# Supplementary material for: Symptom burden and health-related quality of life in chronic kidney disease: A global systematic review and meta-analysis
Source: PLoS Med. 2022 Apr 6;19(4):e1003954. doi: 10.1371/journal.pmed.1003954 (PMC8985967; doi:10.1371/journal.pmed.1003954)
Supplement: S2 Appendix — (DOCX) [file pmed.1003954.s002.docx]

**Appendix 2 – Ovid MEDLINE search strategy**

1. (Symptom* AND CKD).ti.

2. (Symptom* AND chronic kidney disease).ti.

3. (Symptom* AND end-stage renal failure).ti.

4. (Symptom* AND (end-stage kidney disease OR kidney disease)).ti.

5. (Symptom* AND (haemodialysis or peritoneal dialysis)).ti.

6. (Symptom burden AND renal failure).ti.

7. (Symptom burden instrument AND kidney failure).ti.

8. 1 OR 2 OR 3 OR 4 OR 5 OR 6 OR 7

9. ((Quality of life OR QOL) AND CKD).ti.

10. ((Quality of life OR QOL) AND chronic kidney disease).ti.

11. ((Quality of life OR QOL) AND end-stage renal failure).ti.

12. ((Quality of life OR QOL) AND (end-stage kidney disease OR kidney disease)).ti.

13. ((Quality of life OR QOL) AND (haemodialysis or peritoneal dialysis)).ti.

14. ((Quality of life OR QOL) AND renal failure).ti.

15. ((Quality of life OR QOL) AND kidney failure).ti.

16. 9 OR 10 OR 11 OR 12 OR 13 OR 14 OR 15

17. (Adverse event* AND CKD).ti.

18. (Adverse event* AND chronic kidney disease).ti.

19. (Adverse event* AND end-stage renal failure).ti.

20. (Adverse event* AND (end-stage kidney disease OR kidney disease)).ti.

21. (Adverse event* AND (haemodialysis or peritoneal dialysis)).ti.

22. (Adverse event* AND renal failure).ti.

23. (Adverse event* AND kidney failure).ti.

24. 17 OR 18 OR 19 OR 20 OR 21 OR 22 OR 23 25. 8 OR 16 OR 24
