# Supplementary material for: Symptom burden and health-related quality of life in chronic kidney disease: A global systematic review and meta-analysis
Source: PLoS Med. 2022 Apr 6;19(4):e1003954. doi: 10.1371/journal.pmed.1003954 (PMC8985967; doi:10.1371/journal.pmed.1003954)
Supplement: S4 Appendix — (DOCX) [file pmed.1003954.s004.docx]

**Appendix 3 – List of included studies**

1. Aasebo W, Homb-Vesteraas NA, Hartmann A, Stavem K. Life situation and quality of life in young adult kidney transplant recipients. Nephrol Dial Transplant. 2009;24(1):304-8.

2. Abaci SH, Alagoz S, Salihoglu A, Yalin SF, Gulcicek S, Altiparmak MR, et al. Assessment of Anemia and Quality of Life in Patients With Renal Transplantation. Transplant Proc. 2015;47(10):2875-80.

3. Abdel-Kader K, Myaskovsky L, Karpov I, Shah J, Hess R, Dew MA, et al. Individual quality of life in chronic kidney disease: influence of age and dialysis modality. Clin J Am Soc Nephrol. 2009;4(4):711-8.

4. Abdel-Kader K, Unruh ML, Weisbord SD. Symptom burden, depression, and quality of life in chronic and end-stage kidney disease. Clin J Am Soc Nephrol. 2009;4(6):1057-64.

5. Abeywickrama HM, Wimalasiri S, Koyama Y, Uchiyama M, Shimizu U, Kakihara N, et al. Quality of Life and Symptom Burden among Chronic Kidney Disease of Uncertain Etiology (CKDu) Patients in Girandurukotte, Sri Lanka. International Journal of Environmental Research & Public Health [Electronic Resource]. 2020;17(11):05.

6. Acaray A, Pinar R. Quality of life in Turkish haemodialysis patients. Int Urol Nephrol. 2005;37(3):595-602.

7. Acaray A, Pinar R. Quality of life perceived by chronic haemodialysis patients and family members. EDTNA ERCA J. 2005;31(1):35-8.

8. Adejumo O, Akinbodewa A, Alli O, Olatunji A, Ibukun I. Prevalence, Pattern and Association of Pruritus with Quality of Life in Chronic Kidney Disease Patients Attending Kidney Care Centre, Ondo City, Southwest Nigeria. Ethiop J Health Sci. 2016;26(6):549-54.

9. Ademola BL, Obiagwu PN, Aliyu A. Assessment of health-related quality of life of chronic kidney disease patients in aminu kano teaching hospital, Kano. Niger J Clin Pract. 2020;23(7):906-11.

10. Afsar B, Elsurer R, Yilmaz MI, Eyileten T, Yenicesu M. Irritable bowel syndrome in haemodialysis: Prevalence, link with quality of life and depression. Nephrology (Carlton). 2010;15(2):197-202.

11. Afshar M, Rebollo-Mesa I, Murphy E, Murtagh FE, Mamode N. Symptom burden and associated factors in renal transplant patients in the U.K. J Pain Symptom Manage. 2012;44(2):229-38.

12. Aggarwal HK, Jain D, Pawar S, Yadav RK. Health-related quality of life in different stages of chronic kidney disease. QJM. 2016;109(11):711-6.

13. Ajeebi A, Saeed A, Aljamaan A, Alshehri M, Nasradeen M, Alharbi N, et al. A study of quality of life among hemodialysis patients and its associated factors using kidney disease quality of life instrument-SF36 in Riyadh, Saudi Arabia. Saudi J Kidney Dis Transpl. 2020;31(6):1225-33.

14. Akman B, Uyar M, Afsar B, Sezer S, Ozdemir FN, Haberal M. Adherence, depression and quality of life in patients on a renal transplantation waiting list. Transpl Int. 2007;20(8):682-7.

15. Al Salmi I, Kamble P, Lazarus ER, D'Souza MS, Al Maimani Y, Hannawi S. Kidney Disease-Specific Quality of Life among Patients on Hemodialysis. International Journal of Nephrology. 2021;2021:8876559.

16. Al Wakeel J, Al Harbi A, Bayoumi M, Al-Suwaida K, Al Ghonaim M, Mishkiry A. Quality of life in hemodialysis and peritoneal dialysis patients in Saudi Arabia. Ann Saudi Med. 2012;32(6):570-4.

17. Al Wakeel J, Bayoumi M, Al Suwaida A, Al Harbi A, Askar A, Mishriky A. Influences on quality of life in peritoneal dialysis patients. Renal Society of Australasia Journal. 2009;5(3):127-32.

18. Al-Nashri F, Almutary H. Impact of anxiety and depression on the quality of life of haemodialysis patients. J Clin Nurs. 2021:No Pagination Specified.

19. AlDukhayel A. Prevalence of Depressive Symptoms among Hemodialysis and Peritoneal Dialysis Patients. Int J Health Sci (Qassim). 2015;9(1):9-16.

20. Alkatheri A, Al Bekairy A, Aburuz S, Qandil A, Khalidi N, Abdullah K, et al. Exploring quality of life among renal and liver transplant recipients. Ann Saudi Med. 2015;35(5):368-76.

21. Almutary H, Bonner A, Douglas C. Which Patients with Chronic Kidney Disease Have the Greatest Symptom Burden? A Comparative Study of Advanced Ckd Stage and Dialysis Modality. J Ren Care. 2016;42(2):73-82.

22. Alshraifeen A, Al-Rawashdeh S, Alnuaimi K, Alzoubi F, Tanash M, Ashour A, et al. Social support predicted quality of life in people receiving haemodialysis treatment: A cross-sectional survey. Nursing Open. 2020;7(5):1517-25.

23. Alshraifeen A, Al-Rawashdeh S, Herth K, Alnuaimi K, Alzoubi F, Khraim F, et al. The association between hope and quality of life in haemodialysis patients. Br J Nurs. 2020;29(21):1260-5.

24. Alshraifeen A, McCreaddie M, Evans JM. Quality of life and well-being of people receiving haemodialysis treatment in Scotland: a cross-sectional survey. Int J Nurs Pract. 2014;20(5):518-23.

25. Alves KB, Guilarducci NV, Santos TDR, Baldoni AO, Otoni A, Pinto SWL, et al. Is quality of life associated with compliance to pharmacoterapy in patients with chronic kidney disease undergoing maintenance hemodialysis? Einstein (Sao Paulo). 2018;16(1):eAO4036.

26. Amira O. Prevalence of symptoms of depression among patients with chronic kidney disease. Niger J Clin Pract. 2011;14(4):460-3.

27. Andrade A, Amorim A, Queiroz S, Gordon-Nunez M, Freitas R, Galvao H. Comparison of Oral Health Status and the Quality of Life in Haemodialysis Patients with Less and More than Four Years of Treatment. Oral Health Prev Dent. 2017;15(1):57-64.

28. Antunes AV, Mota Sousa LM, Justo C, Ferrer J, Frade F, Silva Pedro Severino S, et al. Assessment of the perceived quality of life of a kidney transplant patient. Enfermería Nefrológica. 2018;21(2):138-44.

29. Aoun M, Sleilaty G, Antoun L, Dib R, Chelala D. Duchenne Smile is Associated with Quality of Life and Survival in Hemodialysis Patients. Am J Health Behav. 2020;44(3):313-25.

30. Arenas VG, Barros LFNM, Lemos FB, Martins MA, David-Neto E. Quality of Life: comparison between patients on automated peritoneal dialysis and patients on hemodialysis. Acta Paulista De Enfermagem. 2009;22:535-9.

31. Artzi-Medvedik R, Kob R, Fabbietti P, Lattanzio F, Corsonello A, Melzer Y, et al. Impaired kidney function is associated with lower quality of life among community-dwelling older adults : The screening for CKD among older people across Europe (SCOPE) study. BMC Geriatr. 2020;20(Suppl 1):340.

32. Atalay H, Solak Y, Biyik M, Biyik Z, Yeksan M, Uguz F, et al. Sertraline treatment is associated with an improvement in depression and health-related quality of life in chronic peritoneal dialysis patients. Int Urol Nephrol. 2010;42(2):527-36.

33. Atapour A, Nasr S, Boroujeni AM, Taheri D, Dolatkhah S. A comparison of the quality of life of the patients undergoing hemodialysis versus peritoneal dialysis and its correlation to the quality of dialysis. Saudi J Kidney Dis Transpl. 2016;27(2):270-80.

34. Auneau-Enjalbert L, Hardouin JB, Blanchin M, Giral M, Morelon E, Cassuto E, et al. Comparison of longitudinal quality of life outcomes in preemptive and dialyzed patients on waiting list for kidney transplantation. Qual Life Res. 2019:No Pagination Specified.

35. Ayoub A, Nelson K, Wood P, Hijjazi KH. Comparing health-related quality of life between haemodialysis patients and a community sample in the United Arab Emirates. Renal Society of Australasia Journal. 2014;10(1):34-43.

36. Azevedo P, Santos R, Duraes J, Santos O, M JC, Cabrita A, et al. Sexual dysfunction in men and women on peritoneal dialysis: Differential link with metabolic factors and quality of life perception. Nefrologia. 2014;34(6):703-9.

37. Bagasha P, Namukwaya E, Leng M, Kalyesubula R, Mutebi E, Naitala R, et al. Comparison of the health-related quality of life of end stage kidney disease patients on hemodialysis and non-hemodialysis management in Uganda. BMC Palliat Care. 2021;20(1):52.

38. Bakewell AB, Higgins RM, Edmunds ME. Quality of life in peritoneal dialysis patients: decline over time and association with clinical outcomes. Kidney Int. 2002;61(1):239-48.

39. Balaska A, Moustafellos P, Gourgiotis S, Pistolas D, Hadjiyannakis E, Vougas V, et al. Changes in health-related quality of life in Greek adult patients 1 year after successful renal transplantation. Exp Clin Transplant. 2006;4(2):521-4.

40. Balasubramanian G, McKitty K, Fan SL. Comparing automated peritoneal dialysis with continuous ambulatory peritoneal dialysis: survival and quality of life differences? Nephrol Dial Transplant. 2011;26(5):1702-8.

41. Barbosa JBN, Cruz de Moura ECS, Barboza de Lira CLO, de Melo Marinho PÉ. Quality of life and duration of hemodialysis in patients with chronic kidney disease (CKD): a cross-sectional study. Fisioterapia em Movimento. 2017;30(4):781-8.

42. Basok EK, Atsu N, Rifaioglu MM, Kantarci G, Yildirim A, Tokuc R. Assessment of female sexual function and quality of life in predialysis, peritoneal dialysis, hemodialysis, and renal transplant patients. Int Urol Nephrol. 2009;41(3):473-81.

43. Battaglia Y, Zerbinati L, Piazza G, Martino E, Provenzano M, Esposito P, et al. Screening Performance of Edmonton Symptom Assessment System in Kidney Transplant Recipients. Journal of Clinical Medicine. 2020;9(4):02.

44. Bayin Donar G, Top M. A conceptual framework of quality of life in chronic kidney disease in Turkey: A patient-focused approach. Int J Health Plann Manage. 2020;35(6):1335-50.

45. Baykan H, Yargic I. Depression, Anxiety Disorders, Quality of Life and Stress Coping Strategies in Hemodialysis and Continuous Ambulatory Peritoneal Dialysis Patients. Klinik Psikofarmakoloji Bulteni-Bulletin of Clinical Psychopharmacology. 2012;22(2):167-76.

46. Bergmann TK, Isbel NM, Ostini R, Barraclough KA, Campbell SB, McWhinney BC, et al. Exploratory study of total and free prednisolone plasma exposure and cushingoid appearance, quality of life and biochemical toxicity in adult male kidney transplant recipients. Clin Drug Investig. 2015;35(11):743-50.

47. Berman N, Christianer K, Roberts J, Feldman R, Reid MC, Shengelia R, et al. Disparities in symptom burden and renal transplant eligibility: a pilot study. J Palliat Med. 2013;16(11):1459-65.

48. Bilgic A, Akman B, Sezer S, Arat Z, Ozelsancak R, Ozdemir N. Daytime sleepiness and quality of life in peritoneal dialysis patients. Ther Apher Dial. 2011;15(6):565-71.

49. Bilgic A, Akman B, Sezer S, Ozisik L, Arat Z, Ozdemir FN, et al. Predictors for quality of life in continuous ambulatory peritoneal dialysis patients. Nephrology (Carlton). 2008;13(7):587-92.

50. Bipath P, Govender C, Viljoen M. A Comparison of Quality of Life in Haemodialysis and Peritoneal Dialysis Patients. Journal of Psychology in Africa. 2008;18(4):625-30.

51. Bittencourt ZZ, Alves Filho G, Mazzali M, Santos NR. [Quality of life in renal transplant patients: impact of a functioning graft]. Rev Saude Publica. 2004;38(5):732-4.

52. Bohlke M, Marini SS, Rocha M, Terhorst L, Gomes RH, Barcellos FC, et al. Factors associated with health-related quality of life after successful kidney transplantation: a population-based study. Qual Life Res. 2009;18(9):1185-93.

53. Bohlke M, Rocha M, Gomes RH, Marini SS, Terhorst L, Barcellos FC, et al. Tacrolimus and quality of life after kidney transplantation--a multicenter study. Clin Transplant. 2006;20(4):504-8.

54. Brennan F, Collett G, Josland EA, Brown MA. The symptoms of patients with CKD stage 5 managed without dialysis. Progress in Palliative Care. 2015;23(5):267-73.

55. Brown EA, Johansson L, Farrington K, Gallagher H, Sensky T, Gordon F, et al. Broadening Options for Long-term Dialysis in the Elderly (BOLDE): differences in quality of life on peritoneal dialysis compared to haemodialysis for older patients. Nephrol Dial Transplant. 2010;25(11):3755-63.

56. Brown MA, Collett GK, Josland EA, Foote C, Li Q, Brennan FP. CKD in elderly patients managed without dialysis: survival, symptoms, and quality of life. Clin J Am Soc Nephrol. 2015;10(2):260-8.

57. Brown SA, Tyrer FC, Clarke AL, Lloyd-Davies LH, Stein AG, Tarrant C, et al. Symptom burden in patients with chronic kidney disease not requiring renal replacement therapy. Clin Kidney J. 2017;10(6):788-96.

58. Brys ADH, Bossola M, Lenaert B, Biamonte F, Gambaro G, Di Stasio E. Daily physical activity in patients on chronic haemodialysis and its relation with fatigue and depressive symptoms. Int Urol Nephrol. 2020;52(10):1959-67.

59. Brys ADH, Di Stasio E, Lenaert B, Sanguinetti M, Picca A, Calvani R, et al. Serum interleukin-6 and endotoxin levels and their relationship with fatigue and depressive symptoms in patients on chronic haemodialysis. Cytokine. 2020;125:154823.

60. Calia R, Lai C, Aceto P, Pascolo G, Lai S, Romagnoli J, et al. Emotional management and quality of life in mother living versus multi-organ donor renal transplant recipients. J Health Psychol. 2017;22(4):475-82.

61. Cano AE, Neil AK, Kang JY, Barnabas A, Eastwood JB, Nelson SR, et al. Gastrointestinal symptoms in patients with end-stage renal disease undergoing treatment by hemodialysis or peritoneal dialysis. Am J Gastroenterol. 2007;102(9):1990-7.

62. Cetingok M, Winsett RP, Hathaway DK. A comparative study of quality of life among the age groups of kidney transplant recipients. Prog Transplant. 2004;14(1):33-8.

63. Ch’ng AS, Chen LL, Lim SK, Poi PJ, Ch'ng AS, Chen LL, et al. Haemodialysis Versus Non-Dialysis Therapy Among Older Adults With Stage 5 Chronic Kidney Disease: A Comparison of Health-Related Quality of Life. Value Health. 2015;18(7):A745-A.

64. Chan W, Chin SH, Whittaker AC, Jones D, Kaur O, Bosch JA, et al. The Associations of Muscle Strength, Muscle Mass, and Adiposity With Clinical Outcomes and Quality of Life in Prevalent Kidney Transplant Recipients. J Ren Nutr. 2019;29(6):536-47.

65. Chen JB, Lam KK, Su YJ, Lee WC, Cheng BC, Kuo CC, et al. Relationship between Kt/V urea-based dialysis adequacy and nutritional status and their effect on the components of the quality of life in incident peritoneal dialysis patients. BMC Nephrol. 2012;13:39.

66. Chen JY, Wan EYF, Choi EPH, Chan AKC, Chan KHY, Tsang JPY, et al. The Health-Related Quality of Life of Chinese Patients on Hemodialysis and Peritoneal Dialysis. Patient. 2017;10(6):799-808.

67. Chen L, Wang J, Huang X, Wang F, Liang W, He Y, et al. Association between diabetes mellitus and health-related quality of life among patients with chronic kidney disease: results from the Chinese Cohort Study of Chronic Kidney Disease (C-STRIDE). Health & Quality of Life Outcomes. 2020;18(1):1-8.

68. Chen W, Chen C, Lee P, Wang W. Quality of life, symptom distress, and social support among renal transplant recipients in Southern Taiwan: a correlational study. Journal of Nursing Research (Taiwan Nurses Association). 2007;15(4):319-29.

69. Chen YC, Lin CL, Lee BO. Relationships of illness representation and quality of life in patients with end‐stage renal disease receiving haemodialysis. Journal of Clinical Nursing (John Wiley & Sons, Inc). 2020;29(19/20):3812-21.

70. Chiang HH, Guo HR, Livneh H, Lu MC, Yen ML, Tsai TY. Increased risk of progression to dialysis or death in CKD patients with depressive symptoms: A prospective 3-year follow-up cohort study. J Psychosom Res. 2015;79(3):228-32.

71. Chilcot J, Wellsted D, Davenport A, Farrington K. Illness representations and concurrent depression symptoms in haemodialysis patients. J Health Psychol. 2011;16(7):1127-37.

72. Chilcot J, Wellsted D, Vilar E, Farrington K. An association between residual renal function and depression symptoms in haemodialysis patients. Nephron Clin Pract. 2009;113(2):c117-24.

73. Chisholm MA, Spivey CA, Nus AV. Influence of economic and demographic factors on quality of life in renal transplant recipients. Clin Transplant. 2007;21(2):285-93.

74. Chisholm-Burns MA, Erickson SR, Spivey CA, Kaplan B. Health-related quality of life and employment among renal transplant recipients. Clin Transplant. 2012;26(3):411-7.

75. Chkhotua A, Pantsulaia T, Managadze L. The quality of life analysis in renal transplant recipients and dialysis patients. Georgian Med News. 2011;11(200):10-7.

76. Christensen AJ, Raichle K, Ehlers SL, Bertolatus AJ. Effect of family environment and donor source on patient quality of life following renal transplantation. Health Psychol. 2002;21(5):468-76.

77. Cleary J, Drennan J. Quality of life of patients on haemodialysis for end-stage renal disease. J Adv Nurs. 2005;51(6):577-86.

78. Coelho de Lima MagalhÃ£es A, ra, Domingos Coelho G, AssumpÃ§Ã£o de Azevedo M, Delacanal Lazzari D, Jung W. QUALITY OF LIFE OF PATIENTS WITH CHRONIC RENAL FAILURE HEMODIALYSIS -TO KIDNEY TRANSPLANT. Journal of Nursing UFPE / Revista de Enfermagem UFPE. 2013;7(9):5442-52.

79. Cohen SD, Patel SS, Khetpal P, Peterson RA, Kimmel PL. Pain, sleep disturbance, and quality of life in patients with chronic kidney disease. Clin J Am Soc Nephrol. 2007;2(5):919-25.

80. Colak H, Sert I, Ekmekci C, Tugmen C, Kurtulmus Y, Kursat S, et al. Correlation of the Volume Control Parameters With Health Related Quality of Life in Renal Transplant Patients. Transplant Proc. 2015;47(5):1369-72.

81. Conde SA, Fernandes N, Santos FR, Chouab A, Mota MM, Bastos MG. Cognitive decline, depression and quality of life in patients at different stages of chronic kidney disease. J Bras Nefrol. 2010;32(3):242-8.

82. Costa JM, Nogueira LT. Association between work, income and quality of life of kidney transplant recipient the municipality of Teresina, PI, Brazil. J Bras Nefrol. 2014;36(3):332-8.

83. Covic A, Seica A, Gusbeth-Tatomir P, Gavrilovici O, Goldsmith DJ. Illness representations and quality of life scores in haemodialysis patients. Nephrol Dial Transplant. 2004;19(8):2078-83.

84. Cruz JP, Colet PC, Alquwez N, Inocian EP, Al-Otaibi RS, Islam SM. Influence of religiosity and spiritual coping on health-related quality of life in Saudi haemodialysis patients. Hemodial Int. 2017;21(1):125-32.

85. Cruz MC, Andrade C, Urrutia M, Draibe S, Nogueira-Martins LA, Sesso Rde C. Quality of life in patients with chronic kidney disease. Clinics (Sao Paulo). 2011;66(6):991-5.

86. Czira ME, Lindner AV, Szeifert L, Molnar MZ, Fornadi K, Kelemen A, et al. Association between the Malnutrition-Inflammation Score and depressive symptoms in kidney transplanted patients. Gen Hosp Psychiatry. 2011;33(2):157-65.

87. Czyzewski L, Frelik P, Wyzgal J, Szarpak L. Evaluation of Quality of Life and Severity of Depression, Anxiety, and Stress in Patients After Kidney Transplantation. Transplant Proc. 2018;50(6):1733-7.

88. Czyzewski L, Sanko-Resmer J, Wyzgal J, Kurowski A. Assessment of health-related quality of life of patients after kidney transplantation in comparison with hemodialysis and peritoneal dialysis. Ann Transplant. 2014;19:576-85.

89. Dano S, Pokarowski M, Liao B, Tang E, Ekundayo O, Li V, et al. Evaluating symptom burden in kidney transplant recipients: validation of the revised Edmonton Symptom Assessment System for kidney transplant recipients - a single-center, cross-sectional study. Transpl Int. 2020;33(4):423-36.

90. Danquah FV, Zimmerman L, Diamond PM, Meininger J, Bergstrom N. Frequency, severity, and distress of dialysis-related symptoms reported by patients on hemodialysis. Nephrol Nurs J. 2010;37(6):627-38; quiz 39.

91. Das RC, Srivastava K, Tudu J, Hooda AK. Crosssectional study of quality of life after renal transplant in end stage renal disease. Ind Psychiatry J. 2014;23(1):40-3.

92. Davison SN, Jhangri GS. Existential and religious dimensions of spirituality and their relationship with health-related quality of life in chronic kidney disease. Clin J Am Soc Nephrol. 2010;5(11):1969-76.

93. Davison SN, Jhangri GS. Impact of pain and symptom burden on the health-related quality of life of hemodialysis patients. J Pain Symptom Manage. 2010;39(3):477-85.

94. Davison SN, Jhangri GS. The Relationship Between Spirituality, Psychosocial Adjustment to Illness, and Health-Related Quality of Life in Patients With Advanced Chronic Kidney Disease. J Pain Symptom Manage. 2013;45(2):170-8.

95. Dawson J, Brennan FP, Hoffman A, Josland E, Li KC, Smyth A, et al. Prevalence of Taste Changes and Association with Other Nutrition-Related Symptoms in End-Stage Kidney Disease Patients. J Ren Nutr. 2021;31(1):80-4.

96. de Groot IB, Veen JI, van der Boog PJ, van Dijk S, Stiggelbout AM, Marang-van de Mheen PJ, et al. Difference in quality of life, fatigue and societal participation between living and deceased donor kidney transplant recipients. Clin Transplant. 2013;27(4):E415-23.

97. de Mendonça AEO, de Vasconcelos Torres G, de Góes Salvetti M, Alchieri JC, Fern, es Costa IK. Changes in Quality of Life after kidney transplantation and related factors. Acta Paulista de Enfermagem. 2013;26(2):287-92.

98. de Oliveira MP, Kusumota L, Marques S, Ribeiro RDHM, Rodrigues RAP, Haas VJ. Work and health-related quality of life of patients on peritoneal dialysis. Acta Paulista De Enfermagem. 2012;25(3):352-7.

99. de Pina Pereira RM, Aparecido Batista M, de Sousa Meira A, Pilotto de Oliveira M, Kusumota L. Quality of life of elderly people with chronic kidney disease in conservative treatment. Rev Bras Enferm. 2017;70(4):851-9.

100. de Wit GA, Merkus MP, Krediet RT, de Charro FT. A comparison of quality of life of patients on automated and continuous ambulatory peritoneal dialysis. Perit Dial Int. 2001;21(3):306-12.

101. Deborah do Couto N, Eduarda Rosado S, Juliana Graciela Vestena Z, Schwartz E, Ângela Jaqueline Sinnott D, Glaucia Jaine Santos da S. QUALITY OF LIFE OF PEOPLE IN PERITONEAL DIALYSIS. Journal of Nursing UFPE / Revista de Enfermagem UFPE. 2017;11(10):4111-7.

102. Diaz-Buxo JA, Lowrie EG, Lew NL, Zhang H, Lazarus JM. Quality-of-life evaluation using Short Form 36: comparison in hemodialysis and peritoneal dialysis patients. Am J Kidney Dis. 2000;35(2):293-300.

103. Dion M, Cristea O, Langford S, Luke PP, Sener A. Debilitating lower urinary tract symptoms in the post-renal transplant population can be predicted pretransplantation. Transplantation. 2013;95(4):589-94.

104. Dong R, Guo ZY. Gastrointestinal symptoms in patients undergoing peritoneal dialysis: multivariate analysis of correlated factors. World J Gastroenterol. 2010;16(22):2812-7.

105. Dong R, Guo ZY, Ding JR, Zhou YY, Wu H. Gastrointestinal symptoms: a comparison between patients undergoing peritoneal dialysis and hemodialysis. World J Gastroenterol. 2014;20(32):11370-5.

106. dos Santos Grincenkov FR, Fernandes N, Chaoubah A, da Silva Fernandes N, Bastos K, Lopes AA, et al. Longitudinal changes in health-related quality of life scores in Brazilian incident peritoneal dialysis patients (BRAZPD): socio-economic status not a barrier. Perit Dial Int. 2013;33(6):687-96.

107. Dweib K, Jumaa S, Khdour M, Hallak H. Quality of life for kidney transplant palestinian patients. Saudi J Kidney Dis Transpl. 2020;31(2):473-81.

108. Einwohner R, Bernardini J, Fried L, Piraino B. The effect of depressive symptoms on survival in peritoneal dialysis patients. Perit Dial Int. 2004;24(3):256-63.

109. Ekberg H, Kyllonen L, Madsen S, Grave G, Solbu D, Holdaas H. Increased prevalence of gastrointestinal symptoms associated with impaired quality of life in renal transplant recipients. Transplantation. 2007;83(3):282-9.

110. Elisabeth Stømer U, Klopstad Wahl A, Gunnar Gøransson L, Hjorthaug Urstad K. Health Literacy in Kidney Disease: Associations with Quality of Life and Adherence. Journal of Renal Care. 2020;46(2):85-94.

111. Eriksson D, Goldsmith D, Teitsson S, Jackson J, van Nooten F. Cross-sectional survey in CKD patients across Europe describing the association between quality of life and anaemia. BMC Nephrol. 2016;17(1):97.

112. Eryilmaz MM, Ozdemir C, Yurtman F, Cilli A, Karaman T. Quality of sleep and quality of life in renal transplantation patients. Transplant Proc. 2005;37(5):2072-6.

113. Esen B, Kahvecioglu S, Atay AE, Ozgen G, Okumus MM, Seyahi N, et al. Evaluation of relationship between sexual functions, depression and quality of life in patients with chronic kidney disease at predialysis stage. Ren Fail. 2015;37(2):262-7.

114. Esposito P, Furini F, Rampino T, Gregorini M, Petrucci L, Klersy C, et al. Assessment of physical performance and quality of life in kidney-transplanted patients: a cross-sectional study. Clin Kidney J. 2017;10(1):124-30.

115. Fan SL, Sathick I, McKitty K, Punzalan S. Quality of life of caregivers and patients on peritoneal dialysis. Nephrol Dial Transplant. 2008;23(5):1713-9.

116. Fardeazar FE, Solhi M, Soola AH, Amani F. Depressive symptoms and associated factors among kidney transplant recipients. Electronic Journal of General Medicine. 2018;15(6):2-8.

117. Fassbinder TR, Winkelmann ER, Schneider J, Wendland J, Oliveira OB. Functional Capacity and Quality of Life in Patients with Chronic Kidney Disease In Pre-Dialytic Treatment and on Hemodialysis--A Cross sectional study. J Bras Nefrol. 2015;37(1):47-54.

118. Favaro Ribeiro D, Helú Mendonça Ribeiro RdC, Sperto Ferreira Baptista MA, Bernardi Cesarino C, Rodrigues CC, im Parra W. DEMOGRAPHIC AND CLINICAL FACTORS ASSOCIATED WITH HEALTH-RELATED QUALITY OF LIFE OF KIDNEY-TRANSPLANTED PATIENTS. Journal of Nursing UFPE / Revista de Enfermagem UFPE. 2016;10(1):57-64.

119. Fayazi S, Asadizaker M, Shahrouz A. Comparison of quality of life between haemodialysis and renal transplant patients. CONNECT: The World of Critical Care Nursing. 2008;6(4):69-72.

120. Feng L, Yap KB, Ng TP. Depressive symptoms in older adults with chronic kidney disease: mortality, quality of life outcomes, and correlates. Am J Geriatr Psychiatry. 2013;21(6):570-9.

121. Fernandez-Jimenez E, Perez-San-Gregorio MA, Martin-Rodriguez A, Perez-Bernal J, Izquierdo G. Evolution of quality of life in renal transplant recipients and patients with multiple sclerosis: a follow-up study. Transplant Proc. 2013;45(10):3616-9.

122. Figueiredo AE, Goodlad C, Clemenger M, Haddoub SS, McGrory J, Pryde K, et al. Evaluation of physical symptoms in patients on peritoneal dialysis. Int J Nephrol. 2012;2012:305424.

123. Fong E, Bargman JM, Chan CT. Cross-sectional comparison of quality of life and illness intrusiveness in patients who are treated with nocturnal home hemodialysis versus peritoneal dialysis. Clin J Am Soc Nephrol. 2007;2(6):1195-200.

124. Fraser SD, Barker J, Roderick PJ, Yuen HM, Shardlow A, Morris JE, et al. Health-related quality of life, functional impairment and comorbidity in people with mild-to-moderate chronic kidney disease: a cross-sectional study. BMJ Open. 2020;10(8):e040286.

125. Fructuoso M, Castro R, Oliveira L, Prata C, Morgado T. Quality of life in chronic kidney disease. Nefrologia. 2011;31(1):91-6.

126. Fujisawa M, Ichikawa Y, Yoshiya K, Isotani S, Higuchi A, Nagano S, et al. Assessment of health-related quality of life in renal transplant and hemodialysis patients using the SF-36 health survey. Urology. 2000;56(2):201-6.

127. Fukushima RLM, Costa JLR, Orl dS, i F. Physical activity and quality of life in chronic kidney disease patients in hemodialysis. Fisioterapia e Pesquisa. 2018;25(3):338-44.

128. Gamondi C, Galli N, Schonholzer C, Marone C, Zwahlen H, Gabutti L, et al. Frequency and severity of pain and symptom distress among patients with chronic kidney disease receiving dialysis. Swiss Med Wkly. 2013;143:w13750.

129. García-Martínez P, Temprado-Albalat MD, Ballester-Arnal R, Gandhi-Morar K, Castro-Calvo J, Collado-Boira E. Predictive model of variables associated with health-related quality of life in patients with advanced chronic kidney disease receiving hemodialysis. Qual Life Res. 2020;29(7):1817-27.

130. Gentile S, Beauger D, Speyer E, Jouve E, Dussol B, Jacquelinet C, et al. Factors associated with health-related quality of life in renal transplant recipients: results of a national survey in France. Health Qual Life Outcomes. 2013;11:88.

131. Ghafourifard M, Mehrizade B, Hassankhani H, Heidari M. Hemodialysis patients perceived exercise benefits and barriers: the association with health-related quality of life. BMC Nephrol. 2021;22(1):1-9.

132. Ginieri-Coccossis M, Theofilou P, Synodinou C, Tomaras V, Soldatos C. Quality of life, mental health and health beliefs in haemodialysis and peritoneal dialysis patients: investigating differences in early and later years of current treatment. BMC Nephrol. 2008;9:14.

133. Goedendorp MM, Hoitsma AJ, Bloot L, Bleijenberg G, Knoop H. Severe fatigue after kidney transplantation: a highly prevalent, disabling and multifactorial symptom. Transpl Int. 2013;26(10):1007-15.

134. Goncalves FA, Dalosso IF, Borba JM, Bucaneve J, Valerio NM, Okamoto CT, et al. Quality of life in chronic renal patients on hemodialysis or peritoneal dialysis: a comparative study in a referral service of Curitiba - PR. J Bras Nefrol. 2015;37(4):467-74.

135. Gorodetskaya I, Zenios S, McCulloch CE, Bostrom A, Hsu CY, Bindman AB, et al. Health-related quality of life and estimates of utility in chronic kidney disease. Kidney Int. 2005;68(6):2801-8.

136. Griva K, Davenport A, Newman SP. Health-related quality of life and long-term survival and graft failure in kidney transplantation: a 12-year follow-up study. Transplantation. 2013;95(5):740-9.

137. Griva K, Goh CS, Kang WCA, Yu ZL, Chan MC, Wu SY, et al. Quality of life and emotional distress in patients and burden in caregivers: a comparison between assisted peritoneal dialysis and self-care peritoneal dialysis. Qual Life Res. 2016;25(2):373-84.

138. Griva K, Kang AW, Yu ZL, Mooppil NK, Foo M, Chan CM, et al. Quality of life and emotional distress between patients on peritoneal dialysis versus community-based hemodialysis. Qual Life Res. 2014;23(1):57-66.

139. Griva K, Stygall J, Ng JH, Davenport A, Harrison MJ, Newman S. Prospective Changes in Health-Related Quality of Life and Emotional Outcomes in Kidney Transplantation over 6 Years. J Transplant. 2011;2011:671571.

140. Griva K, Yu Z, Chan S, Krisnasamy T, Yamin RB, Zakaria FB, et al. Age is not a contraindication to home-based dialysis - Quality-of-Life outcomes favour older patients on peritoneal dialysis regimes relative to younger patients. J Adv Nurs. 2014;70(8):1902-14.

141. Griva K, Ziegelmann JP, Thompson D, Jayasena D, Davenport A, Harrison M, et al. Quality of life and emotional responses in cadaver and living related renal transplant recipients. Nephrol Dial Transplant. 2002;17(12):2204-11.

142. Guenzani D, Buoli M, Caldiroli L, Carnevali GS, Serati M, Vezza C, et al. Malnutrition and inflammation are associated with severity of depressive and cognitive symptoms of old patients affected by chronic kidney disease. J Psychosom Res. 2019;124:109783.

143. Gumprecht J, Zelobowska K, Gosek K, Zywiec J, Adamski M, Grzeszczak W. Quality of life among diabetic and non-diabetic patients on maintenance haemodialysis. Exp Clin Endocrinol Diabetes. 2010;118(3):205-8.

144. Gunalay S, Ozturk YK, Akar H, Mergen H. The relationship between malnutrition and quality of life in haemodialysis and peritoneal dialysis patients. Rev Assoc Med Bras (1992). 2018;64(9):845-52.

145. Guney I, Solak Y, Atalay H, Yazici R, Altintepe L, Kara F, et al. Comparison of effects of automated peritoneal dialysis and continuous ambulatory peritoneal dialysis on health-related quality of life, sleep quality, and depression. Hemodial Int. 2010;14(4):515-22.

146. Gungor O, Kircelli F, Turan MN, Cetin O, Elbi H, Tatar E, et al. Irritable bowel syndrome in renal transplant patients: prevalence, link with quality of life, anxiety, and depression. Ren Fail. 2012;34(7):876-9.

147. Gutierrez Sanchez D, Leiva-Santos JP, Cuesta-Vargas AI. Symptom Burden Clustering in Chronic Kidney Disease Stage 5. Clin Nurs Res. 2019;28(5):583-601.

148. Gyamlani G, Basu A, Geraci S, Lee F, Moxey M, Clark M, et al. Depression, screening and quality of life in chronic kidney disease. Am J Med Sci. 2011;342(3):186-91.

149. Hakamaki M, Lankinen R, Hellman T, Koivuviita N, Parkka JP, Saarenhovi M, et al. Quality of Life Is Associated with Cardiac Biomarkers, Echocardiographic Indices, and Mortality in CKD Stage 4-5 Patients Not on Dialysis. Blood Purif. 2021;50(3):347-54.

150. Harris SA, Lamping DL, Brown EA, Constantinovici N, North Thames Dialysis Study G. Clinical outcomes and quality of life in elderly patients on peritoneal dialysis versus hemodialysis. Perit Dial Int. 2002;22(4):463-70.

151. Hayashino Y, Fukuhara S, Akiba T, Akizawa T, Asano Y, Saito S, et al. Low health-related quality of life is associated with all-cause mortality in patients with diabetes on haemodialysis: the Japan Dialysis Outcomes and Practice Pattern Study. Diabet Med. 2009;26(9):921-7.

152. Hee-Yeon J, Hye Min J, Yang Wook K, Seong C, Hye-Young K, Sung-Ho K, et al. Depressive Symptoms, Patient Satisfaction, and Quality of Life Over Time in Automated and Continuous Ambulatory Peritoneal Dialysis Patients A Prospective Multicenter Propensity-Matched Study. Medicine. 2016;95(21):1-10.

153. Hernandez Sanchez S, Carrero JJ, Garcia Lopez D, Herrero Alonso JA, Menendez Alegre H, Ruiz JR. [Fitness and quality of life in kidney transplant recipients: case-control study]. Med Clin (Barc). 2016;146(8):335-8.

154. Higuita-Gutierrez LF, Velasco-Castano JJ, Jimenez Quiceno JN. Health-Related Quality of Life in Patients with Chronic Kidney Disease in Hemodialysis in Medellin (Colombia). Patient Prefer Adherence. 2019;13:2061-70.

155. Hill KE, Kim S, Crail S, Elias TJ, Whittington T. A comparison of self-reported quality of life for an Australian haemodialysis and haemodiafiltration cohort. Nephrology (Carlton). 2017;22(8):624-30.

156. Hiramatsu T, Okumura S, Asano Y, Mabuchi M, Iguchi D, Furuta S. Quality of Life and Emotional Distress in Peritoneal Dialysis and Hemodialysis Patients. Therapeutic Apheresis & Dialysis: Official Peer-Reviewed Journal of the International Society for Apheresis, the Japanese Society for Apheresis, the Japanese Society for Dialysis Therapy. 2020;24(4):366-72.

157. Ho SE, Ho CC, Norshazwani N, Teoh KH, Ismail MS, Jaafar MZ, et al. Perception of quality of life amongst end stage renal failure patients undergoing haemodialysis. Clin Ter. 2013;164(6):499-505.

158. Hong E, Bernardini J, Fried L, Samsonov M, Pirain B. The relationship between symptoms, depression, and quality of life in peritoneal dialysis patients. Adv Perit Dial. 2006;22:83-7.

159. Hongxia L, Feurer ID, Dwyer K, Speroff T, Shaffer D, Wright Pinson C. The effects of gender and age on health-related quality of life following kidney transplantation. Journal of Clinical Nursing (Wiley-Blackwell). 2008;17(1):82-9.

160. Hoshino J, Muenz D, Zee J, Sukul N, Speyer E, Guedes M, et al. Associations of Hemoglobin Levels With Health-Related Quality of Life, Physical Activity, and Clinical Outcomes in Persons With Stage 3-5 Nondialysis CKD. J Ren Nutr. 2020:N.PAG.

161. Hoth KF, Christensen AJ, Ehlers SL, Raichle KA, Lawton WJ. A longitudinal examination of social support, agreeableness and depressive symptoms in chronic kidney disease. J Behav Med. 2007;30(1):69-76.

162. Houle N, Bohannon RW, Frigon L, Maljanian R, Nieszczezewski J. Health promoting behaviors, quality of life, and hospital resource utilization of patients receiving kidney transplants. Nephrol Nurs J. 2002;29(1):35-40, 56.

163. Huang HC, Walters G, Talaulikar G, Figurski D, Carroll A, Hurwitz M, et al. Sleep apnea prevalence in chronic kidney disease - association with total body water and symptoms. BMC Nephrol. 2017;18(1):125.

164. Hung KC, Wu CC, Chen HS, Ma WY, Tseng CF, Yang LK, et al. Serum IL-6, albumin and co-morbidities are closely correlated with symptoms of depression in patients on maintenance haemodialysis. Nephrol Dial Transplant. 2011;26(2):658-64.

165. Hyodo T, Yamamoto S, Inoguchi Y, Kikuchi C, Sato Y, Oka M, et al. Individual application of the kidney disease quality of life (KDQOL) instrument to monitor the health status of dialysis patients. Nephron. 2000;86(3):391-2.

166. Hyun YY, Lee KB, Chung W, Kim YS, Han SH, Oh YK, et al. Body Mass Index, waist circumference, and health-related quality of life in adults with chronic kidney disease. Qual Life Res. 2019;28(4):1075-83.

167. Ichikawa Y, Fujisawa M, Hirose E, Kageyama T, Miyamoto Y, Sakai Y, et al. Quality of life in kidney transplant patients. Transplant Proc. 2000;32(7):1815-6.

168. Ikonomou M, Skapinakis P, Balafa O, Eleftheroudi M, Damigos D, Siamopoulos KC. The Impact of Socioeconomic Factors on Quality of Life of Patients with Chronic Kidney Disease in Greece. J Ren Care. 2015;41(4):239-46.

169. Iliescu EA, Coo H, McMurray MH, Meers CL, Quinn MM, Singer MA, et al. Quality of sleep and health-related quality of life in haemodialysis patients. Nephrol Dial Transplant. 2003;18(1):126-32.

170. Iqbal MM, Rahman N, Alam M, Deb Nath PK, Waheed S, Islam K, et al. Quality of Life Is Improved in Renal Transplant Recipients Versus That Shown in Patients With Chronic Kidney Disease With or Without Dialysis. Exp Clin Transplant. 2020;18(Suppl 1):64-7.

171. Ishiwatari A, Yamamoto S, Fukuma S, Hasegawa T, Wakai S, Nangaku M. Changes in Quality of Life in Older Hemodialysis Patients: A Cohort Study on Dialysis Outcomes and Practice Patterns. Am J Nephrol. 2020;51(8):650-8.

172. Ismail S, Salamony OE. Evaluation of depression, quality of life and malnutrition-inflammation scores in haemodialysis patients: a cross-sectional analysis. NDT Plus. 2008;1(1):59-60.

173. Iyasere O, Brown E, Gordon F, Collinson H, Fielding R, Fluck R, et al. Longitudinal Trends in Quality of Life and Physical Function in Frail Older Dialysis Patients: A Comparison of Assisted Peritoneal Dialysis and In-Center Hemodialysis. Perit Dial Int. 2019;39(2):112-8.

174. Iyasere O, Brown EA, Johansson L, Davenport A, Farrington K, Maxwell AP, et al. Quality of life with conservative care compared with assisted peritoneal dialysis and haemodialysis. Clin Kidney J. 2019;12(2):262-8.

175. Iyasere OU, Brown EA, Johansson L, Huson L, Smee J, Maxwell AP, et al. Quality of Life and Physical Function in Older Patients on Dialysis: A Comparison of Assisted Peritoneal Dialysis with Hemodialysis. Clin J Am Soc Nephrol. 2016;11(3):423-30.

176. Izbirak G, Akan H, Mistik S, Gundogdu B, Uslu U, Cobanoglu M. Comparison of Health-Related Quality of Life of Patients on Hemodialysis and Continuous Ambulatory Peritoneal Dialysis. Turkiye Klinikleri Tip Bilimleri Dergisi. 2010;30(5):1595-602.

177. Jana AK, Sircar D, Waikhom R, Praharaj SK, ey R, RayChaudhury A, et al. Depression and anxiety as potential correlates of post‑transplantation renal function and quality of life. Indian J Nephrol. 2014;24(5):286-90.

178. Janssen van Doorn K, Heylen M, Mets T, Verbeelen D. Evaluation of functional and mental state and quality of life in chronic haemodialysis patients. Int Urol Nephrol. 2004;36(2):263-7.

179. Jansz TT, Bonenkamp AA, Boereboom FTJ, van Reekum FE, Verhaar MC, van Jaarsveld BC. Health-related quality of life compared between kidney transplantation and nocturnal hemodialysis. PLoS One. 2018;13(9):e0204405.

180. Jesky MD, Dutton M, Dasgupta I, Yadav P, Ng KP, Fenton A, et al. Health-Related Quality of Life Impacts Mortality but Not Progression to End-Stage Renal Disease in Pre-Dialysis Chronic Kidney Disease: A Prospective Observational Study. PLoS One. 2016;11(11):e0165675.

181. Jesus NM, Souza GF, Mendes-Rodrigues C, Almeida Neto OP, Rodrigues DDM, Cunha CM. Quality of life of individuals with chronic kidney disease on dialysis. J Bras Nefrol. 2019;41(3):364-74.

182. Jung HM, Kim HY. A health-related quality of life model for patients undergoing haemodialysis. J Clin Nurs. 2020;29(3-4):613-25.

183. Jung HY, Jeon Y, Park Y, Kim YS, Kang SW, Yang CW, et al. Better Quality of Life of Peritoneal Dialysis compared to Hemodialysis over a Two-year Period after Dialysis Initiation. Sci Rep. 2019;9(1):10266.

184. Kalender B, Ozdemir AC, Dervisoglu E, Ozdemir O. Quality of life in chronic kidney disease: effects of treatment modality, depression, malnutrition and inflammation. Int J Clin Pract. 2007;61(4):569-76.

185. Kaltsouda A, Skapinakis P, Damigos D, Ikonomou M, Kalaitzidis R, Mavreas V, et al. Defensive coping and health-related quality of life in chronic kidney disease: a cross-sectional study. BMC Nephrol. 2011;12:28.

186. Kamau E, Kayima J, Otieno C, Maritim MC, Wanzala P. Health Related Quality of Life of Patients on Maintenance Haemodialysis at Kenyatta National Hospital. East Afr Med J. 2012;89(3):75-81.

187. Kamau E, Kayima J, Otieno CF. The Determinants of Health Related Quality of Life of Patients on Maintenance Haemodialysis at Kenyatta National Hospital, Kenya. East Afr Med J. 2014;91(10):368-74.

188. Kao TW, Tsai DM, Wu KD, Shiah CJ, Hsieh BS, Chen WY. Impact of religious activity on depression and quality of life of chronic peritoneal dialysis patients in Taiwan. J Formos Med Assoc. 2003;102(2):127-30.

189. Karatas A, Canakci E, Turkmen E. Comparison of sleep quality and quality of life indexes with sociodemographic characteristics in patients with chronic kidney disease. Niger J Clin Pract. 2018;21(11):1461-7.

190. Kaynar K, Ozkorumak E, Kural BV, Ulusoy S, Cansiz M, Akcan B, et al. The role of adipocytokines on depressive symptoms of patients with chronic kidney disease. Ren Fail. 2013;35(8):1094-100.

191. Kefale B, Alebachew M, Tadesse Y, Engidawork E. Quality of life and its predictors among patients with chronic kidney disease: A hospital-based cross sectional study. PLoS One. 2019;14(2):e0212184.

192. Kellerman QD, Christensen AJ, Baldwin AS, Lawton WJ. Association Between Depressive Symptoms and Mortality Risk in Chronic Kidney Disease. Health Psychol. 2010;29(6):594-600.

193. Khanh Ngo V, Duangpaeng S, Deenan A, Bonner A. Examining the health-related quality of life of people with end-stage kidney disease living in Hanoi, Vietnam. Renal Society of Australasia Journal. 2012;8(3):140-5.

194. Kim IK, Bae SH, Son S, Kim MS, Jun SY, Ju MK. Health-Related Quality of Life and Psychologic Distress in Korean Kidney Transplant Recipients. Transplant Proc. 2016;48(3):855-7.

195. Kim JH, Han JM, Kim H, Lee KB, Chung W, Kim YS, et al. Low serum adiponectin level is associated with better physical health-related quality of life in chronic kidney disease. Sci Rep. 2021;11(1):10928.

196. Kim O, Yeom EY, Jeon HO. Relationships between depression, family function, physical symptoms, and illness uncertainty in female patients with chronic kidney disease. Nurs Health Sci. 2020;22(3):548-56.

197. Kim S, Nigatu Y, Araya T, Assefa Z, Dereje N. Health related quality of life (HRQOL) of patients with End Stage Kidney Disease (ESKD) on hemodialysis in Addis Ababa, Ethiopia: a cross-sectional study. BMC Nephrol. 2021;22(1):280.

198. Kim YK, Park CS, Ihm SH, Kim HY, Hong TY, Kim DJ, et al. Relationship between the course of depression symptoms and the left ventricular mass index and left ventricular filling pressure in chronic haemodialysis patients. Nephrology (Carlton). 2011;16(2):180-6.

199. Ko B, Khurana A, Spencer J, Scott B, Hahn M, Hammes M. Religious beliefs and quality of life in an American inner-city haemodialysis population. Nephrol Dial Transplant. 2007;22(10):2985-90.

200. Ko GJ, Kim MG, Yu YM, Jo SK, Cho WY, Kim HK. Association between depression symptoms with inflammation and cardiovascular risk factors in patients undergoing peritoneal dialysis. Nephron Clin Pract. 2010;116(1):c29-35.

201. Konel JM, Warsame F, Ying H, Haugen CE, Mountford A, Chu NM, et al. Depressive symptoms, frailty, and adverse outcomes among kidney transplant recipients. Clin Transplant. 2018;32(10):e13391.

202. Kostro JZ, Hellmann A, Kobiela J, Skora I, Lichodziejewska-Niemierko M, Debska-Slizien A, et al. Quality of Life After Kidney Transplantation: A Prospective Study. Transplant Proc. 2016;48(1):50-4.

203. Kovacs AZ, Molnar MZ, Szeifert L, Ambrus C, Molnar-Varga M, Szentkiralyi A, et al. Sleep disorders, depressive symptoms and health-related quality of life--a cross-sectional comparison between kidney transplant recipients and waitlisted patients on maintenance dialysis. Nephrol Dial Transplant. 2011;26(3):1058-65.

204. Krishnan A, Teixeira-Pinto A, Lim WH, Howard K, Chapman JR, Castells A, et al. Health-Related Quality of Life in People Across the Spectrum of CKD. KI Reports. 2020;5(12):2264-74.

205. Kunwar D, Kunwar R, Shrestha B, Amatya R, Risal A. Depression and Quality of Life among the Chronic Kidney Disease Patients. Journal of Nepal Health Research Council. 2020;18(3):459-65.

206. Kustimah, Siswadi AGP, Djunaidi A, Iskandarsyah A. Quality of Life among Patients Undergoing Haemodialysis in Bandung: A Mixed Methods Study. Jurnal Keperawatan Padjadjaran. 2020;8(1):84-92.

207. Lausevic M, Nesic V, Stojanovic M, Stefanovic V. Health-related quality of life in patients on peritoneal dialysis in Serbia: comparison with hemodialysis. Artif Organs. 2007;31(12):901-10.

208. Lazzaretti CT, Carvalho JG, Mulinari RA, Rasia JM. Kidney transplantation improves the multidimensional quality of life. Transplant Proc. 2004;36(4):872-3.

209. Lee HJ, Kwak N, Kim YC, Choi SM, Lee J, Park YS, et al. Impact of Sleep Duration on Mortality and Quality of Life in Chronic Kidney Disease: Results from the 2007-2015 KNHANES. Am J Nephrol. 2021;52(5):396-403.

210. Lee JG, Park MK, Kim YH, Kim YW, Jin K, Park SW, et al. The Correlations among Depressive Symptoms, Cognitive Performance and Serum BDNF Levels in the Patients with Chronic Kidney Disease. Psychiatry Investig. 2018;15(2):186-92.

211. Lee SJ, Jeon J. Relationship between symptom clusters and quality of life in patients at stages 2 to 4 chronic kidney disease in Korea. Appl Nurs Res. 2015;28(4):e13-9.

212. Lee SJ, Son H. Comparison of health-related quality of life between patients with stage 3 and 4 chronic kidney disease and patients undergoing continuous ambulatory peritoneal dialysis. Jpn J Nurs Sci. 2016;13(1):166-73.

213. Lee SJ, Son H, Shin SK. Influence of frailty on health-related quality of life in pre-dialysis patients with chronic kidney disease in Korea: a cross-sectional study. Health Qual Life Outcomes. 2015;13:70.

214. Lee YJ, Kim MS, Cho S, Kim SR. Association of depression and anxiety with reduced quality of life in patients with predialysis chronic kidney disease. Int J Clin Pract. 2013;67(4):363-8.

215. Legrand K, Speyer E, Stengel B, Frimat L, Ngueyon Sime W, Massy ZA, et al. Perceived Health and Quality of Life in Patients With CKD, Including Those With Kidney Failure: Findings From National Surveys in France. Am J Kidney Dis. 2020;75(6):868-78.

216. Lemos CF, Rodrigues MP, Veiga JRP. Family income is associated with quality of life in patients with chronic kidney disease in the pre-dialysis phase: a cross sectional study. Health and Quality of Life Outcomes. 2015;13:1-9.

217. Li J, Wu X, Lin J, Zou D, Yang X, Cheng S, et al. Type D personality, illness perception, social support and quality of life in continuous ambulatory peritoneal dialysis patients. Psychol Health Med. 2017;22(2):196-204.

218. Li Y, Dong J, Zuo L. Is subjective global assessment a good index of nutrition in peritoneal dialysis patients with gastrointestinal symptoms? Perit Dial Int. 2009;29 Suppl 2:S78-82.

219. Li-Chueh W, Yu-Tzu D, Yi-Wen W, Hsiu-Li H, Yang-Jen C. Effects of self-efficacy, self-care behaviours on depressive symptom of Taiwanese kidney transplant recipients. Journal of Clinical Nursing (Wiley-Blackwell). 2008;17(13):1786-94.

220. Lim HA, Yu Z, Kang AW, Foo MW, Griva K. The Course of Quality of Life in Patients on Peritoneal Dialysis: A 12-month Prospective Observational Cohort Study. Int J Behav Med. 2016;23(4):507-14.

221. Lim HJ, Koo TY, Lee J, Huh KH, Park JB, Cho J, et al. Health-Related Quality of Life of Kidney Transplantation Patients: Results from the KoreaN Cohort Study for Outcome in Patients With Kidney Transplantation (KNOW-KT) Study. Transplant Proc. 2016;48(3):844-7.

222. Lin AW, Qian JQ, Yao QA, Gu AP. Quality of life in elderly continuous ambulatory peritoneal dialysis patients. Perit Dial Int. 2003;23 Suppl 2:S95-8.

223. Lin J, Ye H, Yi C, Li J, Yu X, Zhu L, et al. The negative impact of depressive symptoms on patient and technique survival in peritoneal dialysis: a prospective cohort study. Int Urol Nephrol. 2020;52(12):2393-401.

224. Lin YH, Yang MS. A cross-sectional survey of the symptom experience of kidney transplant recipients. Int J Nurs Stud. 2007;44(6):953-60.

225. Lippe N, Waldum-Grevbo B, Reisæter A, Os I. Is HRQOL in dialysis associated with patient survival or graft function after kidney transplantation? BMC Nephrol. 2016;17.

226. Liu HX, Lin J, Lin XH, Wallace L, Teng S, Zhang SP, et al. Quality of sleep and health-related quality of life in renal transplant recipients. Int J Clin Exp Med. 2015;8(9):16191-8.

227. Lockwood MB, Lash JP, Pauls H, Chung SY, Samra M, Ryan C, et al. Physical Symptom Cluster Subgroups in Chronic Kidney Disease. Nurs Res. 2020;69(2):100-8.

228. Lonning K, Midtvedt K, Bernklev T, Brunborg C, Andersen MH, von der Lippe N, et al. Changes in health-related quality of life in older candidates waiting for kidney transplantation. Nephrology (Carlton). 2018;23(10):948-56.

229. Loosman WL, Rottier MA, Honig A, Siegert CE. Association of depressive and anxiety symptoms with adverse events in Dutch chronic kidney disease patients: a prospective cohort study. BMC Nephrol. 2015;16:155.

230. Lopes A, Frade IC, Teixeira L, Almeida M, Dias L, Henriques AC. Quality of life assessment in a living donor kidney transplantation program: evaluation of recipients and donors. Transplant Proc. 2013;45(3):1106-9.

231. Lopes GB, Nogueira FCP, de Souza MR, Penalva MA, de Amorim JL, Pisoni RL, et al. Assessment of the psychological burden associated with pruritus in hemodialysis patients using the kidney disease quality of life short form. Qual Life Res. 2012;21(4):603-12.

232. Lumsdaine JA, Wray A, Power MJ, Jamieson NV, Akyol M, Andrew Bradley J, et al. Higher quality of life in living donor kidney transplantation: prospective cohort study. Transpl Int. 2005;18(8):975-80.

233. Luo L, Chen Q. Effect of CKD-MBD phenotype on health-related quality of life in patients receiving maintenance hemodialysis: A cross-sectional study. J Int Med Res. 2020;48(2):300060519895844.

234. Ma X, Tao M, Hu Y, Tang L, Lu J, Shi Y, et al. Clinical outcomes, quality of life, and costs evaluation of peritoneal dialysis management models in Shanghai Songjiang District: a multi-center and prospective cohort study. Ren Fail. 2021;43(1):754-65.

235. Maglakelidze N, Pantsulaia T, Tchokhonelidze I, Managadze L, Chkhotua A. Assessment of health-related quality of life in renal transplant recipients and dialysis patients. Transplant Proc. 2011;43(1):376-9.

236. Mahato SKS, Apidechkul T, Sriwongpan P, Hada R, Sharma GN, Nayak SK, et al. Factors associated with quality of life among chronic kidney disease patients in Nepal: a cross-sectional study. Health & Quality of Life Outcomes. 2020;18(1):207.

237. Malmstrom RK, Roine RP, Heikkila A, Rasanen P, Sintonen H, Muroma-Karttunen R, et al. Cost analysis and health-related quality of life of home and self-care satellite haemodialysis. Nephrol Dial Transplant. 2008;23(6):1990-6.

238. Manavalan M, Majumdar A, Harichandra Kumar KT, Priyamvada PS. Assessment of health-related quality of life and its determinants in patients with chronic kidney disease. Indian J Nephrol. 2017;27(1):37-43.

239. Manley KJ. Saliva composition and upper gastrointestinal symptoms in chronic kidney disease. J Ren Care. 2014;40(3):172-9.

240. Manns B, Johnson JA, Taub K, Mortis G, Ghali WA, Donaldson C. Quality of life in patients treated with hemodialysis or peritoneal dialysis: what are the important determinants? Clin Nephrol. 2003;60(5):341-51.

241. Mansur HN, Colugnati FA, Grincenkov FR, Bastos MG. Frailty and quality of life: a cross-sectional study of Brazilian patients with pre-dialysis chronic kidney disease. Health Qual Life Outcomes. 2014;12:27.

242. Manu MA, Radulescu S, Harza M, Manu R, Capsa D, Sinescu I. Quality of life assessed by SF-36 health survey in renal transplant patients. Transplant Proc. 2001;33(1-2):1927-8.

243. Marthoenis M, Syukri M, Abdullah A, Tandi TMR, Putra N, Laura H, et al. Quality of life, depression, and anxiety of patients undergoing hemodialysis: Significant role of acceptance of the illness. Int J Psychiatry Med. 2021;56(1):40-50.

244. Martini A, Ammirati A, Garcia C, Andrade C, Portela O, Cendoroglo MS, et al. Evaluation of quality of life, physical, and mental aspects in longevous patients with chronic kidney disease. Int Urol Nephrol. 2018;50(4):725-31.

245. Masina T, Chimera B, Kamponda M, Dreyer G. Health related quality of life in patients with end stage kidney disease treated with haemodialysis in Malawi: a cross sectional study. BMC Nephrol. 2016;17(1):61.

246. Mazairac AH, de Wit GA, Penne EL, van der Weerd NC, de Jong B, Grooteman MP, et al. Changes in quality of life over time--Dutch haemodialysis patients and general population compared. Nephrol Dial Transplant. 2011;26(6):1984-9.

247. Mena Mayayo M, Cerdán Urrutia E, Pérez-Seoane Justo A, Manrique Escola J. Association between functional dependency and affective-depressive symptomatology in patients on the haemodialysis programme. Revista de la Sociedad Española de Enfermería Nefrológica. 2010;13(4):236-41.

248. Metcalfe MS, Tweed A, White SA, Taylor R, Mullin E, Saunders RN, et al. Quality of life for renal transplant recipients of organs from non-heart-beating donors, heart-beating cadaveric donors, and living-related donors. Transplant Proc. 2001;33(7-8):3403-4.

249. Mitsui T, Shimoda N, Morita K, Tanaka H, Moriya K, Nonomura K. Lower urinary tract symptoms and their impact on quality of life after successful renal transplantation. Int J Urol. 2009;16(4):388-92.

250. Mittal SK, Ahern L, Flaster E, Mittal VS, Maesaka JK, Fishbane S. Self-assessed quality of life in peritoneal dialysis patients. Am J Nephrol. 2001;21(3):215-20.

251. Modi GK, Yadav AK, Ghosh A, Kamboj K, Kaur P, Kumar V, et al. Nonmedical Factors and Health-Related Quality of Life in CKD in India. Clinical Journal of The American Society of Nephrology: CJASN. 2020;15(2):191-9.

252. Mok MMY, Liu CKM, Lam MF, Kwan LPY, Chan GCW, Ma MKM, et al. A Longitudinal Study on the Prevalence and Risk Factors for Depression and Anxiety, Quality of Life, and Clinical Outcomes in Incident Peritoneal Dialysis Patients. Perit Dial Int. 2019;39(1):74-82.

253. Mokarram Hossain R, Masud Iqbal M, Rafiqul Alam M, Fazlul Islam S, Omar Faroque M, Islam Selim S. Quality of life in renal transplant recipient and donor. Transplant Proc. 2015;47(4):1128-30.

254. Molnar MZ, Novak M, Szeifert L, Ambrus C, Keszei A, Koczy A, et al. Restless legs syndrome, insomnia, and quality of life after renal transplantation. J Psychosom Res. 2007;63(6):591-7.

255. Molnar-Varga M, Molnar MZ, Szeifert L, Kovacs AZ, Kelemen A, Becze A, et al. Health-related quality of life and clinical outcomes in kidney transplant recipients. Am J Kidney Dis. 2011;58(3):444-52.

256. Moloney FJ, Keane S, O'Kelly P, Conlon PJ, Murphy GM. The impact of skin disease following renal transplantation on quality of life. Br J Dermatol. 2005;153(3):574-8.

257. Molsted S, Prescott L, Heaf J, Eidemak I. Assessment and clinical aspects of health-related quality of life in dialysis patients and patients with chronic kidney disease. Nephron Clin Pract. 2007;106(1):c24-33.

258. Moreira AC, Carolino E, Domingos F, Gaspar A, Ponce P, Camilo ME. Nutritional status influences generic and disease-specific quality of life measures in haemodialysis patients. Nutr Hosp. 2013;28(3):951-7.

259. Morsch CM, Goncalves LF, Barros E. Health-related quality of life among haemodialysis patients - relationship with clinical indicators, morbidity and mortality. J Clin Nurs. 2006;15(4):498-504.

260. Moskovitch JT, Mount PF, Davies MRP. Changes in Symptom Burden in Dialysis Patients Assessed Using a Symptom-Reporting Questionnaire in Clinic. J Palliat Care. 2020;35(1):59-65.

261. Mujais SK, Story K, Brouillette J, Takano T, Soroka S, Franek C, et al. Health-related quality of life in CKD Patients: correlates and evolution over time. Clin J Am Soc Nephrol. 2009;4(8):1293-301.

262. Muñoz Sancho R, Oto Royo A, Barrio Alonso R, Fernández M. Evolution of the quality of life in patients on haemodialysis: one-year prospective study. Revista de la Sociedad Española de Enfermería Nefrológica. 2006;9(1):55-8.

263. Murphy EL, Murtagh FE, Carey I, Sheerin NS. Understanding symptoms in patients with advanced chronic kidney disease managed without dialysis: use of a short patient-completed assessment tool. Nephron Clin Pract. 2009;111(1):c74-80.

264. Murtagh FE, Addington-Hall J, Edmonds P, Donohoe P, Carey I, Jenkins K, et al. Symptoms in the month before death for stage 5 chronic kidney disease patients managed without dialysis. J Pain Symptom Manage. 2010;40(3):342-52.

265. Murtagh FE, Sheerin NS, Addington-Hall J, Higginson IJ. Trajectories of illness in stage 5 chronic kidney disease: a longitudinal study of patient symptoms and concerns in the last year of life. Clin J Am Soc Nephrol. 2011;6(7):1580-90.

266. Murtagh FEM, Addington-Hall JM, Edmonds PM, Donohoe P, Carey I, Jenkins K, et al. Symptoms in advanced renal disease: A cross-sectional survey of symptom prevalence in stage 5 chronic kidney disease managed without dialysis. J Palliat Med. 2007;10(6):1266-76.

267. Myint TM, O'Shaughnessy DV, Marshall S, Vucak-Dzumhur M, Elder GJ. Health-related quality of life of patients awaiting kidney and simultaneous pancreas-kidney transplants. Nephrology (Carlton). 2013;18(12):827-32.

268. Nabolsi MM, Wardam L, Al-Halabi JO. Quality of life, depression, adherence to treatment and illness perception of patients on haemodialysis. Int J Nurs Pract. 2015;21(1):1-10.

269. Neipp M, Karavul B, Jackobs S, Meyer zu Vilsendorf A, Richter N, Becker T, et al. Quality of life in adult transplant recipients more than 15 years after kidney transplantation. Transplantation. 2006;81(12):1640-4.

270. Neto AWG, Boslooper-Meulenbelt K, Geelink M, van Vliet IMY, Post A, Joustra ML, et al. Protein Intake, Fatigue and Quality of Life in Stable Outpatient Kidney Transplant Recipients. Nutrients. 2020;12(8):2451.

271. Ng MSN, So WKW, Wong CL, Hui YH, Ho EHS, Choi KC, et al. Stability and Impact of Symptom Clusters in Patients With End-Stage Renal Disease Undergoing Dialysis. J Pain Symptom Manage. 2020;59(1):67-76.

272. Nguyen HA, Anderson CAM, Miracle CM, Rifkin DE. The Association between Depression, Perceived Health Status, and Quality of Life among Individuals with Chronic Kidney Disease: An Analysis of the National Health and Nutrition Examination Survey 2011-2012. Nephron. 2017;136(2):127-35.

273. Nixon AC, Bampouras TM, Pendleton N, Mitra S, Brady ME, Dhaygude AP. Frailty is independently associated with worse health-related quality of life in chronic kidney disease: a secondary analysis of the Frailty Assessment in Chronic Kidney Disease study. Clinical Kidney Journal. 2020;13(1):85-94.

274. Nixon AC, Wilkinson TJ, Young HML, Taal MW, Pendleton N, Mitra S, et al. Symptom-burden in people living with frailty and chronic kidney disease. BMC Nephrol. 2020;21(1):N.PAG-N.PAG.

275. Noshad H, Sadreddini S, Nezami N, Salekzamani Y, Ardalan MR. Comparison of outcome and quality of life: haemodialysis versus peritoneal dialysis patients. Singapore Med J. 2009;50(2):185-92.

276. Nourbala MH, Hollisaaz MT, Nasiri M, Bahaeloo-Horeh S, Najafi M, Araghizadeh H, et al. Pain affects health-related quality of life in kidney transplant recipients. Transplant Proc. 2007;39(4):1126-9.

277. Novak M, Molnar MZ, Szeifert L, Kovacs AZ, Vamos EP, Zoller R, et al. Depressive symptoms and mortality in patients after kidney transplantation: a prospective prevalent cohort study. Psychosom Med. 2010;72(6):527-34.

278. O'Driscoll CT, House AK, Holman CD. Quality of life measurement using the Short Form-36: a preoperative study of liver and kidney transplantation and living kidney donation in western Australia. Transplant Nurses' Journal. 2008;17(3):32-8.

279. Odden MC, Whooley MA, Shlipak MG. Depression, stress, and quality of life in persons with chronic kidney disease: the Heart and Soul Study. Nephron Clin Pract. 2006;103(1):c1-7.

280. Ogutmen B, Yildirim A, Sever MS, Bozfakioglu S, Ataman R, Erek E, et al. Health-related quality of life after kidney transplantation in comparison intermittent hemodialysis, peritoneal dialysis, and normal controls. Transplant Proc. 2006;38(2):419-21.

281. Oh TR, Kim CS, Bae EH, Ma SK, Han SH, Sung SA, et al. Association between vitamin D deficiency and health-related quality of life in patients with chronic kidney disease from the KNOW-CKD study. PLoS One. 2017;12(4):e0174282.

282. Ok E, Kutlu Y. Hopelessness, Anxiety, Depression and Treatment Adherence in Chronic Hemodialysis Patients. 2020.

283. Okaka EI, Naidoo S, Ahmed MM, Davies M, Naicker S. Quality of life in patients on continuous ambulatory peritoneal dialysis in an African setting. Saudi J Kidney Dis Transpl. 2015;26(3):631-7.

284. Okpechi IG, Nthite T, Swanepoel CR. Health-related quality of life in patients on hemodialysis and peritoneal dialysis. Saudi J Kidney Dis Transpl. 2013;24(3):519-26.

285. Olivera LM, Okuno MFP, Barbosa DA, Sesso RCC, Scherrer Junior G, Pessoa JLE, et al. Quality of life and spirituality of patients with chronic kidney disease: pre- and post-transplant analysis. Rev Bras Enferm. 2020;73 Suppl 5:e20190408.

286. Ong SC, Chow WL, van der Erf S, Joshi VD, Lim JF, Lim C, et al. What factors really matter? Health-related quality of life for patients on kidney transplant waiting list. Ann Acad Med Singapore. 2013;42(12):657-66.

287. Osthus TB, Preljevic V, Sandvik L, Dammen T, Os I. Renal transplant acceptance status, health-related quality of life and depression in dialysis patients. J Ren Care. 2012;38(2):98-106.

288. Overbeck I, Bartels M, Decker O, Harms J, Hauss J, Fangmann J. Changes in quality of life after renal transplantation. Transplant Proc. 2005;37(3):1618-21.

289. Ozcan H, Yucel A, Avsar UZ, Cankaya E, Yucel N, Gozubuyuk H, et al. Kidney Transplantation Is Superior to Hemodialysis and Peritoneal Dialysis in Terms of Cognitive Function, Anxiety, and Depression Symptoms in Chronic Kidney Disease. Transplant Proc. 2015;47(5):1348-51.

290. Ozkayar N, Altun B, Ulusoy S, Yildirim T, Halil M, Yilmaz R, et al. Relationship between Vitamin D Levels and Depressive Symptoms in Renal Transplant Recipients. Int J Psychiatry Med. 2014;47(2):141-51.

291. Pagels AA, Soderkvist BK, Medin C, Hylander B, Heiwe S. Health-related quality of life in different stages of chronic kidney disease and at initiation of dialysis treatment. Health Qual Life Outcomes. 2012;10:71.

292. Pakpour AH, Kumar S, Fridlund B, Zimmer S. A case-control study on oral health-related quality of life in kidney disease patients undergoing haemodialysis. Clin Oral Investig. 2015;19(6):1235-43.

293. Panuccio V, Tripepi R, Bellantoni M, Saporito L, Quattrone S, Lacava V, et al. Pruritus and quality of life in renal transplant patients. Clin Transplant. 2017;31(3):03.

294. Park HC, Lee H, Lee JP, Kim DK, Oh KH, Joo KW, et al. Lower residual renal function is a risk factor for depression and impaired health-related quality of life in Korean peritoneal dialysis patients. J Korean Med Sci. 2012;27(1):64-71.

295. Park JI, Baek H, Jung HH. CKD and Health-Related Quality of Life: The Korea National Health and Nutrition Examination Survey. Am J Kidney Dis. 2016;67(6):851-60.

296. Pauly M, Mateti UV, Shenoy P, Saj N, Philip ML. Dimensions of quality of life in the different stages of chronic kidney disease patients – A cross-sectional study. Clinical Epidemiology and Global Health. 2020;8(3):797-801.

297. Peipert JD, Caicedo JC, Friedewald JJ, Abecassis MMI, Cella D, Ladner DP, et al. Trends and predictors of multidimensional health-related quality of life after living donor kidney transplantation. Qual Life Res. 2020;29(9):2355-74.

298. Peng T, Hu Z, Guo L, Xia Q, Li D, Yang X. Relationship between psychiatric disorders and quality of life in nondialysis patients with chronic kidney disease. Am J Med Sci. 2013;345(3):218-21.

299. Peng YS, Chiang CK, Hung KY, Chiang SS, Lu CS, Yang CS, et al. The association of higher depressive symptoms and sexual dysfunction in male haemodialysis patients. Nephrol Dial Transplant. 2007;22(3):857-61.

300. Peng Z, Wang J, Yuan Q, Xiao X, Xu H, Xie Y, et al. Clinical features and CKD-related quality of life in patients with CKD G3a and CKD G3b in China: results from the Chinese Cohort Study of Chronic Kidney Disease (C-STRIDE). BMC Nephrol. 2017;18(1):311.

301. Perales-Montilla CM, Duschek S, Reyes-Del Paso GA. The influence of emotional factors on the report of somatic symptoms in patients on chronic haemodialysis: the importance of anxiety. Nefrologia. 2013;33(6):816-25.

302. Perales-Montilla CM, Garcia-Leon A, Reyes-del Paso GA. Psychosocial predictors of the quality of life of chronic renal failure patients undergoing haemodialysis. Nefrologia. 2012;32(5):622-30.

303. Perez San Gregorio MA, Martin Rodriguez A, Diaz Dominguez R, Perez Bernal J. [Health related quality of life evolution in kidney transplanted patients]. Nefrologia. 2007;27(5):619-26.

304. Perlman RL, Finkelstein FO, Liu L, Roys E, Kiser M, Eisele G, et al. Quality of life in chronic kidney disease (CKD): a cross-sectional analysis in the Renal Research Institute-CKD study. Am J Kidney Dis. 2005;45(4):658-66.

305. Phair G, Agus A, Normand C, Brazil K, Burns A, Roderick P, et al. Healthcare use, costs and quality of life in patients with end-stage kidney disease receiving conservative management: results from a multi-centre observational study (PACKS). Palliat Med. 2018;32(8):1401-9.

306. Pilger C, Santos R, Lentsck MH, Marques S, Kusumota L. Spiritual well-being and quality of life of older adults in hemodialysis. Rev Bras Enferm. 2017;70(4):689-96.

307. Ponticelli C, Colombo D, Novara M, Basilisco G, Group CS. Gastrointestinal symptoms impair quality of life in Italian renal transplant recipients but are under-recognized by physicians. Transpl Int. 2010;23(11):1126-34.

308. Poppe C, Crombez G, Hanoulle I, Vogelaers D, Petrovic M. Improving quality of life in patients with chronic kidney disease: influence of acceptance and personality. Nephrol Dial Transplant. 2013;28(1):116-21.

309. Portela OT, Belasco AGS, Casarin BF, Lana LD, Nascimento IP, Barbosa DA, et al. Quality of life and cognitive and functional performance of octogenarians and nonagenarians undergoing hemodialysis. Int Urol Nephrol. 2020;52(1):179-85.

310. Porter AC, Lash JP, Xie D, Pan Q, DeLuca J, Kanthety R, et al. Predictors and Outcomes of Health-Related Quality of Life in Adults with CKD. Clin J Am Soc Nephrol. 2016;11(7):1154-62.

311. Prihodova L, Nagyova I, Rosenberger J, Roland R, Groothoff JW, Majernikova M, et al. Health-related quality of life 3 months after kidney transplantation as a predictor of survival over 10 years: a longitudinal study. Transplantation. 2014;97(11):1139-45.

312. Prihodova L, Nagyova I, Rosenberger J, Roland R, van Dijk JP, Groothoff JW. Impact of personality and psychological distress on health-related quality of life in kidney transplant recipients. Transpl Int. 2010;23(5):484-92.

313. Pu L, Zou Y, Wu SK, Wang F, Zhang Y, Li GS, et al. Prevalence and associated factors of depressive symptoms among chronic kidney disease patients in China: Results from the Chinese Cohort Study of Chronic Kidney Disease (C-STRIDE). J Psychosom Res. 2020;128:109869.

314. Pucheu S, Consoli SM, Francais P, Issad B, D'Auzac C. The relationship of quality of life with the severity of disease and non-expression of emotions in peritoneal dialysis. Perit Dial Int. 2004;24(1):77-9.

315. Raimundo P, Ravasco P, Proenca V, Camilo M. Does nutrition play a role in the quality of life of patients under chronic haemodialysis? Nutr Hosp. 2006;21(2):139-44.

316. Raj R, Ahuja KD, Frandsen M, Jose M. Symptoms and their recognition in adult haemodialysis patients: Interactions with quality of life. Nephrology (Carlton). 2017;22(3):228-33.

317. Rambod M, Shabani M, Shokrpour N, Rafii F, Mohammadalliha J. Quality of life of hemodialysis and renal transplantation patients. Health Care Manag (Frederick). 2011;30(1):23-8.

318. Ramos EC, Santos Ida S, Zanini Rde V, Ramos JM. Quality of life of chronic renal patients in peritoneal dialysis and hemodialysis. J Bras Nefrol. 2015;37(3):297-305.

319. Ranabhat K, Khanal P, Mishra SR, Khanal A, Tripathi S, Sigdel MR. Health related quality of life among haemodialysis and kidney transplant recipients from Nepal: a cross sectional study using WHOQOL-BREF. BMC Nephrol. 2020;21(1):N.PAG-N.PAG.

320. Ravindran A, Sunny A, Kunnath RP, Divakaran B. Assessment of Quality of Life among End-Stage Renal Disease Patients Undergoing Maintenance Hemodialysis. Indian J Palliat Care. 2020;26(1):47-53.

321. Rebollo P, Ortega F, Baltar JM, Badia X, Alvarez-Ude F, Diaz-Corte C, et al. Health related quality of life (HRQOL) of kidney transplanted patients: variables that influence it. Clin Transplant. 2000;14(3):199-207.

322. Rebollo P, Ortega F, Valdes C, Fernandez-Vega F, Ortega T, Garcia-Mendoza M, et al. Influence of erectile dysfunction on health related quality of life of male kidney transplant patients. Int J Impot Res. 2004;16(3):282-7.

323. Rehman IU, Chan KG, Munib S, Lee LH, Khan TM. The association between CKD-associated pruritus and quality of life in patients undergoing hemodialysis in Pakistan: A STROBE complaint cross-sectional study. Medicine (Baltimore). 2019;98(36):e16812.

324. Rehman IU, Lai PS, Kun LS, Lee LH, Chan KG, Khan TM. Chronic Kidney Disease-Associated Pruritus and Quality of Life in Malaysian Patients Undergoing Hemodialysis. Ther Apher Dial. 2020;24(1):17-25.

325. Renz Pretto C, Winkelmann ER, Hildebrandt LM, Aparecida Barbosa D, de Fátima Colet C, Fernandes Stumm EM. Quality of life of chronic kidney patients on hemodialysis and related factors. Revista Latino-Americana de Enfermagem (RLAE). 2020;28:1-11.

326. Ricardo AC, Fischer MJ, Peck A, Turyk M, Lash JP. Depressive symptoms and chronic kidney disease: results from the National Health and Nutrition Examination Survey (NHANES) 2005-2006. Int Urol Nephrol. 2010;42(4):1063-8.

327. Rini IS, Rahmayani T, Sari EK, Lestari R. Differences in the quality of life of chronic kidney disease patients undergoing hemodialysis and continuous ambulatory peritoneal dialysis. Journal of Public Health Research. 2021;10(2):14.

328. Rocha G, Poli de Figueiredo CE, d'Avila D, Saitovitch D. Depressive symptoms and kidney transplant outcome. Transplant Proc. 2001;33(7-8):3424.

329. Ronai KZ, Szentkiralyi A, Lazar AS, Lazar ZI, Papp I, Gombos F, et al. Association of symptoms of insomnia and sleep parameters among kidney transplant recipients. J Psychosom Res. 2017;99:95-104.

330. Roscosz Marçal G, da Silva Rêgo A, Paiano M, Trindade Radovanovic CA. Quality of life of patients bearing chronic kidney disease undergoing hemodialysis. Revista de Pesquisa: Cuidado e Fundamental. 2019;11(4):908-13.

331. Russcher M, Nagtegaal JE, Nurmohamed SA, Koch BC, van der Westerlaken MM, van Someren EJ, et al. The effects of kidney transplantation on sleep, melatonin, circadian rhythm and quality of life in kidney transplant recipients and living donors. Nephron. 2015;129(1):6-15.

332. Saffari M, Pakpour AH, Naderi MK, Koenig HG, Baldacchino DR, Piper CN. Spiritual coping, religiosity and quality of life: a study on Muslim patients undergoing haemodialysis. Nephrology (Carlton). 2013;18(4):269-75.

333. Saini T, Murtagh FE, Dupont PJ, McKinnon PM, Hatfield P, Saunders Y. Comparative pilot study of symptoms and quality of life in cancer patients and patients with end stage renal disease. Palliat Med. 2006;20(6):631-6.

334. Samoudi AF, Marzouq MK, Samara AM, Zyoud SeH, Al-Jabi SW. The impact of pain on the quality of life of patients with end-stage renal disease undergoing hemodialysis: a multicenter cross-sectional study from Palestine. Health & Quality of Life Outcomes. 2021;19(1):1-10.

335. Sanner BM, Tepel M, Esser M, Klewer J, Hoehmann-Riese B, Zidek W, et al. Sleep-related breathing disorders impair quality of life in haemodialysis recipients. Nephrol Dial Transplant. 2002;17(7):1260-5.

336. Santos Pereira Bd, Fern S, es Nd, de Melo NP, Abrita R, Santos Grincenkov FRd, et al. Beyond quality of life: a cross sectional study on the mental health of patients with chronic kidney disease undergoing dialysis and their caregivers. Health & Quality of Life Outcomes. 2017;15:1-10.

337. Santos PR. Comparison of quality of life between hemodialysis patients waiting and not waiting for kidney transplant from a poor region of Brazil. J Bras Nefrol. 2011;33(2):166-72.

338. Santos PR, Daher EF, Silva GB, Liborio AB, Kerr LR. Quality of life assessment among haemodialysis patients in a single centre: a 2-year follow-up. Qual Life Res. 2009;18(5):541-6.

339. Santos PR, Franco Sansigolo Kerr LR. Clinical and laboratory variables associated with quality of life in Brazilian haemodialysis patients: a single-centre study. Rev Med Chil. 2008;136(10):1264-71.

340. Saracino A, Gollo I, Di Noia I, Caldone MG, Santarsia G, Procida C, et al. Loss of renal function is associated with deterioration of health-related quality of life in kidney transplant patients. Transplant Proc. 2008;40(10):3460-5.

341. Sayin A, Mutluay R, Sindel S. Quality of life in hemodialysis, peritoneal dialysis, and transplantation patients. Transplant Proc. 2007;39(10):3047-53.

342. Scheel J, Schieber K, Reber S, Jank S, Eckardt KU, Grundmann F, et al. Psychological processing of a kidney transplantation, perceived quality of life, and immunosuppressant medication adherence. Patient Prefer Adherence. 2019;13:775-82.

343. Schick-Makaroff K, Molzahn AE, Kalfoss M. Symptoms, Coping, and Quality of Life of People with Chronic Kidney Disease. Nephrol Nurs J. 2018;45(4):339-55.

344. Schmalz G, Kollmar O, Vasko R, Muller GA, Haak R, Ziebolz D. Oral health-related quality of life in patients on chronic haemodialysis and after kidney transplantation. Oral Dis. 2016;22(7):665-72.

345. Schouten RW, Harmse VJ, Dekker FW, van Ballegooijen W, Siegert CEH, Honig A. Dimensions of Depressive Symptoms and Their Association With Mortality, Hospitalization, and Quality of Life in Dialysis Patients: A Cohort Study. Psychosom Med. 2019;81(7):649-58.

346. Schouten RW, Nadort E, van Ballegooijen W, Loosman WL, Honig A, Siegert CEH, et al. General distress and symptoms of anxiety and depression: A factor analysis in two cohorts of dialysis patients. Gen Hosp Psychiatry. 2020;65:91-9.

347. Schulz T, Niesing J, Homan van der Heide JJ, Westerhuis R, Ploeg RJ, Ranchor AV. Great expectations? Pre-transplant quality of life expectations and distress after kidney transplantation: a prospective study. Br J Health Psychol. 2014;19(4):823-38.

348. Seica A, Segall L, Verzan C, Vaduva N, Madincea M, Rusoiu S, et al. Factors affecting the quality of life of haemodialysis patients from Romania: a multicentric study. Nephrol Dial Transplant. 2009;24(2):626-9.

349. Seidel UK, Gronewold J, Volsek M, Todica O, Kribben A, Bruck H, et al. Physical, cognitive and emotional factors contributing to quality of life, functional health and participation in community dwelling in chronic kidney disease. PLoS One. 2014;9(3):e91176.

350. Senanayake S, Gunawardena N, Palihawadana P, Bandara P, Haniffa R, Karunarathna R, et al. Symptom burden in chronic kidney disease; a population based cross sectional study. BMC Nephrol. 2017;18(1):228.

351. Senanayake S, Gunawardena N, Palihawadana P, Senanayake S, Karunarathna R, Kumara P, et al. Health related quality of life in chronic kidney disease; a descriptive study in a rural Sri Lankan community affected by chronic kidney disease. Health & Quality of Life Outcomes. 2020;18(1):1-9.

352. Senol V, Sipahioglu MH, Ozturk A, Argun M, Utas C. Important determinants of quality of life in a peritoneal dialysis population in Turkey. Ren Fail. 2010;32(10):1196-201.

353. Seow YY, Cheung YB, Qu LM, Yee AC. Trajectory of quality of life for poor prognosis stage 5D chronic kidney disease with and without dialysis. Am J Nephrol. 2013;37(3):231-8.

354. Shah KK, Murtagh FEM, McGeechan K, Crail S, Burns A, Tran AD, et al. Health-related quality of life and well-being in people over 75 years of age with end-stage kidney disease managed with dialysis or comprehensive conservative care: a cross-sectional study in the UK and Australia. BMJ Open. 2019;9(5):e027776.

355. Shahrin FIM, Lim Zhi Y, Omar N, Zakaria NF, Daud ZAM. Association of socio-demographic characteristics, nutritional status, risk of malnutrition and depression with quality of life among elderly haemodialysis patients. Malays J Nutr. 2019;25(1):1-11.

356. Shen Q, Huang X, Luo Z, Xu X, Zhao X, He Q. Sleep quality, daytime sleepiness and health-related quality-of-life in maintenance haemodialysis patients. J Int Med Res. 2016;44(3):698-709.

357. Shen Y, Wang J, Yuan J, Yang L, Yu F, Wang X, et al. Anemia among Chinese patients with chronic kidney disease and its association with quality of life - results from the Chinese cohort study of chronic kidney disease (C-STRIDE). BMC Nephrol. 2021;22(1):1-10.

358. Shim HY, Cho MK. Factors influencing the quality of life of haemodialysis patients according to symptom cluster. J Clin Nurs. 2018;27(9-10):2132-41.

359. Shimoyama S, Hirakawa O, Yahiro K, Mizumachi T, Schreiner A, Kakuma T. Health-related quality of life and caregiver burden among peritoneal dialysis patients and their family caregivers in Japan. Perit Dial Int. 2003;23 Suppl 2:S200-5.

360. Shinozaki G, Jowsey S, Amer H, Biernacka JM, Colby C, Walker D, et al. Relationship between FKBP5 polymorphisms and depression symptoms among kidney transplant recipients. Depress Anxiety. 2011

;28(12):1111-8.

361. Shirazian S, Diep R, Jacobson AM, Grant CD, Mattana J, Calixte R. Awareness of Chronic Kidney Disease and Depressive Symptoms: National Health and Nutrition Examination Surveys 2005-2010. Am J Nephrol. 2016;44(1):1-10.

362. Shrestha A, Shrestha A, Basarab-Horwath C, McKane W, Shrestha B, Raftery A. Quality of life following live donor renal transplantation: a single centre experience. Ann Transplant. 2010;15(2):5-10.

363. Silveira Siqueira D, Pinheiro da Costa BE, Prado Lima Figueiredo AE. Coping and quality of life in patients on kidney transplant waiting lists. Acta Paulista de Enfermagem. 2017;30(6):582-9.

364. Sitjar-Suner M, Suner-Soler R, Masia-Plana A, Chirveches-Perez E, Bertran-Noguer C, Fuentes-Pumarola C. Quality of Life and Social Support of People on Peritoneal Dialysis: Mixed Methods Research. International Journal of Environmental Research & Public Health [Electronic Resource]. 2020;17(12):14.

365. Son YJ, Choi KS, Park YR, Bae JS, Lee JB. Depression, symptoms and the quality of life in patients on hemodialysis for end-stage renal disease. Am J Nephrol. 2009;29(1):36-42.

366. Sousa L, Valentim O, Marques-Vieira C, Antunes AV, Severino S, José H. ASSOCIATION BETWEEN STRESS/ANXIETY, DEPRESSION, PAIN AND QUALITY OF LIFE IN PEOPLE WITH CHRONIC KIDNEY DISEASE. Portuguese Journal of Mental Health Nursing / Revista Portuguesa de Enfermagem de Saude Mental. 2020(23):47-53.

367. Stengel B, Metzger M, Combe C, Jacquelinet C, Briancon S, Ayav C, et al. Risk profile, quality of life and care of patients with moderate and advanced CKD: The French CKD-REIN Cohort Study. Nephrol Dial Transplant. 2019;34(2):277-86.

368. Stomer U, Bergrem H, Goransson LG. Health-related quality of life in kidney transplant patients and non-renal replacement therapy patients with chronic kidney disease stages 3b-4. Ann Transplant. 2013;18:635-42.

369. Suh SH, Choi HS, Kim CS, Bae EH, Ma SK, Lee DH, et al. Chronic kidney disease attenuates the impact of obesity on quality of life. Sci Rep. 2020;10(1):2375.

370. Sung SA, Hyun YY, Lee KB, Park HC, Chung W, Kim YH, et al. Sleep Duration and Health-Related Quality of Life in Predialysis CKD. Clin J Am Soc Nephrol. 2018;13(6):858-65.

371. Surendra NK, Abdul Manaf MR, Hooi LS, Bavanandan S, Mohamad Nor FS, Shah Firdaus Khan S, et al. Health related quality of life of dialysis patients in Malaysia: Haemodialysis versus continuous ambulatory peritoneal dialysis. BMC Nephrol. 2019;20(1):151.

372. Susel J, Batycka-Baran A, Reich A, Szepietowski JC. Uraemic pruritus markedly affects the quality of life and depressive symptoms in haemodialysis patients with end-stage renal disease. Acta Derm Venereol. 2014;94(3):276-81.

373. Suzuki A, Kenmochi T, Maruyama M, Akutsu N, Iwashita C, Otsuki K, et al. Changes in quality of life in deceased versus living-donor kidney transplantations. Transplant Proc. 2012;44(1):287-9.

374. Szeifert L, Molnar MZ, Ambrus C, Koczy AB, Kovacs AZ, Vamos EP, et al. Symptoms of depression in kidney transplant recipients: a cross-sectional study. Am J Kidney Dis. 2010;55(1):132-40.

375. Taghipour B, Mehravar F, Nia HS, Shahidifar S, Hasani SA, Alahyari Z. Association between death anxiety and spiritual intelligence with the spiritual health and quality of life in hemodialysis patients. Journal of Nursing & Midwifery Sciences. 2020;7(2):26-32.

376. Tajima R, Kondo M, Kai H, Saito C, Okada M, Takahashi H, et al. Measurement of health-related quality of life in patients with chronic kidney disease in Japan with EuroQol (EQ-5D). Clin Exp Nephrol. 2010;14(4):340-8.

377. Tanaka M, Ishibashi Y, Hamasaki Y, Kamijo Y, Idei M, Kawahara T, et al. Health-related quality of life on combination therapy with peritoneal dialysis and hemodialysis in comparison with hemodialysis and peritoneal dialysis: A cross-sectional study. Perit Dial Int. 2020;40(5):462-9.

378. Tannor EK, Norman BR, Adusei KK, Sarfo FS, Davids MR, Bedu-Addo G. Quality of life among patients with moderate to advanced chronic kidney disease in Ghana - a single centre study. BMC Nephrol. 2019;20(1):122.

379. Tanriverdi N, Ozcurumez G, Colak T, Duru C, Emiroglu R, Zileli L, et al. Quality of life and mood in renal transplantation recipients, donors, and controls: preliminary report. Transplant Proc. 2004;36(1):117-9.

380. Tao X, Zhang H, Huang J, Gu A, Jin Y, He Y, et al. Physical performance and health-related quality of life among older adults on peritoneal dialysis: a cross-sectional study. Int Urol Nephrol. 2021;53(5):1033-42.

381. Taskintuna N, Ozcurumez G, Duru C, Colak T, Haberal M. Psychosocial aspects of living-related donor renal transplantation: Quality of life and mood in recipients, donors and controls. Int J Psychiatry Clin Pract. 2009;13(3):218-22.

382. Tavallaii SA, Einollahi B, Azizabadi Farahani M, Namdari M. Socioeconomic links to health-related quality of life, anxiety, and depression in kidney transplant recipients. Iran J Kidney Dis. 2009;3(1):40-4.

383. Teles F, Amorim de Albuquerque AL, Freitas Guedes Lins IK, Carvalho Medrado P, Falcao Pedrosa Costa A. Quality of life and depression in haemodialysis patients. Psychol Health Med. 2018;23(9):1069-78.

384. Tesfaye WH, McKercher C, Peterson GM, Castelino RL, Jose M, Zaidi STR, et al. Medication Adherence, Burden and Health-Related Quality of Life in Adults with Predialysis Chronic Kidney Disease: A Prospective Cohort Study. Int J Environ Res Public Health. 2020;17(1):06.

385. Thancharoen O, Waleekhachonloet O, Limwattananon C, Anutrakulchai S. Cognitive impairment, quality of life and healthcare utilization in patients with chronic kidney disease stages 3 to 5. Nephrology. 2020;25(8):625-33.

386. Theofilou P. Quality of life in patients undergoing hemodialysis or peritoneal dialysis treatment. J Clin Med Res. 2011;3(3):132-8.

387. Torino C, Panuccio V, Tripepi R, Vilasi A, Postorino M, Tripepi G, et al. The dominant prognostic value of physical functioning among quality of life domains in end-stage kidney disease. Nephrol Dial Transplant. 2020;35(1):170-5.

388. Tsai YC, Chen HM, Hsiao SM, Chen CS, Lin MY, Chiu YW, et al. Association of physical activity with cardiovascular and renal outcomes and quality of life in chronic kidney disease. PLoS One. 2017;12(8):e0183642.

389. Tsai YC, Chiu YW, Hung CC, Hwang SJ, Tsai JC, Wang SL, et al. Association of symptoms of depression with progression of CKD. Am J Kidney Dis. 2012;60(1):54-61.

390. Tsai YC, Hung CC, Hwang SJ, Wang SL, Hsiao SM, Lin MY, et al. Quality of life predicts risks of end-stage renal disease and mortality in patients with chronic kidney disease. Nephrol Dial Transplant. 2010;25(5):1621-6.

391. Tuot DS, Lin F, Norris K, Gassman J, Smogorzewski M, Ku E. Depressive Symptoms Associate With Race and All-Cause Mortality in Patients With CKD. Kidney Int Rep. 2019;4(2):222-30.

392. Turkmen K, Yazici R, Solak Y, Guney I, Altintepe L, Yeksan M, et al. Health-related quality of life, sleep quality, and depression in peritoneal dialysis and hemodialysis patients. Hemodial Int. 2012;16(2):198-206.

393. Uchiyama K, Washida N, Muraoka K, Morimoto K, Kasai T, Yamaki K, et al. Exercise Capacity and Association with Quality of Life in Peritoneal Dialysis Patients. Perit Dial Int. 2019;39(1):66-73.

394. Uhlinova J, Pechter Ü, Põlluste K, Raag M, Kallikorm R, Lember M, et al. Patient-reported outcomes: association between physical activity and quality of life in patients with chronic kidney disease. Family Medicine & Primary Care Review. 2020;22(4):343-8.

395. van de Luijtgaarden MWM, Caskey FJ, Wanner C, Chesnaye NC, Postorino M, Janmaat CJ, et al. Uraemic symptom burden and clinical condition in women and men of >/=65 years of age with advanced chronic kidney disease: results from the EQUAL study. Nephrol Dial Transplant. 2019;34(7):1189-96.

396. van der Weide MJ, Hilbrands LB, Bemelmans BL, Kiemeney LA. Lower urinary tract symptoms after renal transplantation: are there changes over time? Urology. 2004;63(3):442-6.

397. van der Weide MJ, Hilbrands LB, Bemelmans BL, Meuleman EJ, Frederiks CM. Lower urinary tract symptoms after renal transplantation. J Urol. 2001;166(4):1237-41.

398. van Haalen H, Jackson J, Spinowitz B, Milligan G, Moon R. Impact of chronic kidney disease and anemia on health-related quality of life and work productivity: analysis of multinational real-world data. BMC Nephrol. 2020;21(1):88.

399. van Sandwijk MS, Al Arashi D, van de Hare FM, van der Torren JMR, Kersten MJ, Bijlsma JA, et al. Fatigue, anxiety, depression and quality of life in kidney transplant recipients, haemodialysis patients, patients with a haematological malignancy and healthy controls. Nephrol Dial Transplant. 2019;34(5):833-8.

400. Vanden Wyngaert K, Van Craenenbroeck AH, Eloot S, Calders P, Celie B, Holvoet E, et al. Associations between the measures of physical function, risk of falls and the quality of life in haemodialysis patients: a cross-sectional study. BMC Nephrol. 2020;21(1):7.

401. Varela L, Vazquez MI, Bolanos L, Alonso R. Psychological predictors for health-related quality of life in patients on peritoneal dialysis. Nefrologia. 2011;31(1):97-106.

402. Vero LM, Byham-Gray L, Parrott JS, Steiber AL. Use of the subjective global assessment to predict health-related quality of life in chronic kidney disease stage 5 patients on maintenance hemodialysis. J Ren Nutr. 2013;23(2):141-7.

403. Villeneuve C, Laroche ML, Essig M, Merville P, Kamar N, Coubret A, et al. Evolution and Determinants of Health-Related Quality-of-Life in Kidney Transplant Patients Over the First 3 Years After Transplantation. Transplantation. 2016;100(3):640-7.

404. Virzi A, Signorelli MS, Veroux M, Giammarresi G, Maugeri S, Nicoletti A, et al. Depression and quality of life in living related renal transplantation. Transplant Proc. 2007;39(6):1791-3.

405. Vos PF, Zilch O, Jennekens-Schinkel A, Salden M, Nuyen J, Kooistra MM, et al. Effect of short daily home haemodialysis on quality of life, cognitive functioning and the electroencephalogram. Nephrol Dial Transplant. 2006;21(9):2529-35.

406. Wan EY, Chen JY, Choi EP, Wong CK, Chan AK, Chan KH, et al. Patterns of health-related quality of life and associated factors in Chinese patients undergoing haemodialysis. Health Qual Life Outcomes. 2015;13:108.

407. Wan Zukiman WZH, Yaakup H, Zakaria NF, Shah SAB. Symptom Prevalence and the Negative Emotional States in End-Stage Renal Disease Patients with or without Renal Replacement Therapy: A Cross-Sectional Analysis. J Palliat Med. 2017;20(10):1127-34.

408. Wang AY, Sherrington C, Toyama T, Gallagher MP, Cass A, Hirakawa Y, et al. Muscle strength, mobility, quality of life and falls in patients on maintenance haemodialysis: A prospective study. Nephrology (Carlton). 2017;22(3):220-7.

409. Wang H, Du C, Liu H, Zhang S, Wu S, Fu Y, et al. Exploration of symptom experience in kidney transplant recipients based on symptoms experience model. Qual Life Res. 2020:No Pagination Specified.

410. Wang K, Nguyen M, Chen Y, Hoofnagle AN, Becker JO, Zelnick LR, et al. Association of Tubular Solute Clearance with Symptom Burden in Incident Peritoneal Dialysis. Clinical Journal of The American Society of Nephrology: CJASN. 2020;15(4):530-8.

411. Wang WG, Li P, Wang YS, Wang G, Wang YT, Zhou HL. The effect of erectile dysfunction on quality of life in male kidney transplant recipients. Pak J Med Sci. 2014;30(2):361-5.

412. Wang WL, Liang S, Zhu FL, Liu JQ, Wang SY, Chen XM, et al. The prevalence of depression and the association between depression and kidney function and health-related quality of life in elderly patients with chronic kidney disease: a multicenter cross-sectional study. Clin Interv Aging. 2019;14:905-13.

413. Ware JE, Jr., Richardson MM, Meyer KB, Gandek B. Improving CKD-Specific Patient-Reported Measures of Health-Related Quality of Life. J Am Soc Nephrol. 2019;30(4):664-77.

414. Wasserfallen JB, Halabi G, Saudan P, Perneger T, Feldman HI, Martin PY, et al. Quality of life on chronic dialysis: comparison between haemodialysis and peritoneal dialysis. Nephrol Dial Transplant. 2004;19(6):1594-9.

415. Watanabe Y, Okada H. Effect of Combined Peritoneal Dialysis and Hemodialysis on Health-Related Quality of Life. Contrib Nephrol. 2018;196:135-40.

416. Weber M, Faravardeh A, Jackson S, Berglund D, Spong R, Matas AJ, et al. Quality of life in elderly kidney transplant recipients. J Am Geriatr Soc. 2014;62(10):1877-82.

417. Wee H-L, Seng BJJ, Lee JJ, Chong KJ, Tyagi P, Vathsala A, et al. Association of anemia and mineral and bone disorder with health-related quality of life in Asian pre-dialysis patients. Health and quality of life outcomes. 2016;14:94-.

418. Wei H, Guan Z, Zhao J, Zhang W, Shi H, Wang W, et al. Physical Symptoms and Associated Factors in Chinese Renal Transplant Recipients. Transplant Proc. 2016;48(8):2644-9.

419. Wei TY, Chiang YJ, Hsieh CY, Weng LC, Lin SC, Lin MH. Health related quality of life of long-term kidney transplantation recipients. Biomed J. 2013;36(5):243-51.

420. Weisbord SD, Bossola M, Fried LF, Giungi S, Tazza L, Palevsky PM, et al. Cultural comparison of symptoms in patients on maintenance hemodialysis. Hemodial Int. 2008;12(4):434-40.

421. Weiss M, Mettang T, Tschulena U, Weisshaar E. Health-related quality of life in haemodialysis patients suffering from chronic itch: results from GEHIS (German Epidemiology Haemodialysis Itch Study). Qual Life Res. 2016;25(12):3097-106.

422. Weng LC, Dai YT, Huang HL, Chiang YJ. Self-efficacy, self-care behaviours and quality of life of kidney transplant recipients. J Adv Nurs. 2010;66(4):828-38.

423. White C, Gallagher P. Effect of patient coping preferences on quality of life following renal transplantation. J Adv Nurs. 2010;66(11):2550-9.

424. Wilkinson TJ, Nixon DGD, Palmer J, Lightfoot CJ, Smith AC. Differences in physical symptoms between those with and without kidney disease: a comparative study across disease stages in a UK population. BMC Nephrol. 2021;22(1):147.

425. Wong CKH, Chen JY, Fung SKS, Lo WK, Lui SL, Chan TM, et al. Health-related quality of life and health utility of Chinese patients undergoing nocturnal home haemodialysis in comparison with other modes of dialysis. Nephrology (Carlton). 2019;24(6):630-7.

426. Wong G, Howard K, Chapman J, Pollock C, Chadban S, Salkeld G, et al. How do people with chronic kidney disease value cancer-related quality of life? Nephrology (Carlton). 2012;17(1):32-41.

427. Wong MG, Ninomiya T, Liyanage T, Sukkar L, Hirakawa Y, Wang Y, et al. Physical component quality of life reflects the impact of time and moderate chronic kidney disease, unlike SF-6D utility and mental component SF-36 quality of life: An AusDiab analysis. Nephrology (Carlton). 2019;24(6):605-14.

428. Wozniak I, Kolonko A, Chudek J, Nowak L, Farnik M, Wiecek A. Influence of Polypharmacy on the Quality of Life in Stable Kidney Transplant Recipients. Transplant Proc. 2018;50(6):1896-9.

429. Wright LS, Wilson L. CNE. Quality of Life and Self-Efficacy in Three Dialysis Modalities: Incenter Hemodialysis, Home Hemodialysis, and Home Peritoneal Dialysis. Nephrol Nurs J. 2015;42(5):463-77.

430. Wu AW, Fink NE, Marsh-Manzi JV, Meyer KB, Finkelstein FO, Chapman MM, et al. Changes in quality of life during hemodialysis and peritoneal dialysis treatment: generic and disease specific measures. J Am Soc Nephrol. 2004;15(3):743-53.

431. Wyld MLR, Morton RL, Clayton P, Wong MG, Jardine M, Polkinghorne K, et al. The impact of progressive chronic kidney disease on health-related quality-of-life: a 12-year community cohort study. Qual Life Res. 2019;28(8):2081-90.

432. Xuehan Z, Bansal N, Go AS, Chi-yuan H, Zhang X, Hsu C-Y. Gastrointestinal symptoms, inflammation and hypoalbuminemia in chronic kidney disease patients: a cross-sectional study. BMC Nephrol. 2015;16:1-8.

433. Yagil Y, Geller S, Levy S, Sidi Y, Aharoni S. Body-image, quality of life and psychological distress: a comparison between kidney transplant patients and a matching healthy sample. Psychology Health & Medicine. 2018;23(4):424-33.

434. Yang F, Luo N, Lau T, Yu ZL, Foo MWY, Griva K. Health-Related Quality of Life in Patients Treated with Continuous Ambulatory Peritoneal Dialysis and Automated Peritoneal Dialysis in Singapore. Pharmacoecon Open. 2018;2(2):203-8.

435. Yildirim A. The importance of patient satisfaction and health-related quality of life after renal transplantation. Transplant Proc. 2006;38(9):2831-4.

436. Yoon HE, Kwon YJ, Song HC, Kim JK, Song YR, Shin SJ, et al. Overhydration Negatively Affects Quality of Life in Peritoneal Dialysis Patients: Evidence from a Prospective Observational Study. Int J Med Sci. 2016;13(9):686-95.

437. Yu IC, Huang JY, Tsai YF. Symptom cluster among hemodialysis patients in Taiwan. Appl Nurs Res. 2012;25(3):190-6.

438. Yuan HH, Yang YJ, Zhang YJ, Xue GF, Chen L. The health-related quality of life among patients on maintenance haemodialysis: Evaluation using the "EQ-5D". J Clin Nurs. 2019;28(21-22):4004-11.

439. Zhang AH, Cheng LT, Zhu N, Sun LH, Wang T. Comparison of quality of life and causes of hospitalization between hemodialysis and peritoneal dialysis patients in China. Health Qual Life Outcomes. 2007;5:49.

440. Zhang JC, El-Majzoub S, Li M, Ahmed T, Wu J, Lipman ML, et al. Could symptom burden predict subsequent healthcare use in patients with end stage kidney disease on hemodialysis care? A prospective, preliminary study. Ren Fail. 2020;42(1):294-301.

441. Zhang R, Jia J, Zhang D, Zhao X. Association between fatigue and depressive symptoms among kidney transplantation recipients: The mediating role of rumination. J Adv Nurs. 2019;75(12):3602-8.

442. Zhang W, Shi B, Shi H, Wang J, Wang W, Guan Z, et al. Factors Influencing Health-Related Quality of Life of Living-Donor Kidney Transplant Recipients: A Population-Based Study. Exp Clin Transplant. 2017;15(3):260-6.

443. Zhao L, Yan J, Yang GL, Liu Y. A Study on Adherence to Follow-up, Quality of Life, and Associated Factors Among Renal Transplant Recipients in China. Transplant Proc. 2017;49(6):1285-90.

444. Zhao SM, Dong FF, Qiu HZ, Li D. Quality of Life, Adherence Behavior, and Social Support Among Renal Transplant Recipients in China: A Descriptive Correlational Study. Transplant Proc. 2018;50(10):3329-37.

445. Zheng XY, Han S, Wang LM, Zhu YH, Zeng L, Zhou MS. Quality of life and psychology after living-related kidney transplantation from donors and recipients in China. Transplant Proc. 2014;46(10):3426-30.

446. Zhou W, Hu W, Han G, Wang H, Zhang J, Mei C. The impact of residual renal function on quality of life in patients with peritoneal dialysis. Clin Nephrol. 2018;90(2):106-11.

447. Zimbudzi E, Samlero R, Kerr PG, Zoungas S. How much is enough? An investigation of the relationship between haemodialysis adequacy and quality of life of elderly patients. Nephrology (Carlton). 2016;21(4):314-20.

448. Zyga S, Alikari V, Sachlas A, Stathoulis J, Aroni A, Theofilou P, et al. Management of Pain and Quality of Life in Patients with Chronic Kidney Disease Undergoing Hemodialysis. Pain Manag Nurs. 2015;16(5):712-20.

449. Zyoud SH, Daraghmeh DN, Mezyed DO, Khdeir RL, Sawafta MN, Ayaseh NA, et al. Factors affecting quality of life in patients on haemodialysis: a cross-sectional study from Palestine. BMC Nephrol. 2016;17(1):44.
