# Supplementary material for: Symptom burden and health-related quality of life in chronic kidney disease: A global systematic review and meta-analysis
Source: PLoS Med. 2022 Apr 6;19(4):e1003954. doi: 10.1371/journal.pmed.1003954 (PMC8985967; doi:10.1371/journal.pmed.1003954)
Supplement: S5 Appendix — (DOCX) [file pmed.1003954.s005.docx]

S5 Appendix – Studies excluded after full text screening

[1-182]

1. Quality of Life as Described by Peritoneal Dialysis Patients in Alaska. Nephrology Nursing Journal. 2012;39(2):170-. PubMed PMID: 104557307. Language: English. Entry Date: 20120507. Revision Date: 20150711. Publication Type: Journal Article.

2. Abu Farsakh NA, Rababaa M, Abu Farsakh H. Symptomatic, endoscopic and histological assessment of upper gastrointestinal tract in renal transplant recipients. Indian J Gastroenterol. 2001;20(1):9-12. Epub 2001/02/24. PubMed PMID: 11206884.

3. Akyuz Ozdemir A, Sayin CB, Erdal R, Ozcan C, Haberal M. Quality of Life Through Gender Role Perspective in Candidate Renal Transplant Recipients: A Report From Baskent University Using the Short Form 36 Health Survey. Exp Clin Transplant. 2018;16 Suppl 1(Suppl 1):28-34. Epub 2018/03/13. doi: 10.6002/ect.TOND-TDTD2017.O4. PubMed PMID: 29527987.

4. Alam MB, Khatoon F, Begum SA, Alam MM, Faraji AH, Mahmud MA, et al. Assessment of Quality of Life of Chronic Kidney Disease Patients Receiving Hemodialysis with Kidney Disease Quality of Life-36 Scale. Mymensingh Med J. 2019;28(4):906-13. Epub 2019/10/11. PubMed PMID: 31599259.

5. Alhajim SA. Assessment of the quality of life in patients on haemodialysis in Iraq. Eastern Mediterranean Health Journal. 2017;23(12):815-20. doi: 10.26719/2017.23.12.815. PubMed PMID: WOS:000427439300005.

6. Almutary H, Douglas C, Bonner A. Multidimensional symptom clusters: an exploratory factor analysis in advanced chronic kidney disease. J Adv Nurs. 2016;72(10):2389-400. Epub 2016/05/21. doi: 10.1111/jan.12997. PubMed PMID: 27198911.

7. Almutary H, Douglas C, Bonner A. Towards a symptom cluster model in chronic kidney disease: A structural equation approach. J Adv Nurs. 2017;73(10):2450-61. Epub 2017/03/23. doi: 10.1111/jan.13303. PubMed PMID: 28329420.

8. Alvarez-Rangel LE, Cruz-Santiago J, Meza-Jimenez G, Bernaldez-Gomez G, Ledesma-Gonzalez VM, Camacho-Hernandez F, et al. [Modification of health-related quality of life in kidney transplant recipients]. Rev Med Inst Mex Seguro Soc. 2015;53 Suppl 1:S66-73. Epub 2015/05/29. PubMed PMID: 26020668.

9. Amro A, Waldum-Grevbo B, von der Lippe N, Brekke FB, Miaskowski C, Os I. Symptom Clusters From Dialysis to Renal Transplantation: A Five-Year Longitudinal Study. J Pain Symptom Manage. 2016;51(3):512-9. Epub 2015/11/10. doi: 10.1016/j.jpainsymman.2015.10.012. PubMed PMID: 26550937.

10. Arogundade FA, Abd-Essamie MA, Barsoum RS. Health-related quality of life in emotionally related kidney transplantation: deductions from a comparative study. Saudi J Kidney Dis Transpl. 2005;16(3):311-20. Epub 2007/07/24. PubMed PMID: 17642798.

11. Ay N, Anil M, Alp V, Sevuk U, Dinc B, Celik M, et al. Evaluation of Quality of Life Early and Late After Kidney Transplantation. Ann Transplant. 2015;20:493-9. Epub 2015/08/28. doi: 10.12659/AOT.895139. PubMed PMID: 26313218.

12. Baguelin-Pinaud A, Moinier D, Fouldrin G, Le Roy F, Etienne I, Godin M, et al. [Renal transplantation, anxiety and depressive disorders and quality of life]. Encephale. 2009;35(5):429-35. Epub 2009/10/27. doi: 10.1016/j.encep.2008.06.011. PubMed PMID: 19853715.

13. Boulware LE. Silent kidney disease: many doctors don't recognize the telltale symptoms. Bottom Line Health. 2006;20(12):11-2. PubMed PMID: 106208705. Language: English. Entry Date: 20070112. Revision Date: 20150711. Publication Type: Journal Article. Journal Subset: Consumer Health.

14. Brodin E, Ljungman S, Hedberg M, Sunnerhagen KS. Physical activity, muscle performance and quality of life in patients treated with chronic peritoneal dialysis. Scand J Urol Nephrol. 2001;35(1):71-8. Epub 2001/04/09. doi: 10.1080/00365590151030886. PubMed PMID: 11291692.

15. Brown E. Peritoneal dialysis: older patients report better quality of life than younger. Evid Based Nurs. 2015;18(3):93. Epub 2015/01/22. doi: 10.1136/eb-2014-101989. PubMed PMID: 25605820.

16. Brown EA. What can we do to improve quality of life for the elderly chronic kidney disease patient? Aging Health. 2012;8(5):519-24. PubMed PMID: 104155597. Language: English. Entry Date: 20131106. Revision Date: 20150711. Publication Type: Journal Article.

17. Buzgova R, Smotkova S. [Comparing quality of life in dialysis patients and patients after kidney transplantation: a questionnaire survey]. Cas Lek Cesk. 2013;152(5):233-9. Epub 2013/10/18. PubMed PMID: 24131461.

18. Calia R, Lai C, Aceto P, Luciani M, Camardese G, Lai S, et al. Attachment style predict compliance, quality of life and renal function in adult patients after kidney transplant: preliminary results. Ren Fail. 2015;37(4):678-80. Epub 2015/02/18. doi: 10.3109/0886022X.2015.1010989. PubMed PMID: 25687387.

19. Calia R, Lai C, Aceto P, Luciani M, Camardese G, Lai S, et al. Emotional self-efficacy and alexithymia may affect compliance, renal function and quality of life in kidney transplant recipients: Results from a preliminary cross-sectional study. Physiology & Behavior. 2015;142:152-4. doi: 10.1016/j.physbeh.2015.02.018. PubMed PMID: WOS:000352044000023.

20. Caplin B, Kumar S, Davenport A. Patients' perspective of haemodialysis-associated symptoms. Nephrol Dial Transplant. 2011;26(8):2656-63. Epub 2011/01/08. doi: 10.1093/ndt/gfq763. PubMed PMID: 21212166.

21. Chan R, Brooks R, Erlich J, Chow J, Suranyi M. The effects of kidney-disease-related loss on long-term dialysis patients' depression and quality of life: positive affect as a mediator. Clin J Am Soc Nephrol. 2009;4(1):160-7. Epub 2008/11/07. doi: 10.2215/CJN.01520308. PubMed PMID: 18987298; PubMed Central PMCID: PMCPMC2615692.

22. Chen YC, Hung KY, Kao TW, Tsai TJ, Chen WY. Relationship between dialysis adequacy and quality of life in long-term peritoneal dialysis patients. Perit Dial Int. 2000;20(5):534-40. Epub 2000/12/16. PubMed PMID: 11117244.

23. Cheung KL, Stefanick ML, Allison MA, LeBlanc ES, Vitolins MZ, Shara N, et al. Menopausal symptoms in women with chronic kidney disease. Menopause. 2015;22(9):1006-11. Epub 2015/01/30. doi: 10.1097/GME.0000000000000416. PubMed PMID: 25628057; PubMed Central PMCID: PMCPMC4515400.

24. Chiloff CLM, Cerqueira A, Balbi AL. Quality of life in the treatment of chronic kidney disease: a challenge. J Bras Nefrol. 2017;39(4):351-2. Epub 2018/01/11. doi: 10.5935/0101-2800.20170063. PubMed PMID: 29319757.

25. Chino-Hernandez BE, Acuna-Arellano A, Hernandez-Colin FC, Utrera-Ruiz D, Duarte-Mote J, Garduno-Garcia Jde J. [Quality of life in elderly with chronic kidney disease stage V]. Rev Esp Geriatr Gerontol. 2016;51(5):298-9. Epub 2016/02/26. doi: 10.1016/j.regg.2015.12.011. PubMed PMID: 26908072.

26. Chiu SF, Wong HS, Morad Z, Loo LH. Quality of life in cadaver and living-related renal transplant recipients in Kuala Lumpur hospital. Transplant Proc. 2004;36(7):2030-1. Epub 2004/11/03. doi: 10.1016/j.transproceed.2004.08.105. PubMed PMID: 15518734.

27. Chong VH. Impact of duration of hemodialysis on gastrointestinal symptoms in patients with end stage renal failure. J Gastrointestin Liver Dis. 2010;19(4):462-3. Epub 2010/12/29. PubMed PMID: 21188344.

28. Contreras F, Esguerra GA, Espinosa JC, Gomez V. Coping styles and quality of life in patients with chronic kidney disease (CKD) in treatment with haemodialysis. Acta Colombiana de Psicologia. 10(2):169-79. PubMed PMID: 2008-09214-015.

29. Contreras F, Espinosa JC, Esguerra GA. Quality of life, self-efficacy, coping styles and adherence to treatment in patients with chronic kidney disease undergoing haemodialysis treatment. Psicologia y Salud. 18(2):165-79. PubMed PMID: 2012-09931-003.

30. Corruble E, Barry C, Varescon I, Durrbach A, Samuel D, Lang P, et al. Report of depressive symptoms on waiting list and mortality after liver and kidney transplantation: a prospective cohort study. BMC Psychiatry. 2011;11:182. Epub 2011/11/23. doi: 10.1186/1471-244X-11-182. PubMed PMID: 22103911; PubMed Central PMCID: PMCPMC3231871.

31. Costa-Requena G, Cantarell Aixendri MC, Rodriguez Urrutia A, Seron Micas D. [Health related quality of life and kidney transplantation: a comparison with population values at 6 months post-transplant]. Med Clin (Barc). 2014;142(9):393-6. Epub 2014/02/01. doi: 10.1016/j.medcli.2013.09.044. PubMed PMID: 24480289.

32. Costa-Requena G, Cantarell MC, Moreso F, Parramon G, Seron D. Health related quality of life in renal transplantation: 2 years of longitudinal follow-up. Med Clin (Barc). 2017;149(3):114-8. Epub 2017/04/22. doi: 10.1016/j.medcli.2017.02.032. PubMed PMID: 28427723.

33. Cotera FAU, Alvarez PR. Psychological disturbances and deterioration of health-related quality of life of patients with stage 3-5 chronic kidney disease (not on dialysis). Nefrologia. 2008;28:57-62. PubMed PMID: WOS:000258220200011.

34. Covic A, Jackson J, Hadfield A, Pike J, Siriopol D. Real-World Impact of Cardiovascular Disease and Anemia on Quality of Life and Productivity in Patients with Non-Dialysis-Dependent Chronic Kidney Disease. Adv Ther. 2017;34(7):1662-72. Epub 2017/06/05. doi: 10.1007/s12325-017-0566-z. PubMed PMID: 28578500; PubMed Central PMCID: PMCPMC5504206.

35. de Abreu MM, Walker DR, Sesso RC, Ferraz MB. Health-related quality of life of patients recieving hemodialysis and peritoneal dialysis in Sao Paulo, Brazil: a longitudinal study. Value Health. 2011;14(5 Suppl 1):S119-21. Epub 2011/08/24. doi: 10.1016/j.jval.2011.05.016. PubMed PMID: 21839882.

36. de Alencar SBV, Dias LdA, Dias VdA, de Lima FM, Montarroyos UR, de Petribú KCL. Quality of life may be a more valuable prognostic factor than depression in older hemodialysis patients. Quality of Life Research. 2020;29(7):1829-38. doi: 10.1007/s11136-020-02445-1. PubMed PMID: 143783894. Language: English. Entry Date: In Process. Revision Date: 20210701. Publication Type: journal article. Journal Subset: Allied Health.

37. de Farias GM, de Mendonça AEO. Comparing quality of life of patients in hemodialisys and post-renal transplant using the 'WHOQOL-bref'. Revista Mineira de Enfermagem. 2009;13(4):574-83. PubMed PMID: 105194863. Language: Portuguese. Entry Date: 20100702. Revision Date: 20150818. Publication Type: Journal Article.

38. de Oliveira MP, Kusumota L, Haas V, Jose e, de Cassia Helu Mendonca Ribeiro R, Marques S, et al. Health-related quality of life as a predictor of mortality in patients on peritoneal dialysis. Revista Latino-Americana de Enfermagem Vol 24 2016, ArtID e2794. 2016;24. PubMed PMID: 2016-49805-001.

39. de Ortuzar MG. Ethics and quality of life of kidney transplant patient. Transplant Proc. 2001;33(1-2):1913-6. Epub 2001/03/27. doi: 10.1016/s0041-1345(00)02712-3. PubMed PMID: 11267568.

40. De Pasquale C, Pistorio ML, Veroux P, Giuffrida G, Sinagra N, Ekser B, et al. Quality of life in kidney transplantation from marginal donors. Transplant Proc. 2011;43(4):1045-7. Epub 2011/05/31. doi: 10.1016/j.transproceed.2011.01.156. PubMed PMID: 21620049.

41. De Pasquale C, Veroux M, Pistorio ML, Papotto A, Basile G, Patane M, et al. Return to Work and Quality of Life: A Psychosocial Survey After Kidney Transplant. Transplant Proc. 2019;51(1):153-6. Epub 2019/01/19. doi: 10.1016/j.transproceed.2018.04.083. PubMed PMID: 30655159.

42. Decker O, Overbeck I, Mohs A, Bartels M, Geisse B, Hauss J, et al. Comparison of quality of life of dialysis patients on the waiting list and patients after kidney transplantation. Zeitschrift fur Medizinische Psychologie. 2008;17(1):27-30. PubMed PMID: 2008-02219-005.

43. Delgado CE, Jaramillo MM, Orozco BE, Santaella MH, Nunez JJ, Munoz JP, et al. [Quality of life in patients with chronic kidney disease without dialysis or transplant: a random sample from two insurance companies. Medellin, Colombia, 2008]. Nefrologia. 2009;29(6):548-56. Epub 2009/11/26. doi: 10.3265/Nefrologia.2009.29.6.5490.en.full. PubMed PMID: 19935999.

44. Dogan U, Yaprak M, Dogan EA, Onac M, Yilmaz VT, Aydinli B. Cardiovascular and Neurologic Complications in Kidney Transplant Recipients: A Focused Appraisal of Symptoms. Transplant Proc. 2019;51(4):1101-7. Epub 2019/05/19. doi: 10.1016/j.transproceed.2019.02.005. PubMed PMID: 31101180.

45. Egea-Guerrero JJ, Martin-Villen L, Ruiz de Azua-Lopez Z. [Quality of life after kidney transplant]. Med Clin (Barc). 2016;147(7):326. Epub 2016/07/20. doi: 10.1016/j.medcli.2016.05.033. PubMed PMID: 27431889.

46. El Kass SdMA, El-Senousy TA, Jumaa NA. Factors Affecting Quality of Life among Patients Undergoing Hemodialysis Program in Gaza Strip. International Journal of Caring Sciences. 2020;13(2):1221-9. PubMed PMID: 146255985. Language: English. Entry Date: 20201016. Revision Date: 20201016. Publication Type: Article.

47. Elder SJ, Pisoni RL, Akizawa T, Fissell R, Andreucci VE, Fukuhara S, et al. Sleep quality predicts quality of life and mortality risk in haemodialysis patients: results from the Dialysis Outcomes and Practice Patterns Study (DOPPS). Nephrol Dial Transplant. 2008;23(3):998-1004. Epub 2007/10/04. doi: 10.1093/ndt/gfm630. PubMed PMID: 17911092.

48. Ellis P. CKD associated with poorer quality of life. Journal of Renal Nursing. 2011;3(3):146-7. PubMed PMID: 104668139. Language: English. Entry Date: 20110802. Revision Date: 20150711. Publication Type: Journal Article.

49. Ergesi B, Winkler Y, Kistler T, Grimm MO, John H, Horstmann M. Prevalence and Management of Lower Urinary Tract Symptoms Related to Benign Prostatic Obstruction in a Contemporary Series of Renal Transplant Recipients. Nephrourol Mon. 2016;8(2):e35497. Epub 2016/05/28. doi: 10.5812/numonthly.35497. PubMed PMID: 27231686; PubMed Central PMCID: PMCPMC4879790.

50. Farag YM, Keithi-Reddy SR, Mittal BV, Surana SP, Addabbo F, Goligorsky MS, et al. Anemia, inflammation and health-related quality of life in chronic kidney disease patients. Clin Nephrol. 2011;75(6):524-33. Epub 2011/05/27. doi: 10.5414/cnp75524. PubMed PMID: 21612756.

51. Fayyazi S, Athari SH, Asadizaker M, Rasekh A. Comparative evaluation of QOL in haemodialysis and kidney transplantation patients. CONNECT: The World of Critical Care Nursing. 2005;4(3):91-2. PubMed PMID: 106343860. Language: English. Entry Date: 20061006. Revision Date: 20150711. Publication Type: Journal Article.

52. Ferradini MA, Pogliani D. [Quality of life after kidney transplant]. G Ital Nefrol. 2008;25(5):581-5. Epub 2008/10/02. PubMed PMID: 18828121.

53. Finkelstein AF, Wuerth D, Finkelstein SH. Quality of life assessments in hemodialysis and peritoneal dialysis patients: an important dimension of patient choice why is the evidence favoring hemodialysis over peritoneal dialysis misleading? Semin Dial. 2007;20(3):211-3. Epub 2007/06/09. doi: 10.1111/j.1525-139X.2007.00278.x. PubMed PMID: 17555485.

54. Finkelstein FO, Story K, Firanek C, Mendelssohn D, Barre P, Takano T, et al. Health-related quality of life and hemoglobin levels in chronic kidney disease patients. Clin J Am Soc Nephrol. 2009;4(1):33-8. Epub 2008/11/07. doi: 10.2215/CJN.00630208. PubMed PMID: 18987300; PubMed Central PMCID: PMCPMC2615698.

55. Ford S. The 13 most common kidney disease symptoms have been distilled by researchers. Nursing Times. 2018;114(9):46-. PubMed PMID: 132450526. Language: English. Entry Date: 20181029. Revision Date: 20181029. Publication Type: Article. Journal Subset: Core Nursing.

56. Franke GH, Heemann U, Kohnle M, Luetkes P, Maehner N, Reimer J. Quality of life in patients before and after kidney transplantation. Psychology & Health. 14(6):1037-49. PubMed PMID: 2000-14294-005.

57. Franke GH, Yucetin L, Yaman H, Reimer J, Demirbas A. Disease-specific quality of life in Turkish patients after successful kidney transplantation. Transplant Proc. 2006;38(2):457-9. Epub 2006/03/22. doi: 10.1016/j.transproceed.2005.12.110. PubMed PMID: 16549146.

58. Garcia-Martinez P, Ballester-Arnal R, Gandhi-Morar K, Castro-Calvo J, Gea-Caballero V, Juarez-Vela R, et al. Perceived Stress in Relation to Quality of Life and Resilience in Patients with Advanced Chronic Kidney Disease Undergoing Hemodialysis. International Journal of Environmental Research & Public Health [Electronic Resource]. 2021;18(2):11. doi: <https://dx.doi.org/10.3390/ijerph18020536>. PubMed PMID: 33440671.

59. Garth K. Time-related centile ranges for quality of life outcomes in renal transplantation: University of Tennessee Health Science Center; 2008.

60. Gemmell LA, Terhorst L, Jhamb M, Unruh M, Myaskovsky L, Kester L, et al. Gender and Racial Differences in Stress, Coping, and Health-Related Quality of Life in Chronic Kidney Disease. J Pain Symptom Manage. 2016;52(6):806-12. Epub 2016/10/05. doi: 10.1016/j.jpainsymman.2016.05.029. PubMed PMID: 27697565; PubMed Central PMCID: PMCPMC5156935.

61. Gil Cunqueiro JM, Garcia Cortes MJ, Foronda J, Borrego JF, Sanchez Perales MC, Perez del Barrio P, et al. [Health-related quality of life in elderly patients in haemodialysis]. Nefrologia. 2003;23(6):528-37. Epub 2004/03/09. PubMed PMID: 15002788.

62. Granados NM, Ortiz RV, Reyes HM. Chronic kidney disease associated pruritus impact on dermatological quality of life of patients with chronic renal replacement therapy in hemodialysis from the General Hospital of Mexico. Journal of the American Academy of Dermatology. 2016;74(5):Ab168-Ab. PubMed PMID: WOS:000412760201546.

63. Grincenkov FR, Fernandes N, Pereira Bdos S, Bastos K, Lopes AA, Finkelstein FO, et al. Impact of baseline health-related quality of life scores on survival of incident patients on peritoneal dialysis: a cohort study. Nephron. 2015;129(2):97-103. Epub 2015/01/31. doi: 10.1159/000369139. PubMed PMID: 25633060.

64. Grove BE, Schougaard LM, Hjollund NH, Ivarsen P. Self-rated health, quality of life and appetite as predictors of initiation of dialysis and mortality in patients with chronic kidney disease stages 4-5: a prospective cohort study. BMC Res Notes. 2018;11(1):371. Epub 2018/06/10. doi: 10.1186/s13104-018-3472-9. PubMed PMID: 29884242; PubMed Central PMCID: PMCPMC5994035.

65. Guruprasad P, Kishore K, Mahajan S, Aggarwal S. Active surveillance for adverse events among patients who underwent renal transplantation: A prospective observational study. Perspect Clin Res. 2017;8(3):118-23. Epub 2017/08/23. doi: 10.4103/2229-3485.210447. PubMed PMID: 28828306; PubMed Central PMCID: PMCPMC5543762.

66. Gutierrez Sanchez D, Leiva-Santos JP, Macias Lopez MJ, Cuesta-Vargas AI. Prevalence of symptoms in advanced chronic kidney disease. Nefrologia. 2018;38(5):560-2. Epub 2018/10/15. doi: 10.1016/j.nefro.2017.11.021. PubMed PMID: 30316480.

67. Gutiérrez-Peredo GB, Martins MTS, da Silva FA, Lopes MB, Lopes GB, Lopes AA. Functional dependence and the mental dimension of quality of life in Hemodialysis patients: the PROHEMO study. Health & Quality of Life Outcomes. 2020;18(1):1-10. doi: 10.1186/s12955-020-01464-3. PubMed PMID: 144656168. Language: English. Entry Date: In Process. Revision Date: 20201015. Publication Type: journal article.

68. Harhay MN, Yang W, Sha D, Roy J, Chai B, Fischer MJ, et al. Health-Related Quality of Life, Depressive Symptoms, and Kidney Transplant Access in Advanced CKD: Findings From the Chronic Renal Insufficiency Cohort (CRIC) Study. Kidney Medicine. 2020;2(5):600-9.e1. doi: <https://dx.doi.org/10.1016/j.xkme.2020.06.010>. PubMed PMID: 33089138.

69. Humar A, Denny R, Matas AJ, Najarian JS. Graft and quality of life outcomes in older recipients of a kidney transplant. Exp Clin Transplant. 2003;1(2):69-72. Epub 2005/04/30. PubMed PMID: 15859911.

70. Ibrahim N, Teo SS, Che Din N, Abdul Gafor AH, Ismail R. The Role of Personality and Social Support in Health-Related Quality of Life in Chronic Kidney Disease Patients. PLoS One. 2015;10(7):e0129015. Epub 2015/07/02. doi: 10.1371/journal.pone.0129015. PubMed PMID: 26131714; PubMed Central PMCID: PMCPMC4488553.

71. Ibrahimou B, Albatineh AN. Predictors of Quality of Life among Peritoneal Dialysis Patients with End-Stage Renal Disease in Kuwait. Perit Dial Int. 2019;39(2):180-2. Epub 2019/03/13. doi: 10.3747/pdi.2018.00140. PubMed PMID: 30858286.

72. James G, Nyman E, Fitz-Randolph M, Niklasson A, Hedman K, Hedberg J, et al. Characteristics, Symptom Severity, and Experiences of Patients Reporting Chronic Kidney Disease in the PatientsLikeMe Online Health Community: Retrospective and Qualitative Study. Journal of Medical Internet Research. 2020;22(7):e18548. doi: <https://dx.doi.org/10.2196/18548>. PubMed PMID: 32673242.

73. Jamilano K, Fowler J, Richards M, Cooper CW, Richards N. A quality of life assessment tool for haemodialysis patients in Abu Dhabi. Journal of Kidney Care. 2019;4(3):140-6. PubMed PMID: rayyan-52379014.

74. Janmaat CJ, van Diepen M, Meuleman Y, Chesnaye NC, Drechsler C, Torino C, et al. Kidney function and symptom development over time in elderly patients with advanced chronic kidney disease: results of the EQUAL cohort study. Nephrology Dialysis Transplantation. 2021;36(5):862-70. doi: <https://dx.doi.org/10.1093/ndt/gfz277>. PubMed PMID: 31943084.

75. John MM, Gupta A, Sharma RK, Kaul A. Impact of residual renal function on clinical outcome and quality of life in patients on peritoneal dialysis. Saudi J Kidney Dis Transpl. 2017;28(1):30-5. Epub 2017/01/18. doi: 10.4103/1319-2442.198109. PubMed PMID: 28098100.

76. Joshi SA, Almeida N, Almeida A. Assessment of the perceived quality of life of successful kidney transplant recipients and their donors pre- and post-transplantation. Transplant Proc. 2013;45(4):1435-7. Epub 2013/06/04. doi: 10.1016/j.transproceed.2013.01.037. PubMed PMID: 23726590.

77. Juergensen PH, Zemchenkov A, Watnick S, Finkelstein S, Wuerth D, Finkelstein FO. Comparison of quality-of-life assessment in Russia and the United States in chronic peritoneal dialysis patients. Adv Perit Dial. 2002;18:55-7. Epub 2002/10/31. PubMed PMID: 12402587.

78. Junchotikul P, Charoenthanakit C, Saiyud A, Parapiboon W, Ingsathit A, Jirasiritham S, et al. Assessment of the Changes in Health-related Quality of Life After Kidney Transplantation in a Cohort of 232 Thai Patients. Transplant Proc. 2015;47(6):1732-5. Epub 2015/08/22. doi: 10.1016/j.transproceed.2015.02.018. PubMed PMID: 26293042.

79. Jvanbakhtian R, Hosseini RS. A Study of Physical and Psychological Domains of Quality of Life in Kidney Transplantation Patients in Kerman City, Iran. Qom University of Medical Sciences Journal. 2013;7(5):41-2. PubMed PMID: 104030129. Language: English. Entry Date: 20140221. Revision Date: 20150710. Publication Type: Journal Article.

80. Kalfoss M, Schick-Makaroff K, Molzahn AE. Living with Chronic Kidney Disease: Illness Perceptions, Symptoms, Coping, and Quality of Life. Nephrol Nurs J. 2019;46(3):277-90. Epub 2019/06/15. PubMed PMID: 31199095.

81. Kallay E, Pop R, Balazsi R. Distress, emotional profile, and quality of life in Romanian chronic renal failure and renal transplant patients. Psychology & Health. 2010;25(3):47-. PubMed PMID: WOS:000325913600087.

82. Kamran F, Fife-Schaw C. Do clinical factors influence quality of life among renal transplant patients. Journal of Behavioural Sciences. 2014;24(1):1-20. PubMed PMID: 2014-24024-001.

83. Kamran F, Fife-Schaw C. Socio demographic correlates of quality of life in renal transplant recipients in Pakistan: A longitudinal study. Journal of Behavioural Sciences. 2014;24(2):37-51. PubMed PMID: 2015-01123-003.

84. Karaca S, Çınar S, Bahçebaşı ZB. The perspective from the patients. The effects of peritoneal dialysis on life and mental symptoms. Journal of Marmara University Institute of Health Sciences. 2012;2(4):169-74. PubMed PMID: 104405632. Language: Turkish. Entry Date: 20130107. Revision Date: 20150711. Publication Type: Journal Article.

85. Khedmat H, Karami GR, Pourfarziani V, Assari S, Rezailashkajani M, Naghizadeh MM. A logistic regression model for predicting health-related quality of life in kidney transplant recipients. Transplant Proc. 2007;39(4):917-22. Epub 2007/05/26. doi: 10.1016/j.transproceed.2007.04.004. PubMed PMID: 17524850.

86. Kim HS, So HS. [A prediction model development on quality of life in kidney transplant recipients]. J Korean Acad Nurs. 2009;39(4):518-27. Epub 2009/09/04. doi: 10.4040/jkan.2009.39.4.518. PubMed PMID: 19726908.

87. Kim HW, Choi-Kwon S. [Structural equation modeling on quality of life in pre-dialysis patients with chronic kidney disease]. J Korean Acad Nurs. 2012;42(5):699-708. Epub 2012/12/12. doi: 10.4040/jkan.2012.42.5.699. PubMed PMID: 23221659.

88. Kim SJ, Song SH, Kim JH, Kwak IS. Statistical parametric mapping analysis of the relationship between regional cerebral blood flow and symptom clusters of the depressive mood in patients with pre-dialytic chronic kidney disease. Ann Nucl Med. 2008;22(3):201-6. Epub 2008/05/24. doi: 10.1007/s12149-007-0108-x. PubMed PMID: 18498035.

89. Kizilisik AT, Feurer ID, VanBuren DH, Wise P, Van Buren D, Hopkins J, et al. Effects of diabetes and cadaveric organs on functional performance and health-related quality of life after kidney transplantation. Am J Surg. 2003;186(5):535-9. Epub 2003/11/06. doi: 10.1016/j.amjsurg.2003.07.013. PubMed PMID: 14599621.

90. Kong JH, Davies MRP, Mount PF. Relationship between residual kidney function and symptom burden in haemodialysis patients. Internal Medicine Journal. 2021;51(1):52-61. doi: <https://dx.doi.org/10.1111/imj.14775>. PubMed PMID: 32043691.

91. Kusleikaite N, Bumblyte IA, Pakalnyte R. [Quality of life and depression in renal transplant patients]. Medicina (Kaunas). 2007;43 Suppl 1:103-8. Epub 2007/12/06. PubMed PMID: 17551286.

92. Kutner NG, Zhang R, Barnhart H, Collins AJ. Health status and quality of life reported by incident patients after 1 year on haemodialysis or peritoneal dialysis. Nephrol Dial Transplant. 2005;20(10):2159-67. Epub 2005/07/28. doi: 10.1093/ndt/gfh973. PubMed PMID: 16046520.

93. Kutner NG, Zhang R, Huang Y, Bliwise DL. Association of sleep difficulty with Kidney Disease Quality of Life cognitive function score reported by patients who recently started dialysis. Clin J Am Soc Nephrol. 2007;2(2):284-9. Epub 2007/08/21. doi: 10.2215/CJN.03000906. PubMed PMID: 17699426.

94. Lai S, Amabile MI, Bargagli MB, Musto TG, Martinez A, Testorio M, et al. Peritoneal dialysis in older adults: Evaluation of clinical, nutritional, metabolic outcomes, and quality of life. Medicine (Baltimore). 2018;97(35):e11953. Epub 2018/09/02. doi: 10.1097/MD.0000000000011953. PubMed PMID: 30170391; PubMed Central PMCID: PMCPMC6393061.

95. Laudanski K, Nowak Z, Niemczyk S. Age-related differences in the quality of life in end-stage renal disease in patients enrolled in hemodialysis or continuous peritoneal dialysis. Med Sci Monit. 2013;19:378-85. Epub 2013/05/21. doi: 10.12659/MSM.883916. PubMed PMID: 23685340; PubMed Central PMCID: PMCPMC3665666.

96. Li C, Mao W, Liu X. Depression, anxiety and reduced quality of life in predialysis: differences across the CKD stages? Int J Clin Pract. 2014;68(5):657-8. Epub 2014/04/23. doi: 10.1111/ijcp.12422. PubMed PMID: 24750530.

97. Lichodziejewska-Niemierko M, Bobel-Olchowik B, Majkowicz M, Afeltowicz Z, Liberek T, Rutkowski B. [Quality of life and therapy in the elderly patients on chronic peritoneal dialysis]. Pol Merkur Lekarski. 2003;15(88):330-1; discussion 1-3. Epub 2004/02/21. PubMed PMID: 14974360.

98. Liu H. Coping and health-related quality of life in renal transplant patients: Vanderbilt University; 2006.

99. Makkar V, Kumar M, Mahajan R, Khaira NS. Comparison of Outcomes and Quality of Life between Hemodialysis and Peritoneal Dialysis Patients in Indian ESRD Population. J Clin Diagn Res. 2015;9(3):OC28-31. Epub 2015/05/09. doi: 10.7860/JCDR/2015/11472.5709. PubMed PMID: 25954652; PubMed Central PMCID: PMCPMC4413101.

100. Manley KJ. Taste genetics and gastrointestinal symptoms experienced in chronic kidney disease. Eur J Clin Nutr. 2015;69(7):781-5. Epub 2015/05/28. doi: 10.1038/ejcn.2015.80. PubMed PMID: 26014268.

101. Maoxin HE, Yujie Y, Shaobin YU. Correlation analysis of quality of life and health literacy in middle-aged and elderly patients undergoing maintenance hemodialysis. Chinese Nursing Research. 2020;(21):3886-9. doi: 10.12102/j.issn.1009-6493.2020.21.027. PubMed PMID: 147008504. Language: Chinese. Entry Date: 20201123. Revision Date: 20201123. Publication Type: Article.

102. Markell MS, Terebelo S. Complementary Medicine Use, Symptom Burden and Non-Adherence in Kidney Transplant Recipients. Explore (NY). 2018;14(6):414-9. Epub 2018/10/20. doi: 10.1016/j.explore.2018.04.010. PubMed PMID: 30337196.

103. Martinez-Sanchis S, Bernal MC, Montagud JV, Abad A, Crespo J, Pallardo LM. Quality of life and stressors in patients with chronic kidney disease depending on treatment. Span J Psychol. 2015;18:E25. Epub 2015/04/29. doi: 10.1017/sjp.2015.17. PubMed PMID: 25919086.

104. Matsumura S, Unagami K, Okabe S, Fukuda H, Suzuki A, Fuchinoue S, et al. Comparative Study on Variation of Quality of Life of Patients of Preemptive Kidney Transplantation and Nonpreemptive Kidney Transplantation. Transplant Proc. 2018;50(10):3321-8. Epub 2018/12/24. doi: 10.1016/j.transproceed.2018.08.024. PubMed PMID: 30577202.

105. Mazairac AH, de Wit GA, Penne EL, van der Weerd NC, Grooteman MP, van den Dorpel MA, et al. Protein-energy nutritional status and kidney disease-specific quality of life in hemodialysis patients. J Ren Nutr. 2011;21(5):376-86 e1. Epub 2011/01/05. doi: 10.1053/j.jrn.2010.08.004. PubMed PMID: 21194971.

106. Megawati, Yetti K, Sukmarini L. The factors affecting the quality of life of kidney transplantation patients at the Cipto Mangunkusumo General Hospital in Jakarta, Indonesia. Enferm Clin. 2019;29 Suppl 2:428-33. Epub 2019/07/13. doi: 10.1016/j.enfcli.2019.04.063. PubMed PMID: 31296463.

107. Michels WM, van Dijk S, Verduijn M, le Cessie S, Boeschoten EW, Dekker FW, et al. Quality of life in automated and continuous ambulatory peritoneal dialysis. Perit Dial Int. 2011;31(2):138-47. Epub 2011/03/02. doi: 10.3747/pdi.2010.00063. PubMed PMID: 21357936.

108. Milovanov YS, Dobrosmyslov IA, Milovanova SY, Taranova MV, Milovanova LY, Fomin VV, et al. Quality of life of chronic kidney disease patients on renal replacement therapy. Ter Arkh. 2018;90(6):89-91. Epub 2019/02/01. doi: 10.26442/terarkh201890689-91. PubMed PMID: 30701910.

109. Mitra S, ip, Jayanti A. Clinical outcomes and quality of life for home haemodialysis patients. Journal of Renal Nursing. 2014;6(5):220-5. PubMed PMID: 107831004. Language: English. Entry Date: 20141003. Revision Date: 20150819. Publication Type: Journal Article.

110. Mor Fukushima RL, Costa Menezes AL, Inouye K, Iost Pavarini SC, Orl dS, i F. Quality of life and associated factors in patients with chronic kidney disease on hemodialysis. Acta Paulista de Enfermagem. 2016;29(5):518-24. PubMed PMID: 120942278. Language: English. Entry Date: 20170203. Revision Date: 20170203. Publication Type: Article.

111. Mouelhi Y, Jouve E, Alessandrini M, Pedinielli N, Moal V, Meurette A, et al. Factors associated with Health-Related Quality of Life in Kidney Transplant Recipients in France. BMC Nephrol. 2018;19(1):99. Epub 2018/04/29. doi: 10.1186/s12882-018-0893-6. PubMed PMID: 29703170; PubMed Central PMCID: PMCPMC5921567.

112. Nagarathnam M, Sivakumar V, Latheef SAA. Burden, coping mechanisms, and quality of life among caregivers of hemodialysis and peritoneal dialysis undergoing and renal transplant patients. Indian Journal of Psychiatry. 2019;61(4):380-8. doi: 10.4103/psychiatry.IndianJPsychiatry_401_18. PubMed PMID: WOS:000475770700010.

113. Nasr M, Hadj Ammar M, Khammouma S, Ben Dhia N, Ghachem A. [Haemodialysis and its impact on the quality of life]. Nephrol Ther. 2008;4(1):21-7. Epub 2007/12/29. doi: 10.1016/j.nephro.2007.07.008. PubMed PMID: 18162450.

114. Nguyen NTQ, Cockwell P, Maxwell AP, Griffin M, O'Brien T, O'Neill C. Chronic kidney disease, health-related quality of life and their associated economic burden among a nationally representative sample of community dwelling adults in England. PLoS One. 2018;13(11):e0207960. Epub 2018/11/27. doi: 10.1371/journal.pone.0207960. PubMed PMID: 30475893; PubMed Central PMCID: PMCPMC6258125.

115. Niemczyk M. Health-related quality of life in kidney transplant recipients. Nephrourol Mon. 2013;5(3):851. Epub 2013/11/28. doi: 10.5812/numonthly.9490. PubMed PMID: 24282801; PubMed Central PMCID: PMCPMC3830917.

116. Oh SH, Yoo EK. [Comparison of quality of life between kidney transplant and hemodialysis patients]. Taehan Kanho Hakhoe Chi. 2006;36(7):1145-53. Epub 2007/01/11. doi: 10.4040/jkan.2006.36.7.1145. PubMed PMID: 17211117.

117. Oh TR, Choi HS, Kim CS, Bae EH, Oh YK, Kim YS, et al. Association between health related quality of life and progression of chronic kidney disease. Sci Rep. 2019;9(1):19595. Epub 2019/12/22. doi: 10.1038/s41598-019-56102-w. PubMed PMID: 31863079; PubMed Central PMCID: PMCPMC6925203.

118. Okaka EI, Davies M, Ahmed M, Naidoo S, Naicker S. Impact of socio-economic factors on quality of life in patients on continuous ambulatory peritoneal dialysis in an African setting. West Afr J Med. 2014;33(2):125-9. Epub 2014/09/23. PubMed PMID: 25236829.

119. Okubo R, Kai H, Kondo M, Saito C, Yoh K, Morito N, et al. Health-related quality of life and prognosis in patients with chronic kidney disease: a 3-year follow-up study. Clin Exp Nephrol. 2014;18(5):697-703. Epub 2013/11/08. doi: 10.1007/s10157-013-0901-x. PubMed PMID: 24198050.

120. Oren B, Enc N. Quality of life in chronic haemodialysis and peritoneal dialysis patients in Turkey and related factors. Int J Nurs Pract. 2013;19(6):547-56. Epub 2013/12/18. doi: 10.1111/ijn.12098. PubMed PMID: 24330205.

121. Ortega Suarez F. [Health related quality of life in the kidney transplant patient]. Med Clin (Barc). 2014;142(9):397-8. Epub 2014/03/04. doi: 10.1016/j.medcli.2014.01.007. PubMed PMID: 24581841.

122. Ortiz F, Aronen P, Koskinen PK, Malmstrom RK, Finne P, Honkanen EO, et al. Health-related quality of life after kidney transplantation: who benefits the most? Transpl Int. 2014;27(11):1143-51. Epub 2014/07/01. doi: 10.1111/tri.12394. PubMed PMID: 24977951.

123. Ostrowski M, Wesolowski T, Makar D, Bohatyrewicz R. Changes in patients' quality of life after renal transplantation. Transplant Proc. 2000;32(6):1371-4. Epub 2000/09/21. doi: 10.1016/s0041-1345(00)01261-6. PubMed PMID: 10995983.

124. Peng YS, Chiang CK, Hung KY, Chang CH, Lin CY, Yang CS, et al. Comparison of self-reported health-related quality of life between Taiwan hemodialysis and peritoneal dialysis patients: a multi-center collaborative study. Quality of Life Research. 2011;20(3):399-405. doi: 10.1007/s11136-010-9755-9. PubMed PMID: WOS:000288214800010.

125. Perales Montilla CM, Duschek S, Reyes Del Paso GA. Quality of life related to health chronic kidney disease: Predictive importance of mood and somatic symptoms. Nefrologia. 2016;36(3):275-82. Epub 2016/04/14. doi: 10.1016/j.nefro.2015.12.002. PubMed PMID: 27068350.

126. Peters L, Franke L, Tkachenko D, Schiffer M, Zimmermann T. [Impact of Emotion Regulation and Emotional Arousal on Quality of Life and Adherence of Couples after Kidney Transplantation]. Psychother Psychosom Med Psychol. 2018;68(5):202-11. Epub 2018/05/04. doi: 10.1055/s-0044-102012. PubMed PMID: 29723908.

127. Pinillos-Patino Y, Herazo-Beltran Y, Gil Catano J, Ramos de Avila J. [Association of physical activity with quality of life in patients with chronic kidney disease]. Rev Med Chil. 2019;147(2):153-60. Epub 2019/05/17. doi: 10.4067/s0034-98872019000200153. PubMed PMID: 31095162.

128. Pollice R, Di Mauro S, Bernardini M, Bianchini V, Giordani Paesani N, Ussorio D, et al. [Psychopathology, quality of life and social functioning in dialysis treatment and kidney transplantation patients]. Clin Ter. 2010;161(4):329-33. Epub 2010/10/12. PubMed PMID: 20931155.

129. Ponton P, Rupolo GP, Marchini F, Feltrin A, Perin N, Mazzoldi MA, et al. Quality-of-life change after kidney transplantation. Transplant Proc. 2001;33(1-2):1887-9. Epub 2001/03/27. doi: 10.1016/s0041-1345(00)02737-8. PubMed PMID: 11267555.

130. Possemato K, Geller PA, Ouimette P. Posttraumatic stress and quality of life in kidney transplantation recipients. Traumatology. 15(3):34-9. PubMed PMID: 2009-17906-005.

131. Powe NR. Health-Related Quality of Life in CKD-Advancing Patient-Centered Research to Transform Patient Care. Clin J Am Soc Nephrol. 2016;11(7):1123-4. Epub 2016/06/02. doi: 10.2215/CJN.04730416. PubMed PMID: 27246011; PubMed Central PMCID: PMCPMC4934834.

132. Procaccini DA, Angelini P, Aucella F, Avanzi C, Brusasco S, Carta G, et al. [Health-related quality of life in patients with chronic kidney disease]. G Ital Nefrol. 2008;25(6):694-701. Epub 2008/12/03. PubMed PMID: 19048570.

133. Pucheu S, Consoli SM, D'Auzac C, Francais P, Issad B. Do health causal attributions and coping strategies act as moderators of quality of life in peritoneal dialysis patients? Journal of Psychosomatic Research. 56(3):317-22. PubMed PMID: 2004-13130-009.

134. Pugh-Clarke K, Naish PF, Mercer TM. [Quality of life in chronic kidney disease]. J Ren Care. 2006;32(3):167-71. Epub 2007/03/31. doi: 10.1111/j.1755-6686.2006.tb00010.x. PubMed PMID: 17393813.

135. Quintal-Medina IA, Rocha-Gonzalez HI, Noyola-Garcia ME, Flores-Padilla MG. Factors associated with residual symptom burden in patients with peritoneal dialysis: a cohort study. Revista Medica del Instituto Mexicano del Seguro Social. 2020;58(2):174-80. doi: <https://dx.doi.org/10.24875/RMIMSS.M20000015>. PubMed PMID: 34101562.

136. Raiisifar A, Tayyebi A, Ebadi A, Najafi S, Hashemi S, Asiyabi M. An investigation of Quality of life in Kidney Transplant Patients. Iranian Journal of Critical Care Nursing. 2011;4(3):149-52. PubMed PMID: 104313226. Language: English. Entry Date: 20130315. Revision Date: 20150711. Publication Type: Journal Article.

137. Raimundo Leone DR, Amorim Pereira G, Cristian de Paula Silva A, Silva de Aguiar A. Level of activation and quality of life related to the health of people in hemodialysis. Anna Nery School Journal of Nursing / Escola Anna Nery Revista de Enfermagem. 2021;25(4):1-12. doi: 10.1590/2177-9465-EAN-2020-0486. PubMed PMID: 149825317. Language: English. Entry Date: 20210423. Revision Date: 20210423. Publication Type: Article.

138. Rasmussen K. Quality of life and the relative superiority of different peritoneal dialysis modalities. Perit Dial Int. 2011;31(2):134. Epub 2011/03/24. doi: 10.3747/pdi.2010.00295. PubMed PMID: 21427245.

139. Ratti MM, Delli Zotti GB, Sangiovanni E, Vai B, Limido A, Bertoli S, et al. [Quality of life, anxiety and distress in patients suffering from chronic kidney disease: pre-dialysis and start of dialytic treatment]. G Ital Nefrol. 2017;34(1):Jan-Feb. Epub 2017/02/09. PubMed PMID: 28177103.

140. Ravagnani LMB, Domingos NAM, de Oliveira Santos Miyazaki MC. Quality of life and coping strategies in patients undergoing renal transplantation. Estudos de Psicologia. 12(2):177-84. PubMed PMID: 2008-01601-010.

141. Raymond J, Johnson ST, Diehl-Jones W, Vallance JK. Walking, Sedentary Time and Health-Related Quality Life Among Kidney Transplant Recipients: An Exploratory Study. Transplant Proc. 2016;48(1):59-64. Epub 2016/02/27. doi: 10.1016/j.transproceed.2015.12.022. PubMed PMID: 26915844.

142. Rebollo P, Arenas MD, Castejon N, Reichert GJ, Delgado CP, Gutierrez RP, et al. Pain Incidence and Analgesic Consumption During Haemodialysis Sessions: Impact on Health-Related Quality Of Life. Value Health. 2014;17(7):A521-2. Epub 2014/11/01. doi: 10.1016/j.jval.2014.08.1631. PubMed PMID: 27201632.

143. Reimer J, Franke GH, Lutkes P, Kohnle M, Gerken G, Philipp T, et al. Quality of life in patients before and after kidney transplantation. PPmP: Psychotherapie Psychosomatik Medizinische Psychologie. 52(1):16-23. PubMed PMID: 2002-00536-001.

144. Ricardo AC, Goh V, Chen J, Cedillo-Couvert E, Kapella M, Prasad B, et al. Association of Sleep Duration, Symptoms, and Disorders with Mortality in Adults with Chronic Kidney Disease. Kidney Int Rep. 2017;2(5):866-73. Epub 2017/10/24. doi: 10.1016/j.ekir.2017.05.002. PubMed PMID: 29057381; PubMed Central PMCID: PMCPMC5648366.

145. Rivara MB, Robinson-Cohen C, Kestenbaum B, Roshanravan B, Chen CH, Himmelfarb J, et al. Changes in symptom burden and physical performance with initiation of dialysis in patients with chronic kidney disease. Hemodial Int. 2015;19(1):147-50. Epub 2014/11/19. doi: 10.1111/hdi.12244. PubMed PMID: 25404416; PubMed Central PMCID: PMCPMC4420177.

146. Rodrigo C, de Silva A, Lanerolle R. Symptoms of anxiety among patients with chronic kidney disease in Sri Lanka. Saudi J Kidney Dis Transpl. 2013;24(6):1256-7. Epub 2013/11/16. doi: 10.4103/1319-2442.121302. PubMed PMID: 24231498.

147. Ronai KZ, Szentkiralyi A, Lazar AS, Ujszaszi A, Turanyi C, Gombos F, et al. Depressive Symptoms Are Associated With Objectively Measured Sleep Parameters in Kidney Transplant Recipients. J Clin Sleep Med. 2017;13(4):557-64. Epub 2017/02/07. doi: 10.5664/jcsm.6542. PubMed PMID: 28162142; PubMed Central PMCID: PMCPMC5359332.

148. Ruiz de Alegría-Fernández de Retana B, Basabe-Barañano N, Fernández-Prado E, Baños-Baños C, Nogales-Rodríguez MA, Echavarri-Escribano M, et al. Quality of life and coping: differences between patients receiving continuous ambulatory peritoneal dialysis and those under hospital hemodialysis. Enfermeria Clinica. 2009;19(2):61-8. PubMed PMID: 105363399. Language: Spanish. Entry Date: 20090731. Revision Date: 20150820. Publication Type: Journal Article.

149. Ruokonen H, Nylund K, Meurman JH, Heikkinen AM, Furuholm J, Sorsa T, et al. Oral symptoms and oral health-related quality of life in patients with chronic kidney disease from predialysis to posttransplantation. Clin Oral Investig. 2019;23(5):2207-13. Epub 2018/10/03. doi: 10.1007/s00784-018-2647-z. PubMed PMID: 30276517.

150. Russo GE, Morgia A, Cavallini M, Centi A, Broccoli ML, Cicchinelli A, et al. [Quality of life assessment in patients on hemodialysis and peritoneal dialysis]. G Ital Nefrol. 2010;27(3):290-5. Epub 2010/06/12. PubMed PMID: 20540022.

151. Ruszkowski J, Heleniak Z, Krol E, Tarasewicz A, Galgowska J, Witkowski JM, et al. Constipation and the Quality of Life in Conservatively Treated Chronic Kidney Disease Patients: A Cross-sectional Study. International Journal of Medical Sciences. 2020;17(18):2954-63. doi: <https://dx.doi.org/10.7150/ijms.49648>. PubMed PMID: 33173416.

152. Ruszkowski J, Heleniak Z, Krol E, Tarasewicz A, Witkowski JM, Debska-Slizien A. Associations between symptoms of constipation and sleep quality in patients with nondialysis chronic kidney disease: a cross-sectional study. Polish Archives Of Internal Medicine. 2021;131(6):512-9. doi: <https://dx.doi.org/10.20452/pamw.15974>. PubMed PMID: 33904290.

153. Salamon K, Woods J, Paul E, Huggins C. Peritoneal dialysis patients have higher prevalence of gastrointestinal symptoms than hemodialysis patients. J Ren Nutr. 2013;23(2):114-8. Epub 2012/05/29. doi: 10.1053/j.jrn.2012.02.007. PubMed PMID: 22633989.

154. Sandoval-Jurado L, Ceballos-Martinez ZI, Navarrete-Novelo C, Gonzalez-Hernandez F, Hernandez-Colin V. [Quality of life in patients with continuous ambulatory peritoneal dialysis]. Rev Med Inst Mex Seguro Soc. 2007;45(2):105-9. Epub 2007/06/07. PubMed PMID: 17550694.

155. Sarhan AL, Jarareh RH, Shraim M. Quality of life for kidney transplant recipients and hemodialysis patients in Palestine: a cross-sectional study. BMC Nephrology. 2021;22(1):1-7. doi: 10.1186/s12882-021-02412-z. PubMed PMID: 150668966. Language: English. Entry Date: In Process. Revision Date: 20210810. Publication Type: journal article. Journal Subset: Biomedical.

156. Sawada A, Hiragi S, Tamura H, Goto R, Matsuyama Y, Sakai K, et al. Evaluation of the Quality of Life and Health-Related Quality of Life of Patients With End-Stage Kidney Disease Resulting From Kidney Transplantation Using the Kidney Disease Quality of Life-Short Form and EuroQOL-5 Dimension-5 Level Questionnaires. Transplantation Proceedings. 2021;53(3):881-4. doi: <https://dx.doi.org/10.1016/j.transproceed.2020.09.018>. PubMed PMID: 33303208.

157. Seguí Gomà A, Amador Peris P, Ramos Alcario AB. Quality of life of patients with chronic kidney disease undergoing renal with dialysis therapy. Revista de la Sociedad Española de Enfermería Nefrológica. 2010;13(3):155-60. PubMed PMID: 105101160. Language: Spanish. Entry Date: 20101029. Revision Date: 20150820. Publication Type: Journal Article.

158. Senthil Kumar TG, Soundararajan P, Maiya GA, Ravi A. Physical Activity and Quality of Life after Renal Transplantation. Online Journal of Health & Allied Sciences. 2018;17(4):1-4. PubMed PMID: 134933910. Language: English. Entry Date: 20190301. Revision Date: 20190409. Publication Type: Article. Journal Subset: Allied Health.

159. Shabany Hamedan M, Mohamad Aliha J. Relationship between immunosuppressive medications adherence and quality of life and some patient factors in renal transplant patients in Iran. Glob J Health Sci. 2014;6(4):205-12. Epub 2014/07/08. doi: 10.5539/gjhs.v6n4p205. PubMed PMID: 24999131; PubMed Central PMCID: PMCPMC4825472.

160. Shah VS, Ananth A, Sohal GK, Bertges-Yost W, Eshelman A, Parasuraman RK, et al. Quality of life and psychosocial factors in renal transplant recipients. Transplant Proc. 2006;38(5):1283-5. Epub 2006/06/27. doi: 10.1016/j.transproceed.2006.03.027. PubMed PMID: 16797282.

161. Shimada H, Sakamoto K, Hori S, Asano T, Suzuki T, Gunji Y, et al. Quality of life after cadaveric renal transplantation from a non-heart-beating donor. Transplant Proc. 2000;32(7):1606-7. Epub 2000/12/20. doi: 10.1016/s0041-1345(00)01453-6. PubMed PMID: 11119857.

162. Smith D, Loewenstein G, Jepson C, Jankovich A, Feldman H, Ubel P. Mispredicting and misremembering: patients with renal failure overestimate improvements in quality of life after a kidney transplant. Health Psychol. 2008;27(5):653-8. Epub 2008/10/01. doi: 10.1037/a0012647. PubMed PMID: 18823192.

163. Tait AS. EXPLORING HOW HOPE AND DEPRESSION IN FORMER DIALYSIS PATIENTS ARE RELATED TO QUALITY OF LIFE POST RENAL TRANSPLANTATION. CANNT Journal. 2017;27(2):20-1. PubMed PMID: 123661251. Language: English. Entry Date: 20170621. Revision Date: 20191029. Publication Type: Article.

164. Theofilou P. Quality of life, mental health and health beliefs: comparison between haemodialysis and peritoneal dialysis patients. Interscientific Health Care. 2011;3(2):171-6. PubMed PMID: 104667151. Language: English. Entry Date: 20110915. Revision Date: 20150711. Publication Type: Journal Article.

165. Theofilou P. Quality of life and mental health in hemodialysis and peritoneal dialysis patients: the role of health beliefs. Int Urol Nephrol. 2012;44(1):245-53. Epub 2011/05/07. doi: 10.1007/s11255-011-9975-0. PubMed PMID: 21547466.

166. Theofilou P. Association of insomnia symptoms with kidney disease quality of life reported by patients on maintenance dialysis. Psychol Health Med. 2013;18(1):70-8. Epub 2012/04/27. doi: 10.1080/13548506.2012.674144. PubMed PMID: 22533530.

167. Trbojevic JB, Nesic VB, Stojimirovic BB. Quality of life of elderly patients undergoing continuous ambulatory peritoneal dialysis. Perit Dial Int. 2001;21 Suppl 3:S300-3. Epub 2002/03/13. PubMed PMID: 11887841.

168. Turkmen K, Guney I, Yazici R, Arslan S, Altintepe L, Yeksan M. Health-related quality of life, depression and mortality in peritoneal dialysis patients in Turkey: seven-year experience of a center. Ren Fail. 2014;36(6):859-64. Epub 2014/03/25. doi: 10.3109/0886022X.2014.899874. PubMed PMID: 24655076.

169. Ujszaszi A, Czira ME, Fornadi K, Novak M, Mucsi I, Molnar MZ. Quality of life and protein-energy wasting in kidney transplant recipients. Int Urol Nephrol. 2012;44(4):1257-68. Epub 2012/01/17. doi: 10.1007/s11255-012-0122-3. PubMed PMID: 22246594.

170. Um EK. Gender role in anxiety, depression and quality of life in chronic kidney disease patients. Pakistan Journal of Medical Sciences. 2020;36(2):251-4. doi: <https://dx.doi.org/10.12669/pjms.36.2.869>. PubMed PMID: 32063969.

171. Valdes Arias C, Montoya MM, Rábano Colino M, Artos Montes Y, Cabello Valle P, De Castro Prieto N, et al. Analysis of the agreement between the evaluation by patients undergoing haemodialysis of their Health-Related Quality of Life (HRQL) and the evaluation of these patients by nursing staff. Revista de la Sociedad Española de Enfermería Nefrológica. 2010;13(4):228-34. PubMed PMID: 104810256. Language: Spanish. Entry Date: 20110325. Revision Date: 20150820. Publication Type: Journal Article.

172. Vigil A. Quality of life of peritoneal dialysis and hemodialysis patients: A pilot comparative study. Dissertation Abstracts International: Section B: The Sciences and Engineering. 2019;80(8):No Pagination Specified. PubMed PMID: 2019-41141-011.

173. Vinaccia S, Quiceno JM. Resilience and health-related quality of life in patients with chronic kidney disease-IRC. Revista Argentina de Clinica Psicologica. 20(3):201-11. PubMed PMID: 2012-00232-002.

174. Webbink D, Martin NG, Visscher PM. Does education reduce the probability of being overweight? Journal of Health Economics. 2010;29(1):29-38. doi: 10.1016/j.jhealeco.2009.11.013. PubMed PMID: WOS:000275587300002.

175. Wiederhold D, Kliem V, Landenberger M. [Symptom experience of patients after allogenic renal transplantation]. Dtsch Med Wochenschr. 2015;140(22):e225-30. Epub 2015/11/05. doi: 10.1055/s-0041-106937. PubMed PMID: 26536651.

176. Wyld ML, Chadban SJ, Morton RL. Improving Our Understanding of Quality of Life in CKD. Am J Kidney Dis. 2016;67(6):820-1. Epub 2016/05/24. doi: 10.1053/j.ajkd.2016.03.412. PubMed PMID: 27211365.

177. Yanishi M, Kawa G, Nakamoto T, Yoshida T, Yoshida K, Mishima T, et al. [Lower Urinary Tract Symptoms and Functions after Renal Transplantation at Our Hospital]. Nihon Hinyokika Gakkai Zasshi. 2015;106(4):249-54. Epub 2016/01/01. doi: 10.5980/jpnjurol.106.249. PubMed PMID: 26717783.

178. Yngman-Uhlin P, Kjellsdotter A, Uhlin F, Edell-Gustafsson U. Sleep Quality, Fatigue, and Health-Related Quality of Life in Patients on Initial Peritoneal Dialysis and Multiple Modalities after Two Years: A Prospective Study. Nephrol Nurs J. 2019;46(6):615-49. Epub 2019/12/25. PubMed PMID: 31872992.

179. Zarifian A. Symptom occurrence, symptom distress, and quality of life in renal transplant recipients. Nephrol Nurs J. 2006;33(6):609-18; quiz 19. Epub 2007/01/16. PubMed PMID: 17219723.

180. Zaydfudim V, Feurer ID, Moore DR, Moore DE, Pinson CW, Shaffer D. Pre-transplant overweight and obesity do not affect physical quality of life after kidney transplantation. J Am Coll Surg. 2010;210(3):336-44. Epub 2010/03/03. doi: 10.1016/j.jamcollsurg.2009.11.009. PubMed PMID: 20193898.

181. Zhang L, Guo Y, Ming H. Effects of hemodialysis, peritoneal dialysis, and renal transplantation on the quality of life of patients with end-stage renal disease. Revista Da Associacao Medica Brasileira. 2020;66(9):1229-34. doi: <https://dx.doi.org/10.1590/1806-9282.66.9.1229>. PubMed PMID: 33027450.

182. Zyoud SH, Daraghmeh DN, Mezyed DO, Khdeir RL, Sawafta MN, Ayaseh NA, et al. Quality of life in Palestinian patients with end-stage renal disease receiving treatment by haemodialysis: a cross-sectional study. Lancet. 2018;391:32-. PubMed PMID: WOS:000426471900033.
