# Supplementary material for: Symptom burden and health-related quality of life in chronic kidney disease: A global systematic review and meta-analysis
Source: PLoS Med. 2022 Apr 6;19(4):e1003954. doi: 10.1371/journal.pmed.1003954 (PMC8985967; doi:10.1371/journal.pmed.1003954)
Supplement: S9 Appendix — (DOCX) [file pmed.1003954.s009.docx]

**Developing a renal-specific item bank: a systematic review.**

**Background and aims**

We will develop a new “item bank” and computerised adaptive test (CAT) electronic patient reported outcome measure (ePROM) and paper-based short form to measure symptom burden and impact on quality of life in chronic kidney disease (CKD). This will address the lack of a valid, reliable, responsive and brief patient reported outcome measure (PROM) for use by the UK renal community.

The need to reduce patient burden when measuring PROMs has led researchers to move away from reliance on classical test theory models, and towards item response theory (IRT) and computer adaptive testing. CATs work by administering questions targeted to each individual’s ability or trait level, by using an adaptive algorithm to administer questions from an item bank that are of direct relevance, and at an appropriate level. Responses from individuals who have answered different questions from the same item bank are scored on the same scale, allowing direct comparison.

Underlying every CAT is a bank of items ‘calibrated’ to an IRT model. An item bank consists of a large number of pre-calibrated questionnaire items covering all relevant aspects of the construct under study.

The aim of this review is to contribute towards the development of the item bank by:

Identifying symptoms and Health-Related Quality of Life (HRQOL) domains important in CKD, and where available the prevalence of those symptoms, across all stages of the disease.

Identifying common symptom and HRQOL domains/items incorporated within existing validated PROMs used in CKD.

**Anticipated or actual start date.**

February 2020.

**Review team members and their organisational affiliations.**

Ben Fletcher

Nicola Anderson

Derek Kyte

Centre for Patient Reported Outcomes Research (CPROR), Institute for Applied Health Research (IAHR), University of Birmingham, UK

**Funding**

The review is funded by Kidney Research UK.

**Review questions**

1. What symptoms and Health-Related Quality of Life (HRQOL) domains are important in CKD, and how do these differ across the different stages of CKD?
2. What is the prevalence of those symptoms?
3. What common symptom and HRQL domains/items are incorporated within existing validated PROMs used in CKD.

**Searches**

The following databases will be searched from January 2000 until present:

Ovid MEDLINE, Ovid PsycINFO and EBSCO CINAHL

Three strategies will be employed to identify:

1. Quantitative studies assessing symptom burden, HRQOL or adverse events in CKD (longitudinal or cross-sectional studies) – update of systematic review investigating symptoms in end-stage renal disease by Murtagh *et al* (2007)(1) and Almutary *et al* (2013)(2). The search will be expanded to include any CKD stage, HRQOL and adverse events.
2. Qualitative studies assessing symptom burden/severity, HRQOL or adverse events in CKD.
3. Studies reporting either the development of a PROM measuring HRQOL and/or CKD symptoms in any CKD population, or evaluation of one or more psychometric properties. We will update the searches conducted in a systematic review of measurement properties of PROMs used in CKD by Aiyegbusi *et al.* (2016)(3)

Forward citation searches and a hand search of the reference lists of included articles will be carried out.

Search strategies for each of the three objectives in OVID Medline are available in the Appendices.

**Types of study to be included.**

All studies must be:

1. Original research articles published in English
2. Involve adults with chronic kidney disease, including: stage 1-5, on dialysis or in receipt of a renal transplant

Studies from search strategy 1 must:

1. Assess quality of life, symptom burden or adverse events as the primary aim of research (report prevalence data on symptoms/impacts on HRQOL)

Studies from search strategy 2 must:

1. Assess patient and healthcare professional perspectives on CKD symptoms, HRQOL or adverse events (data from interviews or focus groups)

Studies from search strategy 3 must:

1. Report either the development, or evaluation of one or more psychometric properties, of a PROM measuring HRQOL and/or CKD symptoms in any CKD population.

Studies will be screened for inclusion independently by two researchers (BF all records, and either DK or NA).

**Exclusions**

1. Editorials, conference abstracts, case reports, systematic reviews
2. Full text not available
3. Articles not in English

**Main outcome(s).**

Symptoms, symptom clusters, HRQOL and adverse events reported in CKD.

Validated PROMs used to monitor symptoms and/or HRQOL in CKD.

**Data extraction (selection and coding).**

Data will be extracted in duplicate all included studies using a pre-piloted data extraction form.

Data will be extracted on:

Study information: year conducted, country of origin, study design, sample size, study design

Study population: inclusion/exclusion criteria, CKD stage, demographic information, treatments

Study outcomes: symptoms/symptom clusters definition (and prevalence if available), QOL domain definitions, experiences/perceptions around CKD HRQOL/symptoms/symptom clusters (from qualitative studies)

PROMs: generic/CKD specific/utility based, mode of administration (self- or interviewer-administered), method of capture (paper-based, telephone, electronic), recall period, number of items, scoring, permissions for use

**Strategy for data synthesis.**

Symptoms/QOL domains/items from PROMs used in CKD research will be extracted and combined into similar categories. Initial categories will be based on those reported in Lockwood (2019)(4) and van der Willik (2019)(5) Categories will be refined and new categories introduced when necessary during data extraction.

Initial symptom/impact categories:

- Neuromuscular (e.g., sore muscles, cramps, joint pain, numbness in hands/feet, muscle spasms, poor mobility, etc)
- Cardiopulmonary (e.g., chest pain, heart palpitation, shortness of breath, feeling faint, etc)
- Gastrointestinal (e.g., constipation, diarrhoea, nauseas, decreased appetite, heartburn, etc)
- Psychological/emotional (e.g., depression, anxiety, nervousness, worry, anger, low motivation, intrusive thoughts, etc)
- Energy/fatigue (e.g., tiredness, difficulty sleeping, feeling weak, waking in the night, etc)
- Skin problems (e.g., dry skin, itchiness, loss of hair, sweating, etc)
- Sexual function (e.g., decreased interest in sex, inability to enjoy sex, difficulty becoming aroused, etc)

Prevalence figures will be combined using meta-analysis, if the heterogeneity is acceptable. Data will be pooled using either the random- or the fixed-effects model depending on the heterogeneity of the included studies. Heterogeneity will be determined using Cochran's Q-test at a significance level of 0.10. I² will be calculated to quantify the heterogeneity; acceptable heterogeneity will be defined as I² <70%. In studies with a high heterogeneity (I² >70%), a random-effects model was used.

Logit transformation of the prevalence figures will be applied as logits are more likely to have a normal distribution, essential for pooling data. The final pooled logit will be back transformed, resulting in pooled prevalence and 95% CIs.

Subgroup analyses will be performed based on the stage of CKD, but only if there are at least three studies in a subgroup.

**Appendices**

OVID Medline search strategy for objective 1 (update of Almutary 2013)

1. (Symptom* AND CKD).ti.

2. (Symptom* AND chronic kidney disease).ti.

3. (Symptom* AND end-stage renal failure).ti.

4. (Symptom* AND (end-stage kidney disease OR kidney disease)).ti.

5. (Symptom* AND (haemodialysis or peritoneal dialysis)).ti.

6. (Symptom burden AND renal failure).ti.

7. (Symptom burden instrument AND kidney failure).ti.

8. 1 OR 2 OR 3 OR 4 OR 5 OR 6 OR 7

9. ((Quality of life OR QOL) AND CKD).ti.

10. ((Quality of life OR QOL) AND chronic kidney disease).ti.

11. ((Quality of life OR QOL) AND end-stage renal failure).ti.

12. ((Quality of life OR QOL) AND (end-stage kidney disease OR kidney disease)).ti.

13. ((Quality of life OR QOL) AND (haemodialysis or peritoneal dialysis)).ti.

14. ((Quality of life OR QOL) AND renal failure).ti.

15. ((Quality of life OR QOL) AND kidney failure).ti.

16. 9 OR 10 OR 11 OR 12 OR 13 OR 14 OR 15

17. (Adverse event* AND CKD).ti.

18. (Adverse event* AND chronic kidney disease).ti.

19. (Adverse event* AND end-stage renal failure).ti.

20. (Adverse event* AND (end-stage kidney disease OR kidney disease)).ti.

21. (Adverse event* AND (haemodialysis or peritoneal dialysis)).ti.

22. (Adverse event* AND renal failure).ti.

23. (Adverse event* AND kidney failure).ti.

24. 17 OR 18 OR 19 OR 20 OR 21 OR 22 OR 23

25. 8 OR 16 OR 24

OVID Medline search strategy for objective 2.

1. Kidney diseases/

2. Kidney disease.ti,ab.

3. CKD.ti,ab.

4. End stage renal disease.ti,ab.

5. End stage renal failure.ti,ab

6. End stage kidney disease.ti,ab

7. End stage kidney failure.ti,ab.

8. *dialysis.ti,ab

9. haemodialysis.ti,ab

10. peritoneal dialysis.ti,ab.

11. 1 OR 2 OR 3 OR 4 OR 5 OR 6 OR 7 OR 8 OR 9 OR 10

12. Symptom*.ti,ab.

13. Symptom burden.ti,ab.

14. Quality of life.ti,ab.

15. QOL.ti,ab.

16. Health related quality of life.ti,ab.

17. HRQOL.ti,ab.

18. (Adverse event* or AE*).ti,ab.

19. 12 OR 13 OR 14 OR 15 OR 16 OR 17 OR 18

20. Qualitative research/

21. Observation.ti,ab.

22. Interview.ti,ab.

23. ((qualitative or semi-structured or semistructured or unstructured or informal or in-depth or indepth or "face-to-face" or structured or guide*) adj3 (interview* or discussion* or questionnaire*)).ti,ab.

24. (qualitative or focus group or story or stories or narration or narrative* or discourse or discursive or grounded theory or ethnogra* or phenomenolog* or fieldwork or field work or key informant*).ti,ab.

25. 20 OR 21 OR 22 OR 23 OR 24

26. 11 AND 19 AND 25

OVID Medline search strategy for objective 3 (update of Aiyegbusi 2017)

1. (HR-PRO or HRPRO or HRQL or HRQoL or QL or QoL).ti,ab.

2. quality of life.mp.

3. (health index* or health indices or health profile*).ti,ab.

4. health status.mp.

5. ((patient or self or child or parent or carer or proxy) adj (appraisal* or appraised or report or reported or reporting or rated or rating or based or assessed or assessment*)).ti,ab.

6. ((disability or function or functional or functions or subjective or utility or utilities or wellbeing or well being) adj2 (index or indices or instrument or instruments or measure or measures or questionnaire* or profile or profiles or scale or scales or score or scores or status or survey or surveys)).ti,ab.

7. ((((patient adj reported adj outcome adj measure*) or patient) adj reported adj outcome*) or capability or capabilities).mp. [mp=title, abstract, original title, name of substance word, subject heading word, floating sub-heading word, keyword heading word, organism supplementary concept word, protocol supplementary concept word, rare disease supplementary concept word, unique identifier, synonyms]

8. 1 or 2 or 3 or 4 or 5 or 6 or 7

9. (Renal replacement therapy or APD or Automated Peritoneal Dialysis or CAPD, Continuous Ambulatory Peritoneal Dialysis or CCPD or Continuous cyclic peritoneal dialysis or dialysis or h*emofiltration or h*emodiafiltration or h*emodialysis or kidney transplant* or predialysis or renal replacement or renal transplant*).mp.

10. (CRF or chronic renal failure or CKF or chronic kidney failure or kidney disease* or renal disease or kidney failure or renal failure or CKD or chronic kidney disease or ESKD or end stage kidney disease or ESKF or end stage kidney failure or ESRF or end stage renal failure or ESRD or end stage renal disease or kidney insufficiency).mp.

11. Renal Insufficiency, Chronic/

12. 9 or 10 or 11

13. (((((((((((((((((((((((((((((Instrumentation or method* or Validation Studies or Comparative Study).mp. or psychometrics/ or psychometr*.mp. or clinimetr*.mp. or clinometr*.mp. or outcome assessment health care/ or outcome assessment*.ti,ab. or outcome measure*.mp. or observer variation/ or observer variation*.ti,ab. or Health Status Indicators/ or reproducibility of results/ or reproducib*.ti,ab. or discriminant analysis/ or reliab*.ti,ab. or unreliab*.ti,ab. or valid*.ti,ab. or coefficient of variation.ti,ab. or coefficient*.ti,ab. or homogeneity.ti,ab. or homogeneous.ti,ab. or internal consistency.ti,ab. or cronbach*.ti,ab.) and alpha*.ti,ab.) or item*.ti,ab.) and correlation*.ti,ab.) or selection*.ti,ab. or reduction*.ti,ab. or agreement.mp. or precision.mp. or imprecision.mp. or precise value*.mp. or test-retest.ti,ab. or test.ti,ab.) and retest.ti,ab.) or reliab*.ti,ab.) and test.ti,ab.) or retest.ti,ab. or stability.ti,ab. or interrater.ti,ab. or inter-rater.ti,ab. or intrarater.ti,ab. or intra- rater.ti,ab. or intertester.ti,ab. or inter-tester.ti,ab. or intratester.ti,ab. or intra- tester.ti,ab. or interobserver.ti,ab. or inter-observer.ti,ab. or intraobserver.ti,ab. or intra-observer.ti,ab. or intertechnician.ti,ab. or inter-technician.ti,ab. or intratechnician.ti,ab. or intra-technician.ti,ab. or interexaminer.ti,ab. or inter- examiner.ti,ab. or intraexaminer.ti,ab. or intra-examiner.ti,ab. or interassay.ti,ab. or inter-assay.ti,ab. or intraassay.ti,ab. or intra-assay.ti,ab. or interindividual.ti,ab. or inter-individual.ti,ab. or intraindividual.ti,ab. or intra-individual.ti,ab. or interparticipant.ti,ab. or inter-participant.ti,ab. or intraparticipant.ti,ab. or intra- participant.ti,ab. or kappa*.ti,ab. or kappa's.ti,ab. or repeatab*.mp. or replicab*.mp. or repeated.mp.) and measure*.mp.) or finding*.mp. or result*.mp. or test*.mp. or generaliza*.ti,ab. or generalisa*.ti,ab. or concordance.ti,ab. or intraclass.ti,ab.) and correlation*.ti,ab.) or discriminative.ti,ab. or known group.ti,ab. or factor analysis.ti,ab. or factor analyses.ti,ab. or factor structure.ti,ab. or factor structure.ti,ab. or dimension*.ti,ab. or subscale*.ti,ab. or multitrait.ti,ab.) and scaling.ti,ab. and analysis.ti,ab.) or analyses.ti,ab. or item discriminant.ti,ab. or interscale correlation*.ti,ab. or error.ti,ab. or errors.ti,ab. or individual variability.ti,ab. or interval variability.ti,ab. or rate variability.ti,ab. or variability.ti,ab.) and analysis.ti,ab.) or value*.ti,ab. or uncertainty.ti,ab.) and measurement.ti,ab.) or measuring.ti,ab. or standard error of measurement.ti,ab. or sensitiv*.ti,ab. or responsive*.ti,ab. or limit*.ti,ab.) and detection.ti,ab.) or minimal detectable concentration.ti,ab. or interpretab*.ti,ab. or minimal.ti,ab. or minimally.ti,ab. or clinical.ti,ab. or clinically.ti,ab.) and important.ti,ab.) or significant.ti,ab. or detectable.ti,ab.) and change.ti,ab.) or difference.ti,ab. or small*.ti,ab.) and real.ti,ab.) or detectable.ti,ab.) and change.ti,ab.) or difference.ti,ab. or meaningful change.ti,ab. or ceiling effect.ti,ab. or floor effect.ti,ab. or Item response model.ti,ab. or IRT.ti,ab. or Rasch.ti,ab. or Differential item functioning.ti,ab. or DIF.ti,ab. or computer adaptive testing.ti,ab. or item bank.ti,ab. or cross-cultural equivalence.ti,ab.

14. (PRO integration or Clinical PRO application* or telePRO or automated PRO algorithm* or screening purpose* or PRO questionnaire* or Patient-reported outcome questionnaire* or Patient-reported symptom* or Patient-centred care or Patient self- report* or Self-report health or Self-rated health or Self-reported measure* of health or Health outcome* or Health communication* or Hospital performance evaluation* or Automated telephone survey system* or paper-based survey* or web-based survey* or web-based PRO platform* or web-based system* or PRO collection* or PRO measure* or PRO intervention* or PRO assessment intervention* or PRO data or PRO assessment* or Routine PRO assessment* or Routine PRO collection or Symptom assessment* or Symptom monitoring or Symptom data or Functional status or Electronic PRO assessment* or Electronic PRO system* or ePRO or ePRO* or ePRO system* or PRO system* or Generic PRO system* or PRO-based clinical alert system* or PROM or eRPOM or electronic PROM).mp.

15. 13 or 14

16. 8 and 12 and 15

**References**

1. Murtagh FE, Addington-Hall J, Higginson IJ. The prevalence of symptoms in end-stage renal disease: a systematic review. Adv Chronic Kidney Dis. 2007;14(1):82-99.

2. Almutary H, Bonner A, Douglas C. Symptom burden in chronic kidney disease: a review of recent literature. J Ren Care. 2013;39(3):140-50.

3. Aiyegbusi OL, Kyte D, Cockwell P, Marshall T, Gheorghe A, Keeley T, et al. Measurement properties of patient-reported outcome measures (PROMs) used in adult patients with chronic kidney disease: A systematic review. PLoS One. 2017;12(6):e0179733.

4. Lockwood MB, Chung S, Puzantian H, Bronas UG, Ryan CJ, Park C, et al. Symptom Cluster Science in Chronic Kidney Disease: A Literature Review. Western Journal of Nursing Research. 2019;41(7):1056-91.

5. van der Willik EM, Meuleman Y, Prantl K, van Rijn G, Bos WJW, van Ittersum FJ, et al. Patient-reported outcome measures: selection of a valid questionnaire for routine symptom assessment in patients with advanced chronic kidney disease - a four-phase mixed methods study. BMC Nephrol. 2019;20(1):344.
