# Supplementary material for: Symptom burden and health-related quality of life in chronic kidney disease: A global systematic review and meta-analysis
Source: PLoS Med. 2022 Apr 6;19(4):e1003954. doi: 10.1371/journal.pmed.1003954 (PMC8985967; doi:10.1371/journal.pmed.1003954)
Supplement: Attachment — Submitted filename: Response to editors BF01022022.docx [file pmed.1003954.s012.docx]

Requests from the editors:

1. Please completely address the remaining points of Reviewer 2.

**Author response:** we have addressed the comments in detail below.

2. Abstract: Methods and Findings: Please explicitly describe the participants/comparator groups among the patients with CKD included.

**Author response:** we have now addressed this in the abstract.

3. Abstract: Methods and Findings: Please mention the types of/any restrictions on design of studies included (e.g. longitudinal, cross-sectional).

**Author response:** we have now addressed this in the abstract.

4. Abstract: Line 43-44: Please mention how (the specific tools used) methodological quality and risk of bias were assessed.

**Author response:** we have now addressed this in the abstract.

5. Abstract: For the main outcomes described, it would be helpful to note the numbers of studies/patients included for each.

**Author response:** we have now addressed this in the abstract.

6. Abstract: Line 49: Please define the abbreviation “RRT” at first use.

**Author response:** we have now addressed this in the abstract.

7. Abstract: Line 51-52: Please mention the comparison group for this analysis, and please report 95% CIs and p values for each symptom mentioned.

**Author response:** we have now addressed this in the abstract.

8. Abstract: Lines 54-58: Please also report the p values for these comparisons.

**Author response:** we have now addressed this in the abstract.

9. Methods: Line 124-125: Please reference the supporting information file that contains the study protocol.

**Author response:** we have now added ‘(S9 Appendix – Review Protocol)’.

10. Methods: Please mention the types of studies/designs included. The search did not include non-English language sources of studies (45 studies excluded for this reason). Please acknowledge and discuss this as a limitation.

**Author response:** we have updated our inclusion/exclusion details to address this point and have also updated the ‘strengths and limitations’ section accordingly.

11. Methods: Please provide complete details for the fixed and random effects models used. Please indicate for which analyses fixed vs. random effects models were used.

**Author response**: we have clarified that random effects models were used for all analyses (lines 170-171), and added reference to the Stata commands used for prevalence and severity data (lines 174-176)

12. Discussion: Line 381, Line 461: Please temper claims of primacy with “To the best of our knowledge” or similar.

**Author response:** thank you, we have updated the discussion accordingly.

13. Table 1: Please note that “Population” indicates stage of CKD. Please indicate in the table that values are number of studies and (%) if accurate. It may be helpful to include a legend for the table, defining all abbreviations (RRT) in the legend.

**Author response:** Table 1 has now been updated to address this comment.

14. Table 3: Please clarify the values presented in the table.

**Author response:** we have clarified the values in the table legend.

15. Figure 4: Please include a legend describing all abbreviations used.

**Author response:** we have added a legend describing all abbreviations.

16. S3 Appendix, S6 appendix, S7 appendix: Please include a legend for this table, defining all abbreviations and color-coding used. We suggest choosing a color-coding scheme that avoids the need to distinguish red and green.

**Author response:** we have now removed or amended colour coding and we have either removed or defined all abbreviations across appendices.

17. S8 Appendix: Separate titles and legends for each item (each tab) in this file would be helpful to understand which comparisons are being shown and what is illustrated in each table.

**Author response:** we have added a title to each tab and legend to define highlighted cells.

18. PRISMA Checklist: Thank you for including the PRISMA checklist. Please use section and paragraph numbers to refer to locations within the text. Please do not use page numbers or line numbers.

**Author response:** we have updated the PRISMA checklist as requested.

Comments from the reviewers:

Reviewer #1: The authors have satisfactorily responded to each comment in turn, amending the manuscript as required.

**Author response:** we thank reviewer #1 for their important input.


Reviewer #2: Thank you for the opportunity to review this revised manuscript describing the symptom burden and health related quality of life in CKD. 
I do think this revision is improved but is not yet ready for publication. The authors have addressed many of my concerns but did not directly respond to the second paragraph of text of my original review. Some concerns were addressed in separate comments but I am still concerned that the discussion is largely underdeveloped. It lacks synthesis and interpretation of the study's findings and largely repeats the study's findings, which is particularly apparent in the second paragraph of the discussion… The entire discussion could be strengthened by including more interpretation.

**Author response:** we thank reviewer #2 for their detailed comments. We have extensively reworked the discussion to address these valid points.

The third paragraph of the discussion also contains too many details about the cited study - please only highlight main similarities.

**Author response:** thank you, we have updated this paragraph accordingly.

Other concerns
- Methods: I am concerned about the psychometric validity of rescoring the the self-reported scores to 100. Are there any previous studies or citations that have reported rescaling of the scores to 100?

**Author response:** thank you. This method has been used before: Han CJ, Yang GS, Syrjala K. Symptom Experiences in Colorectal Cancer Survivors After Cancer Treatments: A Systematic Review and Meta-analysis. Cancer Nurs. 2020 May/Jun;43(3):E132-E158. doi: 10.1097/NCC.0000000000000785. PMID: 32000174; PMCID: PMC7182500. The authorship group deliberated for quite some time on the best method of presenting the severity data. We concluded that random effects meta-analysis of the adjusted severity scores represented an appropriate method of generating a distribution which allowed estimation of population differences with a quantifiable degree of probability. However, we do accept severity score transformation as a limitation within the study and as such we have highlighted this within the limitations section of the discussion.

- Figures - please clarify what the word prevalence indicates ? Is it the % of those included in each of the studies that reported the symptom? Please provide that explanation in the Figure legend.

**Author response:** thank you, we have clarified the language across all figure legends in response to this comment.
