# Supplementary material for: Symptom burden and health-related quality of life in chronic kidney disease: A global systematic review and meta-analysis
Source: PLoS Med. 2022 Apr 6;19(4):e1003954. doi: 10.1371/journal.pmed.1003954 (PMC8985967; doi:10.1371/journal.pmed.1003954)
Supplement: Attachment — Submitted filename: Response to editors BF21022022.docx [file pmed.1003954.s013.docx]

Dear editors,

We have addressed all of the requested edits with the exception of:

12 Figure 4: It would be helpful if the categories could be made to stand out more easily – we were unable to do this given the constraints of the software used.

We have submitted a marked and clean version of the manuscript.

Best wishes,

Ben Fletcher on behalf of all co-authors.
