# Supplementary material for: Symptom burden and health-related quality of life in chronic kidney disease: A global systematic review and meta-analysis
Source: PLoS Med. 2022 Apr 6;19(4):e1003954. doi: 10.1371/journal.pmed.1003954 (PMC8985967; doi:10.1371/journal.pmed.1003954)
Supplement: Attachment — Submitted filename: PLOS Medicine response to reviewer comments 30Nov21.docx [file pmed.1003954.s011.docx]

Dear Editor,

Thank you for giving us the opportunity to submit a revised draft of our manuscript “Symptom burden and health-related quality of life in chronic kidney disease: a global systematic review and meta-analysis”. We are grateful to the editor and reviewers for their insightful comments on the paper. We have made the suggested changes, and detail our responses below. We have highlighted the areas of change in red font in the manuscript.

Regards

Dr Ben Fletcher and Dr Derek Kyte on behalf of all authors.

Requests from the editors

1. Please completely address all reviewer comments, particularly the concerns mentioned by Reviewer 2 regarding reporting of the data extraction and analysis methods, and assessment of heterogeneity, including how the high degree of heterogeneity impacts pooling across studies in terms of prevalence and severity, and the interpretation of the meta analyses.

**Author response:** Thank you, we have addressed all reviewer comments and detail our responses to each point raised below.

2. Please update the search to the present time (currently through February of 2020 and updated through October 2020).

**Author response:** Thank you, we updated the search through to 1^st^ September 2021, the latest possible date to allow revision of the manuscript (including updated meta-analysis) by the re-submission deadline.

3. Abstract: Please report your abstract according to PRISMA for abstracts, following the PLOS Medicine abstract structure (Background, Methods and Findings, Conclusions)[http://www.plosmedicine.org/article/info:doi/10.1371/journal.pmed.1001419](http://www.plosmedicine.org/article/info:doi/10.1371/journal.pmed.1001419) .

**Author response:** Thank you, we have re-structured the abstract as requested.

Please combine the Methods and Findings sections into one section, “Methods and findings”. Please provide the dates of search, types of study designs included, exclusion criteria including language exclusions, and synthesis/appraisal methods. Please mention how you evaluated study quality and risk of bias, including publication bias.

**Author response:** Thank you, we have re-structured the abstract as requested.

4. Abstract: Methods and Findings: Please present quantitative data in support of the main findings of the meta analysis. As the last sentence of the Methods and Findings, please describe the main limitation(s) of the study's methodology.

**Author response:** Thank you, we have re-structured the abstract as requested.

5. Author summary: At this stage, we ask that you include a short, non-technical Author Summary of your research to make findings accessible to a wide audience that includes both scientists and non-scientists. The Author Summary should immediately follow the Abstract in your revised manuscript. This text is subject to editorial change and should be distinct from the scientific abstract. Please see our author guidelines for more information:[https://journals.plos.org/plosmedicine/s/revising-your-manuscript#loc-author-summary](https://journals.plos.org/plosmedicine/s/revising-your-manuscript#loc-author-summary)

**Author response:** Thank you, we have added the author summary as requested.

6. Main text: For citations of references in the text, please place reference numbers within square brackets placed before the sentence punctuation. For multiple references, please do not include spaces within brackets.

**Author response:** Thank you, we have updated the formatting and placement of references.

7. Main text: Please include line numbers with the revised version.

**Author response:** Line numbers have been added.

8. Methods: Thank you for noting that your study protocol was registered. Please note whether the protocol and analysis methods were developed prospectively. Please make sure that the Methods section transparently describes when analyses were planned, and when/why any data-driven changes to analyses took place.

**Author response:** Thank you, we have now addressed this in the methods section, and included the protocol in S9 Appendix.

9. Methods: Please provide a list of excluded studies and reasons for exclusion.

**Author response:** We have included the list of studies excluded after full text screening in S5 Appendix, and the reasons are summarised in the PRISMA diagram (Figure 1).

10. Methods: Please note how publication bias was assessed.

**Author response:** We included the following description in the methods: “Publication bias was assessed where meta-analyses included 10 or more studies using Eggers test for funnel plot asymmetry, with a significance level of p<0.05.”

11. Results: As mentioned by Reviewer 1, please include a table with each study summarizing the design, instruments used, outcome measures, size, etc. and include quality assessment and risk of bias for each.

**Author response:** We have compiled the table as requested in S3 Appendix – Information on included studies.

12. Results: Please provide an assessment of how results differ by excluding studies judged to be at high risk of bias.

**Author response:** We used the Joanna Briggs Institute Critical Appraisal Checklist for Studies Reporting Prevalence data, and overall, we show that the included studies scored well in most categories. The Joanna Briggs Institute does not recommend an overall score, or indeed a cut-off score that we could use to conduct sensitivity analyses based on study quality, nor have we found any published literature recommending an appropriate cut-off for systematic reviews of observational studies. Therefore any cut-off we employed would be purely subjective. On that basis, after discussion with the authorship team, we have concluded that it would not be informative to divide studies for a sensitivity analysis according based on risk of bias.

13. Results: Meta-analysis for fatigue prevalence across CKD levels: Please provide both p values and 95% CIs for comparisons of fatigue prevalence between groups, in the text as well as in Figure 3.

**Author response:** Thank you, these values have been added to the text. It is not possible to include these p values in the forest plot, however, all FDR sharpened q-values for each comparison have now been included in the S6 and S7 Appendices.

14. Discussion: Please present and organize the Discussion as follows: a short, clear summary of the article's findings; what the study adds to existing research and where and why the results may differ from previous research; strengths and limitations of the study; implications and next steps for research, clinical practice, and/or public policy; one-paragraph conclusion.

**Author response:** Thank you, we have re-structured the discussion as requested.

15. Figure 4: Please provide an X axis label. The individual symptoms are difficult to read on the left, please increase the font size. Please provide the meta-analysis results for each symptom shown in Figure 4 as displayed for Figure 3.

**Author response:** We have now included an x-axis label and increased the text size as much as possible. All meta-analyses across groups are summarised in Table 2, and further detail in S6 and S7 Appendices. Pairwise comparisons of groups are included in S8 Appendix.

16. PRISMA diagram: Please note the “other sources” for the 8 additional records identified. Please note the reasons for records excluded at each stage.

**Author response:** We have now included the PRISMA diagram in the manuscript, rather than as an appendix. We have detailed the source of the additional studies in the text, and the reasons for exclusions at full text stage Figure 1. We have included a list of studies excluded at full text stage in S5 Appendix.

17. PRISMA Checklist: When completing the checklist, please use section and paragraph numbers, rather than page numbers.

**Author response:** We have updated the checklist to use page numbers and line numbers, and included in S1 Appendix.

Reviewer #1

The stated aim of this review is to produce a comprehensive and consolidated synthesis of symptom prevalence/severity and HRQOL across CKD stage and treatment groups.

Comments:

"The Preferred Reporting Items for Systematic Reviews and Meta-Analyses (PRISMA) guidelines were followed (PRISMA checklist, supplementary appendix 1) and the study protocol was registered with PROSPERO (CRD42020164737)."
The authors have suitably provided the PRISMA checklist in the supplementary material. 
Can they also please provide a copy of the study protocol in the supplementary material?

**Author response:** Thank you, the protocol has now been included in S9 Appendix.

"Three authors (BF, SD, DK) independently assessed selected articles for eligibility at the title/abstract and full-text stages."
Can the authors please confirm if the articles were triple checked, or were they divided up between the three assessors?
Were there any cases of disagreement?  If so, how were these resolved?
Also, were the authors of similar expertise and experience?

**Author response:** Articles were double checked by two of three reviewers (BF, SD, DK), we have now clarified this in the text. The reviewers were indeed of similar levels of expertise and experience.

"The following data were independently extracted into a pre-piloted spreadsheet by three authors (BF, SD, DK)..."
As for the previous comment, can the authors please provide similar clarifications for the data extraction process?

**Author response:** Data from included articles were independently extracted by two of three authors. We have now clarified this in the text.

"Symptom prevalence data, and mean HRQOL scores were combined using random-effects meta-analysis. To aid comparison of symptom severity data provided across different outcome measures, all mean severity scores were converted to a 0-100 scale, where a higher score indicates greater severity. For HRQOL scores, 100 represents best possible quality of life. Symptom severity scores were also combined using random effects meta-analysis. A weighted composite summary score (Kidney Summary Score or KSS) for the Kidney Disease Quality of Life (KDQOL) instrument was calculated by combining the 'Symptom/Problems', 'Effects' and 'Burden' domains, using the method reported by Peipert et al. (2019). Exploratory subgroup analysis was used to compare prevalence and score (severity and HRQOL) estimates between groups in meta-analyses (predialysis v dialysis, predialysis v transplant and dialysis v transplant). To account for multiple testing, sharpened False Discovery Rate (FDR) q-values were
computed, and adjusted p-values are reported."
and
"Random effects meta-analyses were used to pool data on symptom prevalence and severity, and HRQOL scores. We stratified meta-analyses by CKD status: Stage 1-5 not on dialysis (predialysis), on dialysis, and post renal transplantation."
The authors have applied a technically appropriate and rigorous statistical methodology.

Can the authors please discuss heterogeneity within the methods, results and conclusions?
It is noted that I2 in Figure 3 is > 95%, which is substantially high and may mean the results of the meta-analysis are misleading.

**Author response:** Thank you for highlighting this. We have added more detail to the methods, results and discussion sections to clarify our approach (as outlined in our protocol which now appears in the supplementary appendix). We followed established guidelines for systematic reviews of observational epidemiological studies reporting prevalence data published by Munn et al (2015). In summary, heterogeneity was determined using Cochran’s Q test at a significance level of 0.10. I^2^ was calculated to quantify heterogeneity (acceptable heterogeneity defined as I^2^ <70%). In analyses with high heterogeneity, a random effects model was used. Subgroup analysis was performed based on the stage of CKD (not on RRT versus dialysis versus transplant), but only if there were 3 or more studies within a subgroup.

Can the authors confirm whether Figure 6 and Table 4 show the same thing?
If so, it is suggested to remove one of these to avoid duplication.

**Author response:** Thank you, we have now removed Table 4.

It is also suggested to replace the List of Included studies in Appendix 3, with a table describing each (type, size, dates, location etc.) study with its associated JBI quality assessment.

**Author response:** Thank you, we have now included this list in S3 Appendix.

Reviewer #2:

Thank you for the opportunity to review this manuscript describing the symptom burden and health-related quality of life in CKD using a systematic review and meta-analysis of the published literature 2000 to Feb 2020. The authors used three main search engines to retrieve the publications (MEDLINE, PschINFO, CINAHL), which yielded 415 studies to include in the review and meta-analysis. This is an interesting number since only 4 years before, a systematic review published in PLOS One (https://journals.plos.org/plosone/article?id=10.1371/journal.pone.0179733) only identified 66 publications using an additional search engine (EMBASE) that covered the same patient-reported outcome measures in CKD.

**Author response:** Thank you. Our study search strategy and inclusion/exclusion criteria differed from the PLOS one systematic review (in which 3,702 titles/abstracts were screened), which was restricted to papers that reported either the development or evaluation of one or more psychometric properties of a PROM. We had no such restriction in our review, hence the larger pool of studies.

I have major concerns regarding the methodology that was used - particularly for meta-analyses, largely regarding pooling of the data across study populations that used different study instruments without formal assessment of heterogeneity that would indicate if it would be worthwhile to pool the results. The authors already note in that the outcome measures (pg 8) there was "little consistency across measures". The methods section is very under developed and the reader cannot clearly understand the methods used. Additionally, I have concerns about description of prevalence and severity of symptoms as the true denominator is unknown and is affected by publication bias and ascertainment bias (e.g., more studies and instruments asked about pain and fatigue rather than itching - does that mean that itching is less important to the patients?). The discussion is also under-developed.  I think the paper would be strengthened by reporting the number of studies/instruments that included particular symptom measures, what the typical reported level of that symptom is across stages of CKD (if possible) without formal analyses since the ability to pool is likely not sufficient. 

Throughout manuscript - please use alternative term for "pre-dialysis" - for example, non-dependent CKD as not all patients with CKD will go on to develop dialysis

**Author response:** Thank you. We have replaced this term throughout with ‘patients not on renal replacement therapy’

Abstract
-Background: clarify what "treatment groups" means and what does inform capture mean?
-Methods: indicate end year of search
-Conclusion: why only highlight the systematic review portion

**Author response:** Thank you, we have amended the abstract to address these comments.

Introduction
-Spelling error for vulnerable

**Author Response:** Thank you. Error now corrected.

Methods: [2 or more]
-Why was EMBASE not included?

**Author Response:** Thank you. We felt EMBASE would have a significant overlap with MEDLINE and would not add to our already robust search strategy. We included three databases that previous experience has shown to have a broad scope.

-Formal tests of heterogeneity?

-Data analysis section - underdeveloped and need more elaboration of how the studies were pooled and how the instruments were harmonized for pooling - for example - if there was any mention of fatigue in the PROM - was it "counted" as a measure of fatigue? - again how was severity of that measure scored?

- need to also describe how mean HRQOL scores were determined across different instruments - were these also standardized?

-How was a severity score determined?

-How was the weighted composite summary score constructed - need more elaboration than a prior citation.

-Suggest that instead of exploratory meta-analyses comparing scores across stages of CKD - first describe the differences

**Author Response:** Thank you, we agree that the methods section required more information, which we have now added. We followed established guidelines for systematic reviews of observational epidemiological studies reporting prevalence with a focus on estimating the global burden of disease (Munn et al. (2015). We have now made this clear and have added detail around each of the methodological queries outlined above. Exploratory subgroup analysis was used to compare prevalence and score (severity and HRQOL) estimates between groups in meta-analyses (not on RRT versus dialysis, not on RRT versus transplant and dialysis versus transplant). Again, we have made this clearer in the text.

Figure 1 - I think they are misleading - I think is affected by publication bias - is it really symptom prevalence  or prevalence of symptoms asked prior studies?

**Author Response:** Thank you. We have now included the results of Egger’s tests for funnel plot asymmetry to explore publication bias. Of 32 symptom prevalence meta-analyses, three were found to have significant funnel plot asymmetry. None of the HRQOL meta-analyses showed evidence of publication bias.

Figure 2 - it very difficult to interpret these without learning more about the severity scores

**Author Response:** We have now added more detail in the methods section regarding the methods used to pool severity scores.

Figure 4 - as above for figure 2 - need more description about severity scores.

**Author Response:** We included Figure 4 (now Figure 5) to allow readers to see, all in one place, the prevalence of all symptoms across all groups.

Table 3- was individual level used to pool these results or are they from averages (means) reported in the published papers?

**Author Response**: No individual level data were used in pooled analyses, as these were not reported in any of the included studies. We have clarified this in the text.

-The authors note that "data from non-CKD control populations are sparse" which is not possible as there are many many studies that have used legacy PROMS, including SF-36 in non-CKD populations that can provide normative data for comparison <https://hqlo.biomedcentral.com/articles/10.1186/s12955-017-0625-9>

**Author Response:** Thank you for highlighting this. There was a lack of clarity around our intended meaning here. We actually attempted to extract control data that was collected from contemporaneous non-CKD control populations (ideally matched data). We have made this clearer in the text throughout.

Discussion
-I think it is misleading to report there was lower prevalence of symptoms in transplant compared to non-dialysis CKD or dialysis since the denominator differs so much, there were fewer studies and the outcome measures could have asked fewer questions

**Author response:** Thank you. We have clarified the text throughout the manuscript regarding the prevalence of symptoms in transplant patients. There were 139 studies including transplant patients, compared to 126 in patients not on RRT and 274 involving patients on dialysis. After correcting for multiple testing, the following symptoms were significantly less prevalent in the transplant population when compared to those patients not on RRT or receiving dialysis: muscle weakness, fatigue, poor sleep, itching, decreased appetite, depression, dry mouth, poor mobility (FDR sharpened q-values 0.005-0.037). As we corrected for multiple testing, we can be confident that these represent ‘true’ differences between groups.

-On page 29 - "we found little consistently in the PROMS used to capture data around symptoms in CKD" - how do the authors justify meta-analyzing the data?

**Author Response:** Thank you. As mentioned above, we followed established guidelines for systematic reviews of observational epidemiological studies reporting prevalence (Munn et al. (2015), which suggest that, in the presence of significant heterogeneity, random effects meta-analysis may be an appropriate method of generating a distribution which allows estimation of population differences with a quantifiable degree of probability. We have tried to make this clearer in the methods section and also in the limitations section within the discussion.

Suggest the authors include the following citation in their writing about the use of IRT and CAT to develop new measures - as this has been developed by the PROMIS program using mixed methods -<https://www.healthmeasures.net/explore-measurement-systems/promis>

**Author Response:** Thank you for this suggestion. We have incorporated this into the discussion.

The introduction provides background on how this will inform ePROs - I didn't see further discussion on this - which surveys collected were collected with digital/electronic means?

**Author Response:** Thank you. We have removed the element on ePROs in the introduction on the advice of reviewer 1/3.

Reviewer #3

Thank you for the opportunity to review this paper.

It has clearly involved a considerable amount of work to synthesise the mass of data, for which the authors deserve credit.

My main comments concern the framing of the work, some commentary of the methods and then a view on the conclusions reached. This is an important area that requires focus.

The introduction produces the background and rationale for the work but the section on electronic reporting seemed redundant. It was not part of the study. It conflates the definitions of symptoms and the tools used with the operational method to collect those tools. So it did not seem to add anything to the objectives or the methodology.

**Author Response:** Thank you. We have removed the element on ePROs in the introduction

The introduction lays out three clear objectives for the work 'The aim of this study was to: (i) produce a comprehensive and consolidated synthesis of symptom prevalence/severity and HRQOL across CKD treatment groups; (ii) explore which symptoms/HRQOL domains are modified by CKD and may be attributable to the disease; (iii) determine which PRO measures (PROMs) are currently available to capture symptom prevalence/severity and HRQOL in CKD.'

It was not clear how these objectives were achieved from the discussion. It would be useful to reflect back those challenges as a summary of the study.

**Author Response:** Thank you for highlighting this. We have amended the manuscript to strengthen the link between our stated objectives and the summary of findings in the discussion.

Turning to the data itself, there was some discussion on control groups but again it was difficult to pick out the narrative of symptom evolution from normality through to CKD (and across the stages of CKD) to renal replacement therapy (dialysis and transplantation). Figure 4 helps in one sense (and is the fatigue line missing the mark for the dialysis population?) but it only covers prevalence.

The burden comes from both prevalence and from severity. A discussion on this two dimensional view of symptom burden would be valuable. That would provide insight into the utility of measures as drivers to improve care for people with kidney disease.

**Author Response:** Thank you, we have now addressed this.

A minor note on the methods - it was unclear whether those reviewing the papers did so independently and then a consensus was reached where needed.

**Author Response:** Thank you. As per earlier comments, we have now clarified this in the text.

There is also a typo on manuscript page 3 line 10.

**Author Response:** Thank you, we have corrected this**.**
